# Nine new species of *Uramya* Robineau-Desvoidy (Diptera: Tachinidae) from Area de Conservación Guanacaste in northwestern Costa Rica, with a key to their identification

**DOI:** 10.3897/BDJ.5.e9649

**Published:** 2017-03-07

**Authors:** AJ Fleming, D. Monty Wood, M. Alex Smith, Winnie Hallwachs, Daniel Janzen, Tanya Dapkey

**Affiliations:** 1 Agriculture Agri-Food Canada, Ottawa, Canada; 2 Department of Integrative Biology and the Biodiversity Institute of Ontario, Guelph, Canada; 3 University of Pennsylvania, Philadelphia, United States of America

**Keywords:** tropical rain forest, tropical dry forest, cloud forest, parasitoid flies, host-specificity, caterpillars, ACG, Dexiinae, Uramyiini

## Abstract

**Background:**

We describe nine new species in the genus *Uramya* Robineau-Desvoidy, 1830 from Area de Conservación Guanacaste (ACG) in northwestern Costa Rica. All species were reared from an ongoing inventory of wild-caught caterpillars spanning a variety of families (Lepidoptera: Erebidae; Limacodidae; Megalopygidae; Lasiocampidae and Dalceridae). Our study provides a concise description of each new species using morphology, life history, molecular data, and photographic documentation. In addition to the new species the authors provide a redescription the previously described *Uramya sibinivora* Guimarães, which was also collected within ACG during this study. We also provide a redescription of the genus, and a revised key to species of *Uramya* occurring in Central and South America.

**New information:**

The following nine new species of *Uramya*, all authored by Fleming & Wood, are described: *Uramya albosetulosa* Fleming & Wood **sp. nov.**, *Uramya constricta* Fleming & Wood **sp. nov.**, *Uramya contraria* Fleming & Wood **sp. nov.**, *Uramya infracta* Fleming & Wood **sp. nov.**, *Uramya lativittata* Fleming & Wood **sp. nov.**, *Uramya lunula* Fleming & Wood **sp. nov.**, *Uramya nitida* Fleming & Wood **sp. nov.**, *Uramya pannosa* Fleming & Wood **sp. nov.**, and *Uramya penicillata* Fleming & Wood **sp. nov.**

The following are proposed by Wood as new synonyms of *Uramya*: *Olinda* Townsend, **syn. nov.** and *Procleonice* Townsend, **syn. nov.** The following new combination is proposed as a result of the new synonymies: *Uramya brasiliensis* Macquart, **comb. nov.** *Procleonice prolixa* Townsend is synonymized under *Uramya brevicauda* Curran, **syn. nov.**

## Introduction

Tachinidae are the second most diverse family of Diptera (Belshaw 1993, O’Hara 2008), with almost 10,000 described species classified into over 1500 genera (O’Hara 2008, O’Hara 2014). The number of named species catalogued by Guimaraes (1971) for the Neotropical Region, some 2,864 species, is larger than that of any other region. Based on what has recently been discovered in Costa Rica and what is already present in other collections, this number is undoubtedly just a small fraction of what actually exists in nature. The most speciose genera of the Costa Rican tachinid fauna seem to occur in the upper elevations and cloud forests that extend from the western slopes of the Sierra Madre Occidental in Mexico to both slopes of the Andes, from Colombia south to Bolivia. The present study describes 9 new Neotropical species of *Uramya* Robineau-Desvoidy, 1830 (Dexiinae: Uramyini) from Area de Conservación Guanacaste (ACG) in northwestern Costa Rica (http://www.acguanacaste.ac.cr) and provides a key to their identification and that of their Central and South American congeners.

The last major taxonomic work on the Uramyini was by Guimarães (1980): his "Revision of the South American Uramyini (Diptera, Tachinidae)" provided a concise and complete diagnosis of the tribe and included two nominal genera: *Uramya* and *Thelairaporia* Guimarães, 1980. Robineau-Desvoidy (1830) erected the genus *Uramya* based on one male collected in Brasil, misidentified as a female, which he named *U. producta* Robineau-Desvoidy, 1830. Like other genera within the Uramyiini (e.g., *Itaplectops* Townsend, 1927 and *Thelairaporia*), species of *Uramya* parasitize caterpillars within the Arctiinae (Erebidae), Limacodidae, Megalopygidae, Lasiocampidae, and Dalceridae (Arnaud 1978, Guimarães 1980, Wood and Zumbado 2010).

All flies and rearing information described here derive from the ongoing inventory of the tri-trophic relationships between caterpillars, their food plants and their parasitoids within the dry, rain, and cloud forests of the terrestrial portion of ACG (Smith et al. 2006, Smith et al. 2007, Smith et al. 2008, Janzen et al. 2009, Janzen and Hallwachs 2011, Smith et al. 2012, Rodriguez et al. 2012, Fleming et al. 2014b, Fleming et al. 2015b, Janzen and Hallwachs 2015). Since 1978 this inventory has yielded an unprecedented amount of invaluable information on the tri-trophic relationships between parasitoids, hosts and associated food plants (Janzen et al. 2009, Janzen and Hallwachs 2011, Janzen and Hallwachs 2015, Fernandez-Triana et al. 2014).

Our descriptions of these nine new species of *Uramya* build on existing knowledge and are based on differences in external morphology, COI (coxI or cytochrome *c* oxidase I) gene sequences, and male terminalia (when necessary). As the inventory is continually growing, it should be noted that this paper should not be taken as an indication of the final total number of species of *Uramya* present in ACG or Costa Rica. Our descriptions are limited only to the species known and reared from ACG. This paper on *Uramya* is part of a larger effort to describe new species reared during the ACG inventory (Fleming et al. 2014a, Fleming et al. 2014b, Fleming et al. 2015a, Fleming et al. 2015c, Fleming et al. 2015b, Fleming et al. 2015d, Fleming et al. 2016a, Fleming et al. 2016b). This series of taxonomic papers will represent a baseline for later, detailed ecological and behavioral accounts and studies extending across ACG ecological groups, whole ecosystems, and taxonomic assemblages much larger than a genus.

## Materials and methods

### Project aims and rearing intensity

All reared specimens were obtained from host caterpillars collected in ACG (Janzen et al. 2009, Janzen and Hallwachs 2011, Janzen and Hallwachs 2015). ACG's 125,000+ terrestrial hectares span the provinces of Alajuela and Guanacaste, along the dry forested northwestern coast of Costa Rica and inland to the Caribbean lowland rain forest. ACG comprises several different biomes and intergrades, ranging from sea level up to 2,000 m. The tachinid rearing methods are described at http://janzen.bio.upenn.edu/caterpillars/methodology/how/parasitoid_husbandry.htm. Since its inception, this inventory has reared over 750,000 wild-caught ACG caterpillars. Any frequencies of parasitization reported here need to be considered against this background inventory. Comparative details of the parasitization ecology of these flies will be treated separately in later papers, in the context of the study of all parasitization rates of tachinids on ACG caterpillars, once the overall alpha taxonomy of ACG caterpillar-attacking tachinids is more complete than at present.

### Descriptions and imaging

Species accounts presented in this paper are deliberately brief and only include basic descriptions of body morphology and coloration commonly used in the identification of Tachinidae. The descriptions are complemented with a series of color photos of every species, used to illustrate the morphological differences among them. The morphological terminology used follows Cumming and Wood (2009). All dissections and photography were carried out following the methods detailed in Fleming et al. (2014a). Measurements and examples of parts of the terminalia are illustrated in Fig. 1.

### Voucher specimen management

All caterpillars reared from the ACG efforts receive a unique voucher code in the format yy–SRNP–xxxxx. Any parasitoid emerging from a caterpillar receives the same voucher code as a record of the rearing event. If and when the parasitoid is later dealt with individually it receives a second voucher code unique to it, in the format DHJPARxxxxxxx. These voucher codes assigned to both host and parasitoids may be used to obtain the individual rearing record at http://janzen.bio.upenn.edu/caterpillars/database.lasso.

To date, all DHJPARxxxxxx-coded tachinids have had one leg removed for DNA barcoding at the Biodiversity Institute of Ontario (BIO) in Guelph, ON, Canada. All successful barcodes and collateral data are first deposited in the Barcode of Life Data System (BOLD, www.boldsystems.org) (Ratnasingham and Hebert 2007), and later migrated to GenBank. Each barcoded specimen is also assigned unique accession codes from both the Barcode of Life Data System (BOLD) and GenBank respectively.

Inventoried Tachinidae were collected under Costa Rican government research permits issued to DHJ, and exported from Costa Rica to Philadelphia, en route to their final depository in the Canadian National Insect collection in Ottawa, Canada (CNC). Tachinid identifications for the inventory were done by DHJ in coordination with a) visual inspection by AJF and DMW, b) DNA barcode sequence examination by MAS and DHJ, and c) correlation with host caterpillar identifications by DHJ and WH through the inventory itself. Dates of collection cited for each ACG specimen are the dates of eclosion of the fly, not the date of capture of the caterpillar, since the fly eclosion date is much more representative of the time when that fly species is on the wing than is the time of capture of the host caterpillar. The collector listed on the label is the parataxonomist who found the caterpillar, rather than the person who retrieved the newly eclosed fly from its rearing container. The holotypes of the species newly described herein are all deposited at CNC.

### Acronyms for depositories

AMNH American Museum of Natural History, New York, New York, USA

BMNH The Natural History Museum, London, United Kingdom

CNC Canadian National Collection of Insects, Arachnids and Nematodes, Ottawa, Canada

MNCR Museo Nacional de Costa Rica (formerly Instituto Nacional de Biodiversidad - INBio), Santo Domingo de Heredia, Costa Rica

MNHN Muséum National d'Histoire Naturelle, Paris, France

MZSP Museu de Zoologia da Universidade de São Paulo, São Paulo, Brazil

MRSN Museo Regionale di Scienze Naturali di Torino (collection formerly housed at Museo di Zoologia, Istituto di Zoologia e Anatomia Comparata, Università di Torino - MZUT), Turin, Italy

NMW Naturhistorisches Museum Wien, Vienna, Austria

USNM United States National Museum of Natural History, Washington, D.C., USA

### Interim names of undescribed host species

Names of undescribed host species follow a standardized, interim naming system used for taxonomic units considered as distinct species and identified by DNA barcodes. The interim names are given in the format "*Eois* Janzen52" or "*Caviria regina*DHJ01", where the "species epithet" is either composed of the name of the taxonomist who identified the species and a number or the name of a species-group followed by a code. This prevents confusion with already described species while maintaining traceability of each undescribed species within the ACG project.

### DNA Barcoding

DNA barcodes (DNA sequences from a standardized 5’ region of the mitochondrial cytochrome *c* oxidase I (COI) gene) for all ACG inventoried specimens were obtained using DNA extracts prepared from single legs using a modified glass fibre protocol (Ivanova et al. 2006). A 658-bp region near the 5’ terminus of the COI gene was amplified from the total genomic DNA extract using standard insect primers (LepF1–LepR1 (Hebert et al. 2004)) and following established protocols (Smith et al. 2006, Smith et al. 2008).

## Taxon treatments

### Uramya

Robineau-Desvoidy, 1830

Uramya Robineau-Desvoidy, 1830: 215. Type species: *Uramya producta* Robineau-Desvoidy, 1830, by monotypy.
Olinda Robineau-Desvoidy, 1830: 116. Type species: *Olinda brasiliensis* Robineau-Desvoidy, 1830, by monotypy. **Syn. nov.**
Aporia Macquart, 1846: 297 (1846: 169) (preocc. by Hübner, 1819). Type species: *Aporia quadrimaculata* Macquart, 1846, by monotypy.
Oxydexia Bigot, 1885: xxxiii. Type species: *Oxydexia acuminata* Bigot, 1885, by monotypy.
Neaporia Townsend, 1908: 67 (*nom. nov.* for *Aporia* Macquart but preocc. by Gorham, 1897). Type species: *Aporia quadrimaculata* Macquart, 1846, automatic [by designation of the same species (by monotypy) for *Aporia* Macquart, 1846].
Paraporia Townsend, 1912: 48 (*nom. nov.* for *Neaporia* Townsend). Type species: *Aporia quadrimaculata* Macquart, 1846, automatic [by designation of the same species (automatic) for *Neaporia* Townsend, 1908].
Pseudeuantha Townsend, 1915: 416. Type species: *Pseudeuantha linellii* Townsend, 1915 [= *Lydella indita* Walker, 1860], by original designation.
Uromacquartia Townsend, 1916b: 626. Type species: *Uromacquartia halisidotae* Townsend, 1916, by original designation.
Gymnaporia Townsend, 1919: 170. Type species: *Gymnostylia fasciata* Macquart, 1848, by original designation.
Orthaporia Townsend, 1919: 167. Type species: *Orthaporia similis* Townsend, 1919 [= *Dexia longa* Walker, 1852], by original designation.
Uraporia Townsend, 1919: 170. Type species: *Aporia caudata* Schiner, 1868, by original designation.
Anaporia Townsend, 1919: 560. Type species: *Aporia limacodis* Townsend, 1892, by original designation.
Procleonice Townsend, 1935: 223. Type species: *Procleonice prolixa* Townsend, 1935, by original designation. **Syn. nov.**
Thelairomima Townsend, 1935: 222. Type species: *Thelairomima pictipennis* Townsend, 1935, by original designation.
Uramya **Other Neotropical species included in *Uramya* Robineau-Desvoidy**
acuminata Wulp, 1890: 130 (*Macquartia*). Syntypes: 2 males, 3 females (BMNH) [examined by DMW]. Type localities: Mexico, Guerrero, Amula, 7,000 feet (2 males and 1 female); Omilteme (2 females).
aldrichi Reinhard, 1935: 163 (*Uramya*). Holotype male (USNM) [examined by DMW]. Type locality: Mexico, Oaxaca, Etla. (We have opted to leave this species out of the key on account of the holotype being too damaged; however, it most resembles *U. nitens* Schiner.)
brasiliensis Robineau-Desvoidy, 1830: 116 (*Olinda*). Holotype female (MNHN). Type locality: Brazil, Garatuba [examined by AJF]. **Comb. nov.** (The holotype of this species was presumed lost; after ascertaining its existence, we confirm that it belongs to the genus *Uramya*; unfortunately, it is too damaged to identify properly and as such we have opted to leave this species out of the key.)
brevicauda Curran, 1934: 503 (*Uramya*). Holotype male (AMNH) [examined by DMW]. Type locality: Venezuela, Guanoco.
prolixa Townsend, 1935: 223 (*Procleonice*). Holotype female (BMNH) [examined by DMW]. Type locality: Trinidad. **Syn. nov.**
trinitatis Thompson, 1963: 352 (*Uromacquartia*). Syntypes male and female (USNM) [examined by DMW]. Type locality: Trinidad, Santa Cruz Valley, Mt. Lambert.
caudata Schiner, 1868: 320 (*Aporia*). Syntypes: 2 males, presumed in NMW, but possibly lost. (NMW) Type locality: South America.
fasciata Macquart, 1848: 212 (*Gymnostylia*). Syntypes: 2 males. (BMNH) [examined by DMW]. Type locality: Brazil.
indita Walker, 1861: 306 (*Lydella*). Holotype male [published as female] (BMNH) [examined by DMW]. Type locality: Mexico.
elegans Giglios-Tos, 1863: 3 (*Aporia*). Holotype male (MRSN) [examined by DMW]. Type locality: Mexico, Tuxpango.
linellii Townsend, 1915: 416 (*Pseudeuantha*). Holotype female (USNM) [examined by DMW]. Type locality: Mexico, Tehuantepec.
insolita Guimarães, 1980: 198 (*Uramya*). Holotype male (MZSP) [examined by DMW]. Type locality: Brazil, Santa Catarina, Nova Teutònia.
longa Walker, 1853: 311 (*Dexia*). Holotype male (BMNH) [examined by DMW]. Type locality: South America.
similis Townsend, 1919: 167 (*Orthaporia*). Holotype male (AMNH) [examined by DMW]. Type locality: Brazil, Rio de Janeiro.
nubilis Townsend, 1929: 367 (*Pseudeuantha*). Holotype female (USNM) [examined by DMW]. Type locality: Brazil, São Paulo, Tremembé de Cantareira.
nitens Schiner, 1868: 320 (*Aporia*). Lectotype male (NMW), designated by Guimarães (1971) [examined by DMW]. Type locality: Venezuela.
octomaculata Townsend, 1919: 560 (*Pseudeuantha*). Holotype male (USNM) [examined by DMW]. Type locality: Peru, Huadquina, 5,000 ft.
penai Guimarães, 1980: 200 (*Uramya*). Holotype male (MZSP) [examined by DMW]. Type locality: Bolivia, Cochabamba, Chupare Locotal.
plaumanni Guimarães, 1980: 199 (*Uramya*). Holotype male (MZSP) [examined by DMW]. Type locality: Brazil, Santa Catarina, Nova Teutònia.
producta Robineau-Desvoidy, 1830: 216 (*Uramya*). Neotype male (MZSP), designated by Guimarães (1980) [examined by DMW]. Type locality: Brazil, Rio de Janeiro, Novo Friburgo, Mury.
acuminata Bigot, 1885: xxxiii (*Oxydexia*). Holotype male (BMNH) [examined by DMW]. Type locality: Brazil.
pictipennis Townsend, 1935: 222 (*Thelairomima*). Holotype female (MZSP) [examined by DMW]. Type locality: Brazil, São Paulo, São Vicente.
quadrimaculata Macquart, 1846: 297 (1846: 169) (*Aporia*). Holotype male (MNHN) [examined by DMW]. Type locality: Colombia.
hariola Reinhard, 1961: 206 (*Uramya*). Holotype male (CNC) [examined by DMW]. Type locality: Colombia.
sermyla Walker, 1849: 850 (*Dexia*). Holotype male (BMNH). Type locality: Brazil. (Guimarães (1980) opted to leave this species out of *Uramya* on account of the holotype being too degraded).
setiventris Wulp, 1890: 129 (*Macquartia*). Syntypes: 2 males (BMNH and MRSN) [examined by DMW]. Type localities: Mexico: Orizaba (coll. Bellardi in MRSN); Guerrero, Omilteme, 8,000 ft (BMNH).
sibinivora Guimarães, 1980: 201 (*Uramya*). Holotype male (MZSP) [examined by DMW]. Type locality: Paraguay, Villarica.
townsendi Guimarães, 1980: 200 (*Uramya*). Holotype male (MZSP) [examined by DMW]. Type locality: Brazil, São Paulo, Mogi Guaçu, Fazenda Campininha.
umbratilis Reinhard, 1935: 164 (*Pseudeuantha*). Holotype female (USNM) [examined by DMW]. Type locality: USA, Texas, Donna.
venusta Wulp, 1890: 130 (*Macquartia*). Syntypes: 6 males (BMNH) [examined by DMW]. Type localities: Mexico, Guerrero, Amula, 6,000 ft. (1 male); Xucumanatlan, 7,000 ft. (5 males).
Uramya Uramya producta Robineau-Desvoidy, 1830.

#### Description

**Head**: frontal bristles not extending beyond level of pedicel; eyes haired; facial margin not visible in profile; vibrissa arising at level of facial margin; first flagellomere almost reaching facial margin; arista slightly pubescent; facial ridge devoid of bristles; ocellar bristles weak to absent; inner vertical bristles elongate, parallel, in some cases indistiguishable from upper frontal bristles; outer orbitals weak to absent in males, but strong in females; females with two pairs of proclinate orbital bristles.

**Thorax**: chaetotaxy as follows: postsutural supra-alar bristles 3 (2 in *U. pannosa* **sp. nov.**); acrostichal bristles 2:3; dorsocentral bristles 3:3 (4 in *U. venusta*); postpronotal bristles 4 or 5; two strong scutellar marginal bristles (except 3 in *U. brevicauda*); apical scutellar bristles long and decussate; 2–3 katepisternal bristles; propleuron bare; wings smoky gray, in some species with brown markings or bicolored: yellow at base and brownish-black distally; calypters broad, of yellow-amber color, haired marginally; legs black; claws and pulvilli elongate in males.

**Abdomen**: narrowed and elongate, at least twice as long as wide; frequently acutely produced dorsally into a tail-like process; mid-dorsal depression on ST1+2 reaching hind margin of syntergite; T3, T4 and sometimes ST1+2 with one to three pairs of discal bristles; all tergites with marginals. In females, abdomen usually not as elongate as males and with a rounded posterior end.

#### Diagnosis

*Uramya* is easily distinguished by the following combination of traits: densely haired eyes; facial carina absent; facial ridge bare; parafacial bare (sometimes with a few black setulae in females); ocellar bristles weak to absent; prosternum bare; postpronotum with 4–5 bristles; 3 postsutural supra-alar bristles (2 in *U. pannosa*); 3 large postsutural dorsocentral bristles (4 in *U. venusta*); metathoracic spiracle fringed with plumose hairs of about equal size along anterior and posterior edges, leaving a V-shaped mid-dorsal opening; scutellum with 3–4 pairs of strong marginal scutellar bristles (1 pair of basal scutellar bristles, 1–2 pairs of lateral bristles, and 1 pair of subapical bristles), excluding a pair of strong, crossed apicals; vein M sharply angled at bend; abdomen subcylindrical (in males often with T5 dorsally produced into a tail-like structure), with 1–2 pairs of median discal bristles on tergites 3 and 4.

#### Distribution

Nearctic and Neotropical (not known from Chile and southern Argentina)

#### Ecology

**Figure 2.**
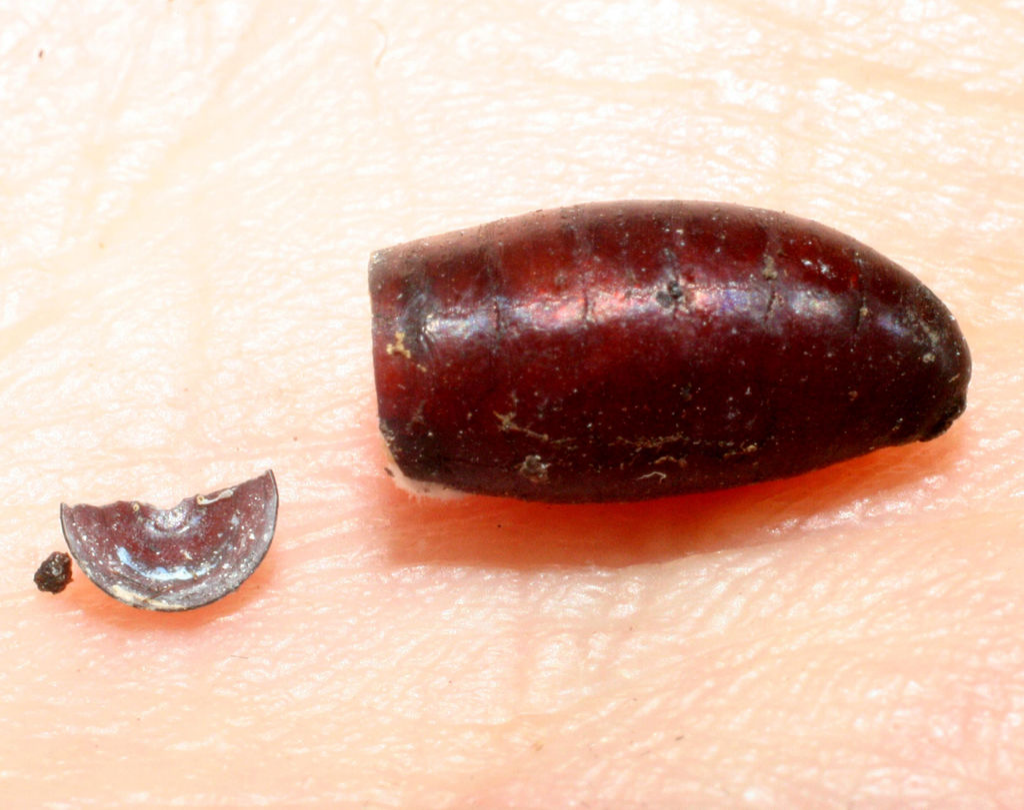
Asymmetrical puparium characteristic ofthe genus *Uramya*, represented here by *Uramya infracta* **sp. nov.** Image voucher in ACG database: 08-SRNP-30125-DHJ462261.jpg.

According to Arnaud (1978) *Uramya* species parasitize lepidopteran larvae in the families Limacodidae, Arctiidae, Megalopygidae and Lasiocampidae. Current data from ACG inventoried larvae confirm this, adding records from the family Dalceridae. *Uramya* puparia are asymmetrical as seen in Fig. 2 (*Uramya infracta* **sp. nov.**).

### Uramya albosetulosa

Fleming & Wood sp. n.

urn:lsid:zoobank.org:act:04777DD1-BAC6-4545-AB51-A64B0804727E

#### Materials

- **Type status:** Holotype. **Occurrence:** occurrenceDetails: http://janzen.sas.upenn.edu; catalogNumber: DHJPAR0018602; recordedBy: D.H. Janzen & W. Hallwachs, Freddy Quesada; individualID: DHJPAR0018602; individualCount: 1; sex: M; lifeStage: adult; preparations: pinned; otherCatalogNumbers: ASTAI1249-07, 99-SRNP-13906; **Taxon:** scientificName: Uramya albosetulosa; phylum: Arthropoda; class: Insecta; order: Diptera; family: Tachinidae; genus: Uramya; specificEpithet: albosetulosa; scientificNameAuthorship: Fleming & Wood; **Location:** continent: Central America; country: Costa Rica; countryCode: CR; stateProvince: Alajuela; county: Area de Conservación Guanacaste; locality: Sector San Cristobal; verbatimLocality: Cementerio Viejo; verbatimElevation: 570; verbatimLatitude: 10.881; verbatimLongitude: -85.389; verbatimCoordinateSystem: Decimal; decimalLatitude: 10.881; decimalLongitude: -85.389; **Identification:** identifiedBy: AJ Fleming; dateIdentified: 2015; **Event:** samplingProtocol: reared from caterpillar of Acharia hyperoche (Limacodidae); verbatimEventDate: Jan-05-2000; **Record Level:** institutionCode: CNC
- **Type status:** Paratype. **Occurrence:** occurrenceDetails: http://janzen.sas.upenn.edu; catalogNumber: DHJPAR0018600; recordedBy: D.H. Janzen & W. Hallwachs, Gloria Sihezar; individualID: DHJPAR0018600; individualCount: 1; sex: F; lifeStage: adult; preparations: pinned; otherCatalogNumbers: ASTAI1247-07, 05-SRNP-3786; **Taxon:** scientificName: Uramya albosetulosa; phylum: Arthropoda; class: Insecta; order: Diptera; family: Tachinidae; genus: Uramya; specificEpithet: albosetulosa; scientificNameAuthorship: Fleming & Wood; **Location:** continent: Central America; country: Costa Rica; countryCode: CR; stateProvince: Alajuela; county: Area de Conservación Guanacaste; locality: Sector San Cristobal; verbatimLocality: Rio Blanco Abajo; verbatimElevation: 500; verbatimLatitude: 10.9; verbatimLongitude: -85.373; verbatimCoordinateSystem: Decimal; decimalLatitude: 10.9; decimalLongitude: -85.373; **Identification:** identifiedBy: AJ Fleming; dateIdentified: 2015; **Event:** samplingProtocol: reared from caterpillar of Acharia hyperoche (Limacodidae); verbatimEventDate: Jul-31-2005; **Record Level:** language: en; institutionCode: CNC; collectionCode: Insects; basisOfRecord: Pinned Specimen
- **Type status:** Paratype. **Occurrence:** occurrenceDetails: http://janzen.sas.upenn.edu; catalogNumber: DHJPAR0018587; recordedBy: D.H. Janzen & W. Hallwachs, Mariano Pereira; individualID: DHJPAR0018587; individualCount: 1; sex: M; lifeStage: adult; preparations: pinned; otherCatalogNumbers: ASTAI1234-07, 04-SRNP-47029; **Taxon:** scientificName: Uramya albosetulosa; phylum: Arthropoda; class: Insecta; order: Diptera; family: Tachinidae; genus: Uramya; specificEpithet: albosetulosa; scientificNameAuthorship: Fleming & Wood; **Location:** continent: Central America; country: Costa Rica; countryCode: CR; stateProvince: Guanacaste; county: Area de Conservación Guanacaste; locality: Sector Cacao; verbatimLocality: Gongora Bananal; verbatimElevation: 600; verbatimLatitude: 10.889; verbatimLongitude: -85.476; verbatimCoordinateSystem: Decimal; decimalLatitude: 10.889; decimalLongitude: -85.476; **Identification:** identifiedBy: AJ Fleming; dateIdentified: 2015; **Event:** samplingProtocol: reared from caterpillar of Acharia sarans (Limacodidae); verbatimEventDate: Jul-28-2004; **Record Level:** institutionCode: CNC
- **Type status:** Paratype. **Occurrence:** occurrenceDetails: http://janzen.sas.upenn.edu; catalogNumber: DHJPAR0029618; recordedBy: D.H. Janzen & W. Hallwachs, Jose Perez; individualID: DHJPAR0029618; individualCount: 1; sex: F; lifeStage: adult; preparations: pinned; otherCatalogNumbers: ASHYM1039-09, 08-SRNP-41429; **Taxon:** scientificName: Uramya albosetulosa; phylum: Arthropoda; class: Insecta; order: Diptera; family: Tachinidae; genus: Uramya; specificEpithet: albosetulosa; scientificNameAuthorship: Fleming & Wood; **Location:** continent: Central America; country: Costa Rica; countryCode: CR; stateProvince: Alajuela; county: Area de Conservación Guanacaste; locality: Sector Rincon Rain Forest; verbatimLocality: Cabanya; verbatimElevation: 340; verbatimLatitude: 10.877; verbatimLongitude: -85.231; verbatimCoordinateSystem: Decimal; decimalLatitude: 10.877; decimalLongitude: -85.231; **Identification:** identifiedBy: AJ Fleming; dateIdentified: 2015; **Event:** samplingProtocol: reared from caterpillar of Acharia hyperoche (Limacodidae); verbatimEventDate: Aug-31-2008; **Record Level:** institutionCode: CNC
- **Type status:** Paratype. **Occurrence:** occurrenceDetails: http://janzen.sas.upenn.edu; catalogNumber: DHJPAR0018583; recordedBy: D.H. Janzen & W. Hallwachs, Esteban Umana; individualID: DHJPAR0018583; individualCount: 1; sex: M; lifeStage: adult; preparations: pinned; otherCatalogNumbers: ASTAI1230-07, 04-SRNP-56376; **Taxon:** scientificName: Uramya albosetulosa; phylum: Arthropoda; class: Insecta; order: Diptera; family: Tachinidae; genus: Uramya; specificEpithet: albosetulosa; scientificNameAuthorship: Fleming & Wood; **Location:** continent: Central America; country: Costa Rica; countryCode: CR; stateProvince: Guanacaste; county: Area de Conservación Guanacaste; locality: Sector Pitilla; verbatimLocality: Pasmompa; verbatimElevation: 440; verbatimLatitude: 11.019; verbatimLongitude: -85.41; verbatimCoordinateSystem: Decimal; decimalLatitude: 11.019; decimalLongitude: -85.41; **Identification:** identifiedBy: AJ Fleming; dateIdentified: 2015; **Event:** samplingProtocol: reared from caterpillar of Acharia apicalis (Limacodidae); verbatimEventDate: Jan-03-2005; **Record Level:** institutionCode: CNC
- **Type status:** Paratype. **Occurrence:** occurrenceDetails: http://janzen.sas.upenn.edu; catalogNumber: DHJPAR0018598; recordedBy: D.H. Janzen & W. Hallwachs, Gloria Sihezar; individualID: DHJPAR0018598; individualCount: 1; sex: F; lifeStage: adult; preparations: pinned; otherCatalogNumbers: ASTAI1245-07, 05-SRNP-3786; **Taxon:** scientificName: Uramya albosetulosa; phylum: Arthropoda; class: Insecta; order: Diptera; family: Tachinidae; genus: Uramya; specificEpithet: albosetulosa; scientificNameAuthorship: Fleming & Wood; **Location:** continent: Central America; country: Costa Rica; countryCode: CR; stateProvince: Alajuela; county: Area de Conservación Guanacaste; locality: Sector San Cristobal; verbatimLocality: Rio Blanco Abajo; verbatimElevation: 500; verbatimLatitude: 10.9; verbatimLongitude: -85.373; verbatimCoordinateSystem: Decimal; decimalLatitude: 10.9; decimalLongitude: -85.373; **Identification:** identifiedBy: AJ Fleming; dateIdentified: 2015; **Event:** samplingProtocol: reared from caterpillar of Acharia hyperoche (Limacodidae); verbatimEventDate: Jul-31-2005; **Record Level:** institutionCode: CNC
- **Type status:** Paratype. **Occurrence:** occurrenceDetails: http://janzen.sas.upenn.edu; catalogNumber: DHJPAR0018601; recordedBy: D.H. Janzen & W. Hallwachs, Esteban Umana; individualID: DHJPAR0018601; individualCount: 1; sex: F; lifeStage: adult; preparations: pinned; otherCatalogNumbers: ASTAI1248-07, 04-SRNP-56377; **Taxon:** scientificName: Uramya albosetulosa; phylum: Arthropoda; class: Insecta; order: Diptera; family: Tachinidae; genus: Uramya; specificEpithet: albosetulosa; scientificNameAuthorship: Fleming & Wood; **Location:** continent: Central America; country: Costa Rica; countryCode: CR; stateProvince: Guanacaste; county: Area de Conservación Guanacaste; locality: Sector Pitilla; verbatimLocality: Pasmompa; verbatimElevation: 440; verbatimLatitude: 11.019; verbatimLongitude: -85.41; verbatimCoordinateSystem: Decimal; decimalLatitude: 11.019; decimalLongitude: -85.41; **Identification:** identifiedBy: AJ Fleming; dateIdentified: 2015; **Event:** samplingProtocol: reared from caterpillar of Acharia apicalis (Limacodidae); verbatimEventDate: Jan-06-2005; **Record Level:** institutionCode: CNC
- **Type status:** Paratype. **Occurrence:** occurrenceDetails: http://janzen.sas.upenn.edu; catalogNumber: DHJPAR0018584; recordedBy: D.H. Janzen & W. Hallwachs, Esteban Umana; individualID: DHJPAR0018584; individualCount: 1; sex: M; lifeStage: adult; preparations: pinned; otherCatalogNumbers: ASTAI1231-07, 04-SRNP-56380; **Taxon:** scientificName: Uramya albosetulosa; phylum: Arthropoda; class: Insecta; order: Diptera; family: Tachinidae; genus: Uramya; specificEpithet: albosetulosa; scientificNameAuthorship: Fleming & Wood; **Location:** continent: Central America; country: Costa Rica; countryCode: CR; stateProvince: Guanacaste; county: Area de Conservación Guanacaste; locality: Sector Pitilla; verbatimLocality: Pasmompa; verbatimElevation: 440; verbatimLatitude: 11.019; verbatimLongitude: -85.41; verbatimCoordinateSystem: Decimal; decimalLatitude: 11.019; decimalLongitude: -85.41; **Identification:** identifiedBy: AJ Fleming; dateIdentified: 2015; **Event:** samplingProtocol: reared from caterpillar of Acharia apicalis (Limacodidae); verbatimEventDate: Jan-04-2005; **Record Level:** institutionCode: CNC
- **Type status:** Paratype. **Occurrence:** occurrenceDetails: http://janzen.sas.upenn.edu; catalogNumber: DHJPAR0020991; recordedBy: D.H. Janzen & W. Hallwachs, Jose Perez; individualID: DHJPAR0020991; individualCount: 1; sex: F; lifeStage: adult; preparations: pinned; otherCatalogNumbers: ASTA1334-07, 07-SRNP-42091; **Taxon:** scientificName: Uramya albosetulosa; phylum: Arthropoda; class: Insecta; order: Diptera; family: Tachinidae; genus: Uramya; specificEpithet: albosetulosa; scientificNameAuthorship: Fleming & Wood; **Location:** continent: Central America; country: Costa Rica; countryCode: CR; stateProvince: Alajuela; county: Area de Conservación Guanacaste; locality: Sector Rincon Rain Forest; verbatimLocality: Camino Rio Francia; verbatimElevation: 410; verbatimLatitude: 10.904; verbatimLongitude: -85.287; verbatimCoordinateSystem: Decimal; decimalLatitude: 10.904; decimalLongitude: -85.287; **Identification:** identifiedBy: AJ Fleming; dateIdentified: 2015; **Event:** samplingProtocol: reared from caterpillar of Acharia hyperoche (Limacodidae); verbatimEventDate: Aug-12-2007; **Record Level:** institutionCode: CNC
- **Type status:** Paratype. **Occurrence:** occurrenceDetails: http://janzen.sas.upenn.edu; catalogNumber: DHJPAR0018585; recordedBy: D.H. Janzen & W. Hallwachs, Esteban Umana; individualID: DHJPAR0018585; individualCount: 1; sex: M; lifeStage: adult; preparations: pinned; otherCatalogNumbers: ASTAI1232-07, 04-SRNP-56379; **Taxon:** scientificName: Uramya albosetulosa; phylum: Arthropoda; class: Insecta; order: Diptera; family: Tachinidae; genus: Uramya; specificEpithet: albosetulosa; scientificNameAuthorship: Fleming & Wood; **Location:** continent: Central America; country: Costa Rica; countryCode: CR; stateProvince: Guanacaste; county: Area de Conservación Guanacaste; locality: Sector Pitilla; verbatimLocality: Pasmompa; verbatimElevation: 440; verbatimLatitude: 11.019; verbatimLongitude: -85.41; verbatimCoordinateSystem: Decimal; decimalLatitude: 11.019; decimalLongitude: -85.41; **Identification:** identifiedBy: AJ Fleming; dateIdentified: 2015; **Event:** samplingProtocol: reared from caterpillar of Acharia apicalis (Limacodidae); verbatimEventDate: Dec-26-2004; **Record Level:** institutionCode: CNC
- **Type status:** Paratype. **Occurrence:** occurrenceDetails: http://janzen.sas.upenn.edu; catalogNumber: DHJPAR0018599; recordedBy: D.H. Janzen & W. Hallwachs, Esteban Umana; individualID: DHJPAR0018599; individualCount: 1; sex: M; lifeStage: adult; preparations: pinned; otherCatalogNumbers: ASTAI1246-07, 04-SRNP-56378; **Taxon:** scientificName: Uramya albosetulosa; phylum: Arthropoda; class: Insecta; order: Diptera; family: Tachinidae; genus: Uramya; specificEpithet: albosetulosa; scientificNameAuthorship: Fleming & Wood; **Location:** continent: Central America; country: Costa Rica; countryCode: CR; stateProvince: Guanacaste; county: Area de Conservación Guanacaste; locality: Sector Pitilla; verbatimLocality: Pasmompa; verbatimElevation: 440; verbatimLatitude: 11.019; verbatimLongitude: -85.41; verbatimCoordinateSystem: Decimal; decimalLatitude: 11.019; decimalLongitude: -85.41; **Identification:** identifiedBy: AJ Fleming; dateIdentified: 2015; **Event:** samplingProtocol: reared from caterpillar of Acharia apicalis (Limacodidae); verbatimEventDate: Dec-26-2004; **Record Level:** institutionCode: CNC

#### Description

**Male** (Fig. 3). Length: 9–14 mm. **Head** (Fig. 3b): antenna: medial surface of first flagellomere dark orange along upper margin, closest to pedicel; pedicel black; arista 1.5X as long as first flagellomere, dark brown and minutely pubescent; palpus dark yellow, haired; fronto-orbital plate, parafacial and gena silver pollinose; gena bearing few fine hairs along lower margin. **Thorax** (Fig. 3a, c): entirely silver pollinose; dorsum of thorax and scutellum with conspicuous yellow-white hairs covering surface; sternopleura, hypopleura, pteropleura and ventral surface of abdomen yellow-white pilose; 2 katepisternal bristles; 3 postsutural supra-alar bristles; postpronotum bearing fine black hairs and anepisternum with fine yellow-white hairs; scutellum bearing 1 pair of discal bristles; underside of scutellum bearing a tuft of white hairs near basal marginal bristle. Legs: reddish-yellow in ground color; femur covered in long yellow hairs interspersed among darker hairs and bristles; tarsus all black. Wings: smoky gray translucent; wing veins not infuscate. **Abdomen** (Fig. 3a): 1 pair of median marginal bristles on ST1+2 and T3; row of marginal bristles on T4 and T5; median discal bristles on T3, T4 and T5; abdomen brown-orange dorsally, with silver pollen on anterior half of T3, T4 and T5; underside of abdomen pale pilose and entirely covered in silver pollinosity. **Terminalia** (Fig. 3d, e, f): sternite 5 consisting of two small lobes; inner margin covered in dense pollinosity appearing darker than surrounding cuticle; apical edges of lobes of sternite 5 bearing many long, stout, outwardly pointing bristles interspersed with longer bristles close to anterior margin; sternite 5 with wide V-shaped median cleft 0.34X the length of the sternite from lobe apex to base; cercus sharply pointed and distinctly tapered; apical section subequal in length to upper lobe of cercus; slightly curved when viewed laterally, with a slight upward hook at its tip; surstylus oblong, curved and scythe-like in lateral view; posterodorsal half haired, with few short apical bristles; tip of surstylus not lobed when viewed dorsally; in dorsal view, surstylus angled inwards and 1.3X as long as cercus.

**Female** (Fig. 4). Length: 7–10 mm. As male, except wing smoky translucent amber color and wing veins slightly infuscate; ground color of abdomen as in male except T5 all black and tergal margins appearing yellow on ventral surface.

#### Diagnosis

*Uramya albosetulosa* can be distinguished from all other Neotropical species of *Uramya* by the following combination of traits: dark brown to black antennae, 3 postsutural supra-alar bristles, underside of scutellum with a tuft of white hairs near basal marginal bristle, 1 pair of median marginal bristles on ST1+2 and T3; T4 and T5 with a row of marginal bristles; underside of abdomen pale pilose and silver pollinose.

#### Etymology

The specific epithet is derived from the Latin adjective “ *albus* ”, for white, and the noun “ *seta* ”, for bristle, in reference to the tuft of white hairs present along the underside of the scutellum in this species.

#### Distribution

Costa Rica, ACG (Guanacaste and Alajuela Provinces), 340–600 m.

#### Ecology

*Uramya albosetulosa* **sp. nov.** has been reared 35 times from *Acharia sarans* (Dyar), *Acharia hyperoche* Dognin and *Acharia apicalis* (Dyar) (Limacodidae), from 2,035 wild-caught mixed siblings and non-siblings found in both dry and rain forest.

### Uramya constricta

Fleming & Wood sp. n.

urn:lsid:zoobank.org:act:5FAF0A60-43C5-4508-A6B1-F93BC0F16586

#### Materials

- **Type status:** Holotype. **Occurrence:** occurrenceDetails: http://janzen.sas.upenn.edu; catalogNumber: DHJPAR0018567; recordedBy: D.H. Janzen & W. Hallwachs, Harry Ramirez; individualID: DHJPAR0018567; individualCount: 1; sex: M; lifeStage: adult; preparations: pinned; otherCatalogNumbers: ASTAI1214-07, 97-SRNP-1486.16; **Taxon:** scientificName: Uramya constricta; phylum: Arthropoda; class: Insecta; order: Diptera; family: Tachinidae; genus: Uramya; specificEpithet: constricta; scientificNameAuthorship: Fleming & Wood; **Location:** continent: Central America; country: Costa Rica; countryCode: CR; stateProvince: Guanacaste; county: Area de Conservación Guanacaste; locality: Sector Cacao; verbatimLocality: Estacion Cacao; verbatimElevation: 1150; verbatimLatitude: 10.927; verbatimLongitude: -85.468; verbatimCoordinateSystem: Decimal; decimalLatitude: 10.927; decimalLongitude: -85.468; **Identification:** identifiedBy: AJ Fleming; dateIdentified: 2015; **Event:** samplingProtocol: reared from caterpillar of *Acharia ophelians* (Limacodidae); verbatimEventDate: 10-Aug-1997; **Record Level:** language: en; institutionCode: CNC; collectionCode: Insects; basisOfRecord: Pinned Specimen
- **Type status:** Paratype. **Occurrence:** occurrenceDetails: http://janzen.sas.upenn.edu; catalogNumber: DHJPAR0018569; recordedBy: D.H. Janzen & W. Hallwachs, Harry Ramirez; individualID: DHJPAR0018569; individualCount: 1; sex: M; lifeStage: adult; preparations: pinned; otherCatalogNumbers: ASTAI1216-07, 97-SRNP-1486; **Taxon:** scientificName: Uramya constricta; phylum: Arthropoda; class: Insecta; order: Diptera; family: Tachinidae; genus: Uramya; specificEpithet: constricta; scientificNameAuthorship: Fleming & Wood; **Location:** continent: Central America; country: Costa Rica; countryCode: CR; stateProvince: Guanacaste; county: Area de Conservación Guanacaste; locality: Sector Cacao; verbatimLocality: Estacion Cacao; verbatimElevation: 1150; verbatimLatitude: 10.927; verbatimLongitude: -85.468; verbatimCoordinateSystem: Decimal; decimalLatitude: 10.927; decimalLongitude: -85.468; **Identification:** identifiedBy: AJ Fleming; dateIdentified: 2015; **Event:** samplingProtocol: reared from caterpillar of *Acharia ophelians* (Limacodidae); verbatimEventDate: 02-Aug-1997; **Record Level:** language: en; institutionCode: CNC; collectionCode: Insects; basisOfRecord: Pinned Specimen
- **Type status:** Paratype. **Occurrence:** occurrenceDetails: http://janzen.sas.upenn.edu; catalogNumber: DHJPAR0018559; recordedBy: D.H. Janzen & W. Hallwachs, Harry Ramirez; individualID: DHJPAR0018559; individualCount: 1; sex: F; lifeStage: adult; preparations: pinned; otherCatalogNumbers: ASTAI1206-07, 97-SRNP-1486.10; **Taxon:** scientificName: Uramya constricta; phylum: Arthropoda; class: Insecta; order: Diptera; family: Tachinidae; genus: Uramya; specificEpithet: constricta; scientificNameAuthorship: Fleming & Wood; **Location:** continent: Central America; country: Costa Rica; countryCode: CR; stateProvince: Guanacaste; county: Area de Conservación Guanacaste; locality: Sector Cacao; verbatimLocality: Estacion Cacao; verbatimElevation: 1150; verbatimLatitude: 10.927; verbatimLongitude: -85.468; verbatimCoordinateSystem: Decimal; decimalLatitude: 10.927; decimalLongitude: -85.468; **Identification:** identifiedBy: AJ Fleming; dateIdentified: 2015; **Event:** samplingProtocol: reared from caterpillar of *Acharia ophelians* (Limacodidae); verbatimEventDate: 10-Aug-1997; **Record Level:** language: en; institutionCode: CNC; collectionCode: Insects; basisOfRecord: Pinned Specimen
- **Type status:** Paratype. **Occurrence:** occurrenceDetails: http://janzen.sas.upenn.edu; catalogNumber: DHJPAR0018564; recordedBy: D.H. Janzen & W. Hallwachs, Roster Moraga; individualID: DHJPAR0018564; individualCount: 1; sex: M; lifeStage: adult; preparations: pinned; otherCatalogNumbers: ASTAI1211-07, 97-SRNP-1542; **Taxon:** scientificName: Uramya constricta; phylum: Arthropoda; class: Insecta; order: Diptera; family: Tachinidae; genus: Uramya; specificEpithet: constricta; scientificNameAuthorship: Fleming & Wood; **Location:** continent: Central America; country: Costa Rica; countryCode: CR; stateProvince: Guanacaste; county: Area de Conservación Guanacaste; locality: Sector Cacao; verbatimLocality: Estacion Cacao; verbatimElevation: 1150; verbatimLatitude: 10.927; verbatimLongitude: -85.468; verbatimCoordinateSystem: Decimal; decimalLatitude: 10.927; decimalLongitude: -85.468; **Identification:** identifiedBy: AJ Fleming; dateIdentified: 2015; **Event:** samplingProtocol: reared from caterpillar of *Euclea mesoamericana*DHJ04 (Limacodidae); verbatimEventDate: 14-Aug-1997; **Record Level:** language: en; institutionCode: CNC; collectionCode: Insects; basisOfRecord: Pinned Specimen
- **Type status:** Paratype. **Occurrence:** occurrenceDetails: http://janzen.sas.upenn.edu; catalogNumber: DHJPAR0018560; recordedBy: D.H. Janzen & W. Hallwachs, Harry Ramirez; individualID: DHJPAR0018560; individualCount: 1; sex: F; lifeStage: adult; preparations: pinned; otherCatalogNumbers: ASTAI1207-07, 97-SRNP-1486.60; **Taxon:** scientificName: Uramya constricta; phylum: Arthropoda; class: Insecta; order: Diptera; family: Tachinidae; genus: Uramya; specificEpithet: constricta; scientificNameAuthorship: Fleming & Wood; **Location:** continent: Central America; country: Costa Rica; countryCode: CR; stateProvince: Guanacaste; county: Area de Conservación Guanacaste; locality: Sector Cacao; verbatimLocality: Estacion Cacao; verbatimElevation: 1150; verbatimLatitude: 10.927; verbatimLongitude: -85.468; verbatimCoordinateSystem: Decimal; decimalLatitude: 10.927; decimalLongitude: -85.468; **Identification:** identifiedBy: AJ Fleming; dateIdentified: 2015; **Event:** samplingProtocol: reared from caterpillar of *Acharia ophelians* (Limacodidae); verbatimEventDate: 02-Aug-1997; **Record Level:** language: en; institutionCode: CNC; collectionCode: Insects; basisOfRecord: Pinned Specimen
- **Type status:** Paratype. **Occurrence:** occurrenceDetails: http://janzen.sas.upenn.edu; catalogNumber: DHJPAR0018565; recordedBy: D.H. Janzen & W. Hallwachs, Harry Ramirez; individualID: DHJPAR0018565; individualCount: 1; sex: M; lifeStage: adult; preparations: pinned; otherCatalogNumbers: ASTAI1212-07, 97-SRNP-1486.11; **Taxon:** scientificName: Uramya constricta; phylum: Arthropoda; class: Insecta; order: Diptera; family: Tachinidae; genus: Uramya; specificEpithet: constricta; scientificNameAuthorship: Fleming & Wood; **Location:** continent: Central America; country: Costa Rica; countryCode: CR; stateProvince: Guanacaste; county: Area de Conservación Guanacaste; locality: Sector Cacao; verbatimLocality: Estacion Cacao; verbatimElevation: 1150; verbatimLatitude: 10.927; verbatimLongitude: -85.468; verbatimCoordinateSystem: Decimal; decimalLatitude: 10.927; decimalLongitude: -85.468; **Identification:** identifiedBy: AJ Fleming; dateIdentified: 2015; **Event:** samplingProtocol: reared from caterpillar of *Acharia ophelians* (Limacodidae); verbatimEventDate: 02-Aug-1997; **Record Level:** language: en; institutionCode: CNC; collectionCode: Insects; basisOfRecord: Pinned Specimen
- **Type status:** Paratype. **Occurrence:** occurrenceDetails: http://janzen.sas.upenn.edu; catalogNumber: DHJPAR0018563; recordedBy: D.H. Janzen & W. Hallwachs, Harry Ramirez; individualID: DHJPAR0018563; individualCount: 1; sex: M; lifeStage: adult; preparations: pinned; otherCatalogNumbers: ASTAI1210-07, 97-SRNP-1486.61; **Taxon:** scientificName: Uramya constricta; phylum: Arthropoda; class: Insecta; order: Diptera; family: Tachinidae; genus: Uramya; specificEpithet: constricta; scientificNameAuthorship: Fleming & Wood; **Location:** continent: Central America; country: Costa Rica; countryCode: CR; stateProvince: Guanacaste; county: Area de Conservación Guanacaste; locality: Sector Cacao; verbatimLocality: Estacion Cacao; verbatimElevation: 1150; verbatimLatitude: 10.927; verbatimLongitude: -85.468; verbatimCoordinateSystem: Decimal; decimalLatitude: 10.927; decimalLongitude: -85.468; **Identification:** identifiedBy: AJ Fleming; dateIdentified: 2015; **Event:** samplingProtocol: reared from caterpillar of *Acharia ophelians* (Limacodidae); verbatimEventDate: 04-Aug-1997; **Record Level:** language: en; institutionCode: CNC; collectionCode: Insects; basisOfRecord: Pinned Specimen
- **Type status:** Paratype. **Occurrence:** occurrenceDetails: http://janzen.sas.upenn.edu; catalogNumber: DHJPAR0018566; recordedBy: D.H. Janzen & W. Hallwachs, Harry Ramirez; individualID: DHJPAR0018566; individualCount: 1; sex: F; lifeStage: adult; preparations: pinned; otherCatalogNumbers: ASTAI1213-07, 97-SRNP-1486.48; **Taxon:** scientificName: Uramya constricta; phylum: Arthropoda; class: Insecta; order: Diptera; family: Tachinidae; genus: Uramya; specificEpithet: constricta; scientificNameAuthorship: Fleming & Wood; **Location:** continent: Central America; country: Costa Rica; countryCode: CR; stateProvince: Guanacaste; county: Area de Conservación Guanacaste; locality: Sector Cacao; verbatimLocality: Estacion Cacao; verbatimElevation: 1150; verbatimLatitude: 10.927; verbatimLongitude: -85.468; verbatimCoordinateSystem: Decimal; decimalLatitude: 10.927; decimalLongitude: -85.468; **Identification:** identifiedBy: AJ Fleming; dateIdentified: 2015; **Event:** samplingProtocol: reared from caterpillar of *Acharia ophelians* (Limacodidae); verbatimEventDate: 02-Aug-1997; **Record Level:** language: en; institutionCode: CNC; collectionCode: Insects; basisOfRecord: Pinned Specimen
- **Type status:** Paratype. **Occurrence:** occurrenceDetails: http://janzen.sas.upenn.edu; catalogNumber: DHJPAR0018562; recordedBy: D.H. Janzen & W. Hallwachs, Harry Ramirez; individualID: DHJPAR0018562; individualCount: 1; sex: F; lifeStage: adult; preparations: pinned; otherCatalogNumbers: ASTAI1209-07, 97-SRNP-1486.30; **Taxon:** scientificName: Uramya constricta; phylum: Arthropoda; class: Insecta; order: Diptera; family: Tachinidae; genus: Uramya; specificEpithet: constricta; scientificNameAuthorship: Fleming & Wood; **Location:** continent: Central America; country: Costa Rica; countryCode: CR; stateProvince: Guanacaste; county: Area de Conservación Guanacaste; locality: Sector Cacao; verbatimLocality: Estacion Cacao; verbatimElevation: 1150; verbatimLatitude: 10.927; verbatimLongitude: -85.468; verbatimCoordinateSystem: Decimal; decimalLatitude: 10.927; decimalLongitude: -85.468; **Identification:** identifiedBy: AJ Fleming; dateIdentified: 2015; **Event:** samplingProtocol: reared from caterpillar of *Acharia ophelians* (Limacodidae); verbatimEventDate: 02-Aug-1997; **Record Level:** language: en; institutionCode: CNC; collectionCode: Insects; basisOfRecord: Pinned Specimen
- **Type status:** Paratype. **Occurrence:** occurrenceDetails: http://janzen.sas.upenn.edu; catalogNumber: DHJPAR0018570; recordedBy: D.H. Janzen & W. Hallwachs, Harry Ramirez; individualID: DHJPAR0018570; individualCount: 1; sex: F; lifeStage: adult; preparations: pinned; otherCatalogNumbers: ASTAI1217-07, 97-SRNP-1486.35; **Taxon:** scientificName: Uramya constricta; phylum: Arthropoda; class: Insecta; order: Diptera; family: Tachinidae; genus: Uramya; specificEpithet: constricta; scientificNameAuthorship: Fleming & Wood; **Location:** continent: Central America; country: Costa Rica; countryCode: CR; stateProvince: Guanacaste; county: Area de Conservación Guanacaste; locality: Sector Cacao; verbatimLocality: Estacion Cacao; verbatimElevation: 1150; verbatimLatitude: 10.927; verbatimLongitude: -85.468; verbatimCoordinateSystem: Decimal; decimalLatitude: 10.927; decimalLongitude: -85.468; **Identification:** identifiedBy: AJ Fleming; dateIdentified: 2015; **Event:** samplingProtocol: reared from caterpillar of *Acharia ophelians* (Limacodidae); verbatimEventDate: 02-Aug-1997; **Record Level:** language: en; institutionCode: CNC; collectionCode: Insects; basisOfRecord: Pinned Specimen
- **Type status:** Paratype. **Occurrence:** occurrenceDetails: http://janzen.sas.upenn.edu; catalogNumber: DHJPAR0018561; recordedBy: D.H. Janzen & W. Hallwachs, Harry Ramirez; individualID: DHJPAR0018561; individualCount: 1; sex: F; lifeStage: adult; preparations: pinned; otherCatalogNumbers: ASTAI1208-07, 97-SRNP-1486.21; **Taxon:** scientificName: Uramya constricta; phylum: Arthropoda; class: Insecta; order: Diptera; family: Tachinidae; genus: Uramya; specificEpithet: constricta; scientificNameAuthorship: Fleming & Wood; **Location:** continent: Central America; country: Costa Rica; countryCode: CR; stateProvince: Guanacaste; county: Area de Conservación Guanacaste; locality: Sector Cacao; verbatimLocality: Estacion Cacao; verbatimElevation: 1150; verbatimLatitude: 10.927; verbatimLongitude: -85.468; verbatimCoordinateSystem: Decimal; decimalLatitude: 10.927; decimalLongitude: -85.468; **Identification:** identifiedBy: AJ Fleming; dateIdentified: 2015; **Event:** samplingProtocol: reared from caterpillar of *Acharia ophelians* (Limacodidae); **Record Level:** language: en; institutionCode: CNC; collectionCode: Insects; basisOfRecord: Pinned Specimen
- **Type status:** Paratype. **Occurrence:** occurrenceDetails: http://janzen.sas.upenn.edu; catalogNumber: DHJPAR0018568; recordedBy: D.H. Janzen & W. Hallwachs, Harry Ramirez; individualID: DHJPAR0018568; individualCount: 1; sex: M; lifeStage: adult; preparations: pinned; otherCatalogNumbers: ASTAI1215-07, 97-SRNP-1486.41; **Taxon:** scientificName: Uramya constricta; phylum: Arthropoda; class: Insecta; order: Diptera; family: Tachinidae; genus: Uramya; specificEpithet: constricta; scientificNameAuthorship: Fleming & Wood; **Location:** continent: Central America; country: Costa Rica; countryCode: CR; stateProvince: Guanacaste; county: Area de Conservación Guanacaste; locality: Sector Cacao; verbatimLocality: Estacion Cacao; verbatimElevation: 1150; verbatimLatitude: 10.927; verbatimLongitude: -85.468; verbatimCoordinateSystem: Decimal; decimalLatitude: 10.927; decimalLongitude: -85.468; **Identification:** identifiedBy: AJ Fleming; dateIdentified: 2015; **Event:** samplingProtocol: reared from caterpillar of *Acharia ophelians* (Limacodidae); verbatimEventDate: 13-Aug-1997; **Record Level:** language: en; institutionCode: CNC; collectionCode: Insects; basisOfRecord: Pinned Specimen

#### Description

**Male** (Fig. 5). Length: 11–15 mm. **Head** (Fig. 5b): antenna: first flagellomere dark brown on lower 2/3, turning to bright orange on upper third, adjacent to pedicel; pedicel dark black, with orange undertones; arista 1.5X as long as first flagellomere, dark brown and minutely pubescent; palpus dark orange and haired; fronto-orbital plate, parafacial and gena silver pollinose; gena with a cluster of hairs along lower 1/3 to 1/2. **Thorax** (Fig. 5a, c): entirely gray pollinose; dorsum of thorax and scutellum black pilose; sternopleura, hypopleura, pteropleura, and ventral surface of abdomen white pilose; 2 katepisternal bristles; 3 postsutural supra-alar bristles; postpronotum and anepisternum bearing fine black hairs; underside of scutellum bearing a tuft of black hairs near basal marginal bristle. Legs: fore femur gray pollinose, densely covered in thin dark hairs; mid and hind femora dark brown to black in ground color, contrasting with the dark orange tibiae. Wing: smoky grey transparent; wing veins not infuscate. **Abdomen** (Fig. 5a): median marginal bristles present on all abdominal tergites (ST1+2, T3, T4 and T5); 1 pair of median discal bristles on T3, T4 and T5; ground color of abdomen brown-black up to tergite 5; silver pollinosity present on anterior half of T3, T4 and T5; ST1+2 with lateral gray pollinose spots on posterior half; base of ST1+2 slightly constricted, giving the abdomen a basaly tapered appearance. **Terminalia** (Fig. 5d, e, f): sternite 5 with two small lobes, inner margin covered in dense pollinosity, appearing darker than surrounding cuticle; apical edges of lobes of sternite 5 bearing short, stout bristles interspersed with longer bristles close to apical margin; sternite 5 with wide V-shaped median cleft, 0.4X the length of sternite from lobe apex to base; cercus sharply pointed, distinctly tapered; apical section 1.4X as long as upper lobe; strongly curved when viewed laterally and with a slight upward hook at its tip; surstylus equilaterally oblong and scythe-like in lateral view; posterodorsal half densely haired; apex of surstylus with a slight lobe when viewed dorsally and few short apical bristles; surstyli weakly angled inwards in dorsal view, almost parallel; surstylus 1.6X as long as cercus.

**Female** (Fig. 6). Length: 7–10 mm. As male, except arista 1.7X as long as first flagellomere, and 3 katepisternal bristles.

#### Diagnosis

*Uramya constricta* can be distinguished from all other Neotropical species of *Uramya* by the following combination of traits: pedicel dark brown, 3 postsutural supra-alar bristles, underside of scutellum with a tuft of black hairs near basal marginal bristle, only 1 pair of median marginal bristles on ST1+2, T3 and T4, and by the shape of the surstylus.

#### Etymology

The specific epithet is derived from the Latin adjective “*constrictus*”, for constricted or compressed, in reference to the slightly constricted base of ST1+2 where it meets the thorax.

#### Distribution

Costa Rica, ACG (Prov. Guanacaste), 1,150 m.

#### Ecology

*Uramya constricta* has been reared 38 times from *Acharia ophelians* Dyar (Limacodidae) (one sibling brood of caterpillars) and once from *Euclea mesoamericana*DHJ04 (Lepidoptera: Limacodidae) in ACG cloud forest.

### Uramya contraria

Fleming & Wood sp. n.

urn:lsid:zoobank.org:act:A95CABAB-81A0-4C60-BA89-8A457B1E773E

#### Materials

- **Type status:** Holotype. **Occurrence:** occurrenceDetails: http://janzen.sas.upenn.edu; catalogNumber: DHJPAR0022003; recordedBy: D.H. Janzen & W. Hallwachs, Calixto Moraga, Petrona Rios, Manuel Rios; individualID: DHJPAR0022003; individualCount: 1; sex: M; lifeStage: adult; preparations: pinned; otherCatalogNumbers: ASTAT1141-07, 07-SRNP-32916; **Taxon:** scientificName: Uramya contraria; phylum: Arthropoda; class: Insecta; order: Diptera; family: Tachinidae; genus: Uramya; specificEpithet: contraria; scientificNameAuthorship: Fleming & Wood; **Location:** continent: Central America; country: Costa Rica; countryCode: CR; stateProvince: Guanacaste; county: Area de Conservación Guanacaste; locality: Sector Pitilla; verbatimLocality: Sendero Rotulo; verbatimElevation: 510; verbatimLatitude: 11.014; verbatimLongitude: -85.424; verbatimCoordinateSystem: Decimal; decimalLatitude: 11.014; decimalLongitude: -85.424; **Identification:** identifiedBy: AJ Fleming; dateIdentified: 2015; **Event:** samplingProtocol: reared from caterpillar of *Caviria regina*DHJ04 (Erebidae); verbatimEventDate: 01-Sep-2007; **Record Level:** language: en; institutionCode: CNC; collectionCode: Insects; basisOfRecord: Pinned Specimen
- **Type status:** Paratype. **Occurrence:** occurrenceDetails: http://janzen.sas.upenn.edu; catalogNumber: DHJPAR0021038; recordedBy: D.H. Janzen & W. Hallwachs, Calixto Moraga, Petrona Rios, Manuel Rios; individualID: DHJPAR0021038; individualCount: 1; sex: F; lifeStage: adult; preparations: pinned; otherCatalogNumbers: ASTA1381-07, 07-SRNP-32877; **Taxon:** scientificName: Uramya contraria; phylum: Arthropoda; class: Insecta; order: Diptera; family: Tachinidae; genus: Uramya; specificEpithet: contraria; scientificNameAuthorship: Fleming & Wood; **Location:** continent: Central America; country: Costa Rica; countryCode: CR; stateProvince: Guanacaste; county: Area de Conservación Guanacaste; locality: Sector Pitilla; verbatimLocality: Casa Roberto; verbatimElevation: 520; verbatimLatitude: 11.011; verbatimLongitude: -85.421; verbatimCoordinateSystem: Decimal; decimalLatitude: 11.011; decimalLongitude: -85.421; **Identification:** identifiedBy: AJ Fleming; dateIdentified: 2015; **Event:** samplingProtocol: reared from caterpillar of *Caviria regina*DHJ04 (Erebidae); verbatimEventDate: 26-Aug-2007; **Record Level:** language: en; institutionCode: CNC; collectionCode: Insects; basisOfRecord: Pinned Specimen
- **Type status:** Paratype. **Occurrence:** occurrenceDetails: http://janzen.sas.upenn.edu; catalogNumber: DHJPAR0021039; recordedBy: D.H. Janzen & W. Hallwachs, Calixto Moraga, Petrona Rios, Manuel Rios; individualID: DHJPAR0021039; individualCount: 1; sex: F; lifeStage: adult; preparations: pinned; otherCatalogNumbers: ASTA1382-07, 07-SRNP-32922; **Taxon:** scientificName: Uramya contraria; phylum: Arthropoda; class: Insecta; order: Diptera; family: Tachinidae; genus: Uramya; specificEpithet: contraria; scientificNameAuthorship: Fleming & Wood; **Location:** continent: Central America; country: Costa Rica; countryCode: CR; stateProvince: Guanacaste; county: Area de Conservación Guanacaste; locality: Sector Pitilla; verbatimLocality: Casa Roberto; verbatimElevation: 520; verbatimLatitude: 11.011; verbatimLongitude: -85.421; verbatimCoordinateSystem: Decimal; decimalLatitude: 11.011; decimalLongitude: -85.421; **Identification:** identifiedBy: AJ Fleming; dateIdentified: 2015; **Event:** samplingProtocol: reared from caterpillar of *Caviria regina*DHJ04 (Erebidae); verbatimEventDate: 31-Aug-2007; **Record Level:** language: en; institutionCode: CNC; collectionCode: Insects; basisOfRecord: Pinned Specimen
- **Type status:** Paratype. **Occurrence:** occurrenceDetails: http://janzen.sas.upenn.edu; catalogNumber: DHJPAR0022005; recordedBy: D.H. Janzen & W. Hallwachs, Calixto Moraga, Petrona Rios, Manuel Rios; individualID: DHJPAR0022005; individualCount: 1; sex: F; lifeStage: adult; preparations: pinned; otherCatalogNumbers: ASTAT1143-07, 07-SRNP-33078; **Taxon:** scientificName: Uramya contraria; phylum: Arthropoda; class: Insecta; order: Diptera; family: Tachinidae; genus: Uramya; specificEpithet: contraria; scientificNameAuthorship: Fleming & Wood; **Location:** continent: Central America; country: Costa Rica; countryCode: CR; stateProvince: Guanacaste; county: Area de Conservación Guanacaste; locality: Sector Pitilla; verbatimLocality: Pasmompa; verbatimElevation: 440; verbatimLatitude: 11.019; verbatimLongitude: -85.41; verbatimCoordinateSystem: Decimal; decimalLatitude: 11.019; decimalLongitude: -85.41; **Identification:** identifiedBy: AJ Fleming; dateIdentified: 2015; **Event:** samplingProtocol: reared from caterpillar of *Caviria regina*DHJ04 (Erebidae); verbatimEventDate: 13-Sep-2007; **Record Level:** language: en; institutionCode: CNC; collectionCode: Insects; basisOfRecord: Pinned Specimen

#### Description

**Male** (Fig. 7). Length: 12­–13 mm. **Head** (Fig. 7b): antenna black, with orange medially and at base, adjacent to pedicel; pedicel black; arista 1.5X as long as first flagellomere, dark brown and minutely pubescent; palpus dark yellow and haired; fronto-orbital plate, parafacial and gena silver pollinose; gena bearing fine hairs along lower margin. **Thorax** (Fig. 7b, c): entirely gray pollinose; dorsum of thorax and scutellum covered by conspicuous yellow-white hairs; sternopleura, hypopleura, pteropleura, and ventral surface of abdomen yellow-white pilose; 2 katepisternal bristles; 3 postsutural supra-alar bristles, 2nd postsutural supra-alar 4X as long as first; postpronotum bearing fine black hairs and anepisternum with fine yellow-white hairs; scutellum bearing 1 pair of discal bristles; underside of scutellum bearing a tuft of white hairs near basal marginal bristle. Legs: reddish-yellow in ground color; femora covered in long, yellow hairs interspersed among darker hairs and bristles; tarsi all black. Wing: smoky gray translucent; wing veins strongly infuscate; infuscation of wing veins becoming more generalized and blending together to a dark gray tone along basal portion of the wing. **Abdomen** (Fig. 7a): 1 pair of median marginal bristles on ST1+2 and T3; row of marginal bristles on T4 and T5; 2 pairs of median discal bristles on T3 and 1 pair on T4; abdomen dark brown dorsally, with silver pollen on anterior half of T3, T4 and T5; ST1+2 with 2 pollinose spots on either side of mid-dorsal depression; underside of abdomen entirely covered in silver pollinosity. **Terminalia** (Fig. 7d, e, f): sternite 5 with two small lobes; inner margin covered in dense pollinosity, appearing darker than surrounding cuticle; apical edges of lobes of sternite 5 bearing many long, stout, outwardly pointed bristles interspersed with longer bristles close to lobe margin; sternite 5 with wide V-shaped median cleft, 0.32X length of sternite from lobe apex to base; cercus sharply pointed and distinctly tapered; apical section 1.7X length of upper lobe; slightly curved when viewed laterally, with a slight upward hook at its tip; surstylus narrow, curved and scythe-like in lateral view; middle third of surstylus haired, otherwise almost bare; tip of surstylus not lobed when viewed dorsally and distinctly inwardly angled; surstylus 0.9X as long as cercus.

**Female** (Fig. 8). Length: 8–11 mm. As male, except with a slight golden tinge around the thoracic suture, one pair of median discal bristles on T3, T4 and T5, wing smoky brown translucent, only slightly infuscate around major wing veins.

#### Diagnosis

*Uramya contraria* can be distinguished from all other Neotropical species of *Uramya* by the following combination of traits: dark brown to black antennae, 3 postsutural supra-alar bristles, 2 strong lateral scutellar bristles, no discal scutellar bristles, underside of scutellum with a tuft of white hairs near basal marginal bristle, abdomen flattened dorsoventrally, ST1+2 with lateral white pollinose spots on either side of mid-dorsal depression, T5 subtriangular, not strongly produced into a long, tail-like process, 1 pair of median discal bristles on T3, T4 and T5, and silver pollinosity on underside of abdomen.

#### Etymology

The species epithet is derived from the latin adjective " *contrarius* " for contrary or opposed, referring to its overall similarity but differing terminalia to *U. halisidotae*.

#### Distribution

Costa Rica, ACG (Prov. Guanacaste), 440–520 m.

#### Ecology

*Uramya contraria* has been reared four times from a sample of 100 wild-caught, non-sibling *Caviria regina* Cramer (Erebidae, Lymantriinae) in ACG rain forest.

### Uramya infracta

Fleming & Wood sp. n.

urn:lsid:zoobank.org:act:F7921F5C-3BC1-4F80-9900-362C8B1D8892

#### Materials

- **Type status:** Holotype. **Occurrence:** occurrenceDetails: http://janzen.sas.upenn.edu; catalogNumber: DHJPAR0024628; recordedBy: D.H. Janzen & W. Hallwachs, Calixto Moraga; individualID: DHJPAR0024628; individualCount: 1; sex: M; lifeStage: adult; preparations: pinned; otherCatalogNumbers: ASTAW738-08, 08-SRNP-30125; **Taxon:** scientificName: Uramya infracta; phylum: Arthropoda; class: Insecta; order: Diptera; family: Tachinidae; genus: Uramya; specificEpithet: infracta; scientificNameAuthorship: Fleming & Wood; **Location:** continent: Central America; country: Costa Rica; countryCode: CR; stateProvince: Guanacaste; county: Area de Conservación Guanacaste; locality: Sector Pitilla; verbatimLocality: Sendero Orosilito; verbatimElevation: 900; verbatimLatitude: 10.983; verbatimLongitude: -85.436; verbatimCoordinateSystem: Decimal; decimalLatitude: 10.983; decimalLongitude: -85.436; **Identification:** identifiedBy: AJ Fleming; dateIdentified: 2015; **Event:** samplingProtocol: reared from caterpillar of *Natada fusca* (Limacodidae); verbatimEventDate: Mar-17-2008; **Record Level:** language: en; institutionCode: CNC; collectionCode: Insects; basisOfRecord: Pinned Specimen

#### Description

**Male** (Fig. 9). Length: 14 mm. **Head** (Fig. 9b): antenna: medial surface of first flagellomere with a dark orange tinge along upper margin, closest to pedicel; pedicel black to dark brown; arista 1.5X as long as first flagellomere, dark brown and minutely pubescent; palpus dark yellow and haired; fronto-orbital plate, parafacial and gena silver pollinose; gena with few fine hairs along lower margin; frontogenal suture darkened but not black. **Thorax** (Fig. 9a, c): entirely gray pollinose; dorsum of thorax and scutellum with conspicuous black hairs covering surface; sternopleura, hypopleura, pteropleura and ventral surface of abdomen yellow-white pilose; 3 katepisternal bristles; 3 postsutural supra-alar bristles, 2nd postsutural supra-alar 3X as long as first; postpronotum and anepisternum bearing fine black hairs; scutellum bearing 1 pair of almost indistinct discal bristles, and 3 strong lateral marginal bristles; underside of scutellum bearing a tuft of black hairs near basal marginal bristle. Legs: black in ground color, femora covered in long yellow hairs interspersed around femoral bristles; tibiae of dark yellow ground color; tarsi all black. Wing: smoky clear translucent; wing veins slightly infuscate. **Abdomen** (Fig. 9a): 1 pair of median marginal bristles on ST1+2; row of marginal bristles on T3 and T4; median discal bristles on T3 and T4; black ground color, with silver pollen on either side of mid-dorsal depression on ST1+2; silver pollen on anterior half of T3 and T4; T5 with 2 silver pollinose spots on either side; silver pollinosity extending to underside of abdomen on T3 and T4. **Terminalia** (Fig. 9d, e, f): sternite 5 with two small lobes, inner margin covered in dense pollinosity appearing slightly darker than surrounding cuticle; apical edges of lobes of sternite 5 bearing many long, stout, outwardly pointed bristles interspersed among shorter hairs, with longer bristles closest to lobe margin; sternite 5 with wide Y-shaped median cleft, 0.48X length of sternite from lobe apex to base; cercus sharply pointed, distinctly tapered; apical section 1.6X length of upper lobe; strongly curved downwards when viewed laterally, lacking any upward hook at its tip; surstylus narrow, curved downwards, and scythe-like in lateral view; surstylus haired along almost its entire length, with tip not lobed when viewed dorsally; in dorsal view, surstyli angled medially so that tips are almost pointing inwards; surstylus 1.3X as long as cercus.

**Female**: Unknown.

#### Diagnosis

*Uramya infracta* can be distinguished from all other Neotropical species of *Uramya* by the following combination of traits: dark brown to black antennae with only a slight orange tinge and dark brown pedicel, thorax with black hairs interspersed among the bristles dorsally, and yellow-white hairs on sides and ventrally, underside of scutellum with a tuft of black hairs near basal marginal bristle, 1 pair of median marginal bristles on ST1+2, and a row of marginal bristles on T3 and T4.

#### Etymology

The species epithet is derived from the latin adjective " *infractus* " meaning broken or weakened, in reference to the broken band of silver pollinosity on T5, which appears as two pollinose spots.

#### Distribution

Costa Rica, ACG (Prov. Guanacaste), 900 m.

#### Ecology

*Uramya infracta* has been reared only once, from a total of 153 wild-caught non-sibling *Natada fusca* Druce (Limacodidae) caterpillars in ACG rain forest.

### Uramya lativittata

Fleming & Wood sp. n.

urn:lsid:zoobank.org:act:816D6875-4B00-4405-9902-10B58A2153F6

#### Materials

- **Type status:** Holotype. **Occurrence:** occurrenceDetails: http://janzen.sas.upenn.edu; catalogNumber: DHJPAR0011557; recordedBy: D.H. Janzen, W. Hallwachs, Harry Ramirez; individualID: DHJPAR0011557; individualCount: 1; sex: M; lifeStage: adult; preparations: pinned; otherCatalogNumbers: ASTAQ944-06, 04-SRNP-48701; **Taxon:** scientificName: Uramya lativittata; phylum: Arthropoda; class: Insecta; order: Diptera; family: Tachinidae; genus: Uramya; specificEpithet: lativittata; scientificNameAuthorship: Fleming & Wood; **Location:** continent: Central America; country: Costa Rica; countryCode: CR; stateProvince: Guanacaste; county: Area de Conservación Guanacaste; locality: Sector Cacao; verbatimLocality: Gongora Bananal; verbatimElevation: 600; verbatimLatitude: 10.889; verbatimLongitude: -85.476; verbatimCoordinateSystem: Decimal; decimalLatitude: 10.889; decimalLongitude: -85.476; **Identification:** identifiedBy: AJ Fleming; dateIdentified: 2015; **Event:** samplingProtocol: reared from caterpillar of *Megalopyge albicollis* (Megalopygidae); verbatimEventDate: 02-Oct-2004; **Record Level:** language: en; institutionCode: CNC; collectionCode: Insects; basisOfRecord: Pinned Specimen
- **Type status:** Paratype. **Occurrence:** occurrenceDetails: http://janzen.sas.upenn.edu; catalogNumber: DHJPAR0023645; recordedBy: D.H. Janzen, W. Hallwachs, Dunia Garcia; individualID: DHJPAR0023645; individualCount: 1; sex: F; lifeStage: adult; preparations: pinned; otherCatalogNumbers: ASTAW502-08, 07-SRNP-47386; **Taxon:** scientificName: Uramya lativittata; phylum: Arthropoda; class: Insecta; order: Diptera; family: Tachinidae; genus: Uramya; specificEpithet: lativittata; scientificNameAuthorship: Fleming & Wood; **Location:** continent: Central America; country: Costa Rica; countryCode: CR; stateProvince: Guanacaste; county: Area de Conservación Guanacaste; locality: Sector Cacao; verbatimLocality: Quebrada Otilio; verbatimElevation: 550; verbatimLatitude: 10.89; verbatimLongitude: -85.48; verbatimCoordinateSystem: Decimal; decimalLatitude: 10.89; decimalLongitude: -85.48; **Identification:** identifiedBy: AJ Fleming; dateIdentified: 2015; **Event:** samplingProtocol: reared from caterpillar of Trosia nigropunctigera (Megalopygidae); verbatimEventDate: 15-Jan-2008; **Record Level:** language: en; institutionCode: CNC; collectionCode: Insects; basisOfRecord: Pinned Specimen
- **Type status:** Paratype. **Occurrence:** occurrenceDetails: http://janzen.sas.upenn.edu; catalogNumber: DHJPAR0037434; recordedBy: D.H. Janzen, W. Hallwachs, Mariano Pereira; individualID: DHJPAR0037434; individualCount: 1; sex: M; lifeStage: adult; preparations: pinned; otherCatalogNumbers: ASHYC4179-10, 10-SRNP-55053; **Taxon:** scientificName: Uramya lativittata; phylum: Arthropoda; class: Insecta; order: Diptera; family: Tachinidae; genus: Uramya; specificEpithet: lativittata; scientificNameAuthorship: Fleming & Wood; **Location:** continent: Central America; country: Costa Rica; countryCode: CR; stateProvince: Guanacaste; county: Area de Conservación Guanacaste; locality: Sector Mundo Nuevo; verbatimLocality: Vado Miramonte; verbatimElevation: 305; verbatimLatitude: 10.772; verbatimLongitude: -85.434; verbatimCoordinateSystem: Decimal; decimalLatitude: 10.772; decimalLongitude: -85.434; **Identification:** identifiedBy: AJ Fleming; dateIdentified: 2015; **Event:** samplingProtocol: reared from caterpillar of Norape Janzen03 (Megalopygidae); verbatimEventDate: 04-Feb-2010; **Record Level:** language: en; institutionCode: CNC; collectionCode: Insects; basisOfRecord: Pinned Specimen
- **Type status:** Paratype. **Occurrence:** occurrenceDetails: http://janzen.sas.upenn.edu; catalogNumber: DHJPAR0034603; recordedBy: D.H. Janzen, W. Hallwachs, Dinia Martinez; individualID: DHJPAR0034603; individualCount: 1; sex: M; lifeStage: adult; preparations: pinned; otherCatalogNumbers: ASHYC1255-09, 09-SRNP-30931; **Taxon:** scientificName: Uramya lativittata; phylum: Arthropoda; class: Insecta; order: Diptera; family: Tachinidae; genus: Uramya; specificEpithet: lativittata; scientificNameAuthorship: Fleming & Wood; **Location:** continent: Central America; country: Costa Rica; countryCode: CR; stateProvince: Guanacaste; county: Area de Conservación Guanacaste; locality: Sector Pitilla; verbatimLocality: Estacion Quica; verbatimElevation: 470; verbatimLatitude: 10.997; verbatimLongitude: -85.397; verbatimCoordinateSystem: Decimal; decimalLatitude: 10.997; decimalLongitude: -85.397; **Identification:** identifiedBy: AJ Fleming; dateIdentified: 2015; **Event:** samplingProtocol: reared from caterpillar of Megalopyge dyari (Megalopygidae); verbatimEventDate: 15-Apr-2009; **Record Level:** language: en; institutionCode: CNC; collectionCode: Insects; basisOfRecord: Pinned Specimen
- **Type status:** Paratype. **Occurrence:** occurrenceDetails: http://janzen.sas.upenn.edu; catalogNumber: DHJPAR0010328; recordedBy: D.H. Janzen, W. Hallwachs, Jose Perez; individualID: DHJPAR0010328; individualCount: 1; sex: M; lifeStage: adult; preparations: pinned; otherCatalogNumbers: ASTAS159-06, 06-SRNP-42351; **Taxon:** scientificName: Uramya lativittata; phylum: Arthropoda; class: Insecta; order: Diptera; family: Tachinidae; genus: Uramya; specificEpithet: lativittata; scientificNameAuthorship: Fleming & Wood; **Location:** continent: Central America; country: Costa Rica; countryCode: CR; stateProvince: Alajuela; county: Area de Conservación Guanacaste; locality: Sector Rincon Rain Forest; verbatimLocality: Vochysia; verbatimElevation: 320; verbatimLatitude: 10.867; verbatimLongitude: -85.245; verbatimCoordinateSystem: Decimal; decimalLatitude: 10.867; decimalLongitude: -85.245; **Identification:** identifiedBy: AJ Fleming; dateIdentified: 2015; **Event:** samplingProtocol: reared from caterpillar of Trosia nigropunctigera (Megalopygidae); verbatimEventDate: 25-Jul-2006; **Record Level:** language: en; institutionCode: CNC; collectionCode: Insects; basisOfRecord: Pinned Specimen
- **Type status:** Paratype. **Occurrence:** occurrenceDetails: http://janzen.sas.upenn.edu; catalogNumber: DHJPAR0018606; recordedBy: D.H. Janzen, W. Hallwachs, Minor Carmona; individualID: DHJPAR0018606; individualCount: 1; sex: M; lifeStage: adult; preparations: pinned; otherCatalogNumbers: ASTAI1253-07, 05-SRNP-40500; **Taxon:** scientificName: Uramya lativittata; phylum: Arthropoda; class: Insecta; order: Diptera; family: Tachinidae; genus: Uramya; specificEpithet: lativittata; scientificNameAuthorship: Fleming & Wood; **Location:** continent: Central America; country: Costa Rica; countryCode: CR; stateProvince: Alajuela; county: Area de Conservación Guanacaste; locality: Sector Rincon Rain Forest; verbatimLocality: Sendero Rincon; verbatimElevation: 430; verbatimLatitude: 10.896; verbatimLongitude: -85.278; verbatimCoordinateSystem: Decimal; decimalLatitude: 10.896; decimalLongitude: -85.278; **Identification:** identifiedBy: AJ Fleming; dateIdentified: 2015; **Event:** samplingProtocol: reared from caterpillar of Megalopyge dyari (Megalopygidae); verbatimEventDate: 10-Mar-2005; **Record Level:** language: en; institutionCode: CNC; collectionCode: Insects; basisOfRecord: Pinned Specimen
- **Type status:** Paratype. **Occurrence:** occurrenceDetails: http://janzen.sas.upenn.edu; catalogNumber: DHJPAR0011558; recordedBy: D.H. Janzen, W. Hallwachs, Manuel Pereira; individualID: DHJPAR0011558; individualCount: 1; sex: M; lifeStage: adult; preparations: pinned; otherCatalogNumbers: ASTAQ945-06, 04-SRNP-48830; **Taxon:** scientificName: Uramya lativittata; phylum: Arthropoda; class: Insecta; order: Diptera; family: Tachinidae; genus: Uramya; specificEpithet: lativittata; scientificNameAuthorship: Fleming & Wood; **Location:** continent: Central America; country: Costa Rica; countryCode: CR; stateProvince: Guanacaste; county: Area de Conservación Guanacaste; locality: Sector Cacao; verbatimLocality: Gongora Bananal; verbatimElevation: 600; verbatimLatitude: 10.889; verbatimLongitude: -85.476; verbatimCoordinateSystem: Decimal; decimalLatitude: 10.889; decimalLongitude: -85.476; **Identification:** identifiedBy: AJ Fleming; dateIdentified: 2015; **Event:** samplingProtocol: reared from caterpillar of Megalopyge albicollis (Megalopygidae); verbatimEventDate: 04-Oct-2004; **Record Level:** language: en; institutionCode: CNC; collectionCode: Insects; basisOfRecord: Pinned Specimen
- **Type status:** Paratype. **Occurrence:** occurrenceDetails: http://janzen.sas.upenn.edu; catalogNumber: DHJPAR0018605; recordedBy: D.H. Janzen, W. Hallwachs, Elieth Cantillano; individualID: DHJPAR0018605; individualCount: 1; sex: M; lifeStage: adult; preparations: pinned; otherCatalogNumbers: ASTAI1252-07, 04-SRNP-26973; **Taxon:** scientificName: Uramya lativittata; phylum: Arthropoda; class: Insecta; order: Diptera; family: Tachinidae; genus: Uramya; specificEpithet: lativittata; scientificNameAuthorship: Fleming & Wood; **Location:** continent: Central America; country: Costa Rica; countryCode: CR; stateProvince: Guanacaste; county: Area de Conservación Guanacaste; locality: Sector El Hacha; verbatimLocality: Sendero Tigre; verbatimElevation: 280; verbatimLatitude: 11.032; verbatimLongitude: -85.526; verbatimCoordinateSystem: Decimal; decimalLatitude: 11.032; decimalLongitude: -85.526; **Identification:** identifiedBy: AJ Fleming; dateIdentified: 2015; **Event:** samplingProtocol: reared from caterpillar of Megalopyge dyari (Megalopygidae); verbatimEventDate: 20-Jan-2005; **Record Level:** language: en; institutionCode: CNC; collectionCode: Insects; basisOfRecord: Pinned Specimen
- **Type status:** Paratype. **Occurrence:** occurrenceDetails: http://janzen.sas.upenn.edu; catalogNumber: DHJPAR0016609; recordedBy: D.H. Janzen, W. Hallwachs, Gloria Sihezar; individualID: DHJPAR0016609; individualCount: 1; sex: M; lifeStage: adult; preparations: pinned; otherCatalogNumbers: ASTAP813-07, 06-SRNP-8311; **Taxon:** scientificName: Uramya lativittata; phylum: Arthropoda; class: Insecta; order: Diptera; family: Tachinidae; genus: Uramya; specificEpithet: lativittata; scientificNameAuthorship: Fleming & Wood; **Location:** continent: Central America; country: Costa Rica; countryCode: CR; stateProvince: Alajuela; county: Area de Conservación Guanacaste; locality: Sector San Cristobal; verbatimLocality: Corrales Viejos; verbatimElevation: 495; verbatimLatitude: 10.9; verbatimLongitude: -85.381; verbatimCoordinateSystem: Decimal; decimalLatitude: 10.9; decimalLongitude: -85.381; **Identification:** identifiedBy: AJ Fleming; dateIdentified: 2015; **Event:** samplingProtocol: reared from caterpillar of Trosia nigropunctigera (Megalopygidae); verbatimEventDate: 13-Nov-2006; **Record Level:** language: en; institutionCode: CNC; collectionCode: Insects; basisOfRecord: Pinned Specimen
- **Type status:** Paratype. **Occurrence:** occurrenceDetails: http://janzen.sas.upenn.edu; catalogNumber: DHJPAR0024634; recordedBy: D.H. Janzen, W. Hallwachs, Marta Acosta; individualID: DHJPAR0024634; individualCount: 1; sex: M; lifeStage: adult; preparations: pinned; otherCatalogNumbers: ASTAW744-08, 08-SRNP-70295; **Taxon:** scientificName: Uramya lativittata; phylum: Arthropoda; class: Insecta; order: Diptera; family: Tachinidae; genus: Uramya; specificEpithet: lativittata; scientificNameAuthorship: Fleming & Wood; **Location:** continent: Central America; country: Costa Rica; countryCode: CR; stateProvince: Guanacaste; county: Area de Conservación Guanacaste; locality: Sector Pitilla; verbatimLocality: Lobo; verbatimElevation: 520; verbatimLatitude: 10.996; verbatimLongitude: -85.406; verbatimCoordinateSystem: Decimal; decimalLatitude: 10.996; decimalLongitude: -85.406; **Identification:** identifiedBy: AJ Fleming; dateIdentified: 2015; **Event:** samplingProtocol: reared from caterpillar of Podalia orsilocha (Megalopygidae); verbatimEventDate: 20-May-2008; **Record Level:** language: en; institutionCode: CNC; collectionCode: Insects; basisOfRecord: Pinned Specimen
- **Type status:** Paratype. **Occurrence:** occurrenceDetails: http://janzen.sas.upenn.edu; catalogNumber: DHJPAR0034614; recordedBy: D.H. Janzen, W. Hallwachs, Manuel Rios; individualID: DHJPAR0034614; individualCount: 1; sex: F; lifeStage: adult; preparations: pinned; otherCatalogNumbers: ASHYC1266-09, 09-SRNP-31274; **Taxon:** scientificName: Uramya lativittata; phylum: Arthropoda; class: Insecta; order: Diptera; family: Tachinidae; genus: Uramya; specificEpithet: lativittata; scientificNameAuthorship: Fleming & Wood; **Location:** continent: Central America; country: Costa Rica; countryCode: CR; stateProvince: Guanacaste; county: Area de Conservación Guanacaste; locality: Sector Pitilla; verbatimLocality: Sendero Naciente; verbatimElevation: 700; verbatimLatitude: 10.987; verbatimLongitude: -85.428; verbatimCoordinateSystem: Decimal; decimalLatitude: 10.987; decimalLongitude: -85.428; **Identification:** identifiedBy: AJ Fleming; dateIdentified: 2015; **Event:** samplingProtocol: reared from caterpillar of Podalia orsilocha (Megalopygidae); verbatimEventDate: 28-May-2009; **Record Level:** language: en; institutionCode: CNC; collectionCode: Insects; basisOfRecord: Pinned Specimen
- **Type status:** Paratype. **Occurrence:** occurrenceDetails: http://janzen.sas.upenn.edu; catalogNumber: DHJPAR0034566; recordedBy: D.H. Janzen, W. Hallwachs, Ricardo Calero; individualID: DHJPAR0034566; individualCount: 1; sex: M; lifeStage: adult; preparations: pinned; otherCatalogNumbers: ASHYC1218-09, 09-SRNP-30537; **Taxon:** scientificName: Uramya lativittata; phylum: Arthropoda; class: Insecta; order: Diptera; family: Tachinidae; genus: Uramya; specificEpithet: lativittata; scientificNameAuthorship: Fleming & Wood; **Location:** continent: Central America; country: Costa Rica; countryCode: CR; stateProvince: Guanacaste; county: Area de Conservación Guanacaste; locality: Sector Pitilla; verbatimLocality: Estacion Quica; verbatimElevation: 470; verbatimLatitude: 10.997; verbatimLongitude: -85.397; verbatimCoordinateSystem: Decimal; decimalLatitude: 10.997; decimalLongitude: -85.397; **Identification:** identifiedBy: AJ Fleming; dateIdentified: 2015; **Event:** samplingProtocol: reared from caterpillar of Megalopyge dyari (Megalopygidae); verbatimEventDate: 25-Mar-2009; **Record Level:** language: en; institutionCode: CNC; collectionCode: Insects; basisOfRecord: Pinned Specimen

#### Description

**Male** (Fig. 10): 10–15 mm. **Head** (Fig. 10b): antenna, first flagellomere all black, except orange along upper margin, adjacent to pedicel; pedicel light brown; arista 1.5X as long as first flagellomere, dark brown and minutely pubescent; palpus yellow and haired; fronto-orbital plate, parafacial and gena silver pollinose; gena bearing few fine hairs along lower margin. **Thorax** (Fig. 10a, c): entirely gray pollinose; surfaces of dorsum of thorax and scutellum with conspicuous black hairs; sternopleura, hypopleura, pteropleura, and ventral surface of abdomen black pilose; 3 katepisternal bristles; 3 postsutural supra-alar bristles, 2nd postsutural supra-alar bristle 4X as long as 1st; postpronotum bearing fine black hairs and anepisternum with fine, yellow-white hairs; scutellum bearing 1 pair of nearly indistinct discal bristles, slightly thicker than scutellar hairs; anatergite bearing a small patch of black hairs; with 2–3 pairs of strong marginal bristles in addition to basal and subapical bristles; underside of scutellum bearing a tuft of black hairs near basal marginal bristle. Legs: femora reddish-brown ground color; tibiae yellow; coxae of a reddish color; tarsi all black. Wing: dark smoky brown translucent; major wing veins strongly infuscate. **Abdomen** (Fig. 10a): 1 pair of median marginal bristles on ST1+2, T3, T4 and T5; 2 pairs of median discal bristles on T3, T4 and T5; ground color of abdomen black, with silver pollen on basal half of T3, T4, and T5; dorsal surface of tergites not covered by silver pollen, rather bearing a brown pollinosity visible under the microscope, appearing glabrous to the naked eye; no pollen of any sort on ST1+2; underside of abdomen entirely covered in silver pollen, and black hairs. **Terminalia** (Fig. 10d, e, f): sternite 5 with two broad rectangular lobes; inner edges of lobes of sternite 5 of lobes covered in dense pollinosity, and haired marginally; sternite 5 with a narrow V-shaped median cleft, 0.43X length of sternite from lobe apex to base; cercus long, thin and sharply pointed; slightly curved when viewed laterally, with a slight upward hook at its tip; distinctly tapered, with apical section 1.6X as long as upper lobe; surstylus long and digitiform in lateral view, haired along its entire length, these hairs becoming thicker basally; tip of surstylus not lobed when viewed dorsally; surstylus inwardly curved, 1.1X as long as cercus.

**Female** (Fig. 11): 7–10 mm. As male, except wings smoky amber translucent, with slight infuscation around each wing vein.

#### Diagnosis

*Uramya lativittata* can be distinguished from all other Neotropical species of *Uramya* by the following combination of traits: dark brown to black antennae, 3 postsutural supra-alar bristles, underside of scutellum with a tuft of black hairs near basal bristle, 4–5 pairs of marginal scutellar bristles, and silver pollinosity on underside of abdomen.

#### Etymology

The species epithet is derived from a combination of the latin nouns " *latus* ", meaning side, and " *vitta* ", meaning band, in reference to its distinctive abdominal pattern.

#### Distribution

Costa Rica, ACG (Provs. Guanacaste and Alajuela), 280–700 m.

#### Ecology

*Uramya lativittata* has been reared 23 times from large caterpillars belonging to the genera *Norape* Walker, *Megalopyge* Hübner, *Podalia* Walker and *Trosia* Hübner (Megalopygidae) in a sample of 2,816 non-siblings from dry, rain and dry-rain intergrade forest.

### Uramya lunula

Fleming & Wood, 2016 sp. n.

urn:lsid:zoobank.org:act:32A0B157-46DF-4509-B1D9-70F19D1BD1B3

#### Materials

- **Type status:** Holotype. **Occurrence:** occurrenceDetails: http://janzen.sas.upenn.edu; catalogNumber: DHJPAR0059536; recordedBy: D.H. Janzen, W. Hallwachs & Pablo Umana Calderon; individualID: DHJPAR0059536; individualCount: 1; sex: male; lifeStage: adult; preparations: pinned; otherCatalogNumbers: ACGBA5953-16, 16-SRNP-40422, BOLD:ADE3044; **Taxon:** scientificName: Uramya lunula; phylum: Arthropoda; class: Insecta; order: Diptera; family: Tachinidae; genus: Uramya; specificEpithet: lunula; scientificNameAuthorship: Fleming & Wood, 2016; **Location:** continent: Central America; country: Costa Rica; countryCode: CR; stateProvince: Guanacaste; county: Sector Rincon Rain Forest; locality: Area de Conservacion Guanacaste; verbatimLocality: Sendero Anonas; verbatimElevation: 405; verbatimLatitude: 10.90528; verbatimLongitude: -85.27882; verbatimCoordinateSystem: Decimal; decimalLatitude: 10.90528; decimalLongitude: -85.27882; **Identification:** identifiedBy: AJ Fleming; dateIdentified: 2016; **Event:** samplingProtocol: Reared from the larvae of the Megalopygidae, Podalia orsilocha; verbatimEventDate: 17-Jul-2016; **Record Level:** language: en; institutionCode: CNC; collectionCode: Insects; basisOfRecord: Pinned Specimen

#### Description

**Male** (Fig. 12): 10 mm. **Head** (Fig. 12b): antenna: pedicel black; arista, black and minutely pubescent; fronto-orbital plate, parafacial and gena silver pollinose; gena with few fine hairs along lower margin; facial ridge darkened; frontogenal suture black. **Thorax** (Fig. 12a, c): entirely gray pollinose; surfaces of dorsum of thorax and scutellum covered with conspicuous short black hairs; sternopleura, hypopleura, pteropleura, and ventral surface of abdomen black pilose; 3 katepisternal bristles; 3 postsutural supra-alar bristles, 2nd postsutural supra-alar 4X as long as 1st postsutural supra-alar; postpronotum and anepisternum bearing fine black hairs; scutellum bearing one pair of discal bristles; underside of scutellum bearing a tuft of black hairs near basal marginal bristle. Legs: entirely black; femora silver pollinose anterodorsally, covered in long black hairs interspersed among darker hairs and bristles. Wings: smoky gray translucent, brownish infuscate costobasally; wing veins not strongly infuscate; infuscation only visible on anterior half of R_4+5_ and M. **Abdomen** (Fig. 12a): 1 pair of median marginal bristles on ST1+2 and T3, row of marginal bristles on T4 and T5; 2 pairs of median discal bristles on T3, T4 and T5; ground color of abdomen black; posterior margin of ST1+2 silver pollinose; silver pollinose crescent-shaped markings midway along length of tergites T3 and T4; T5 bearing 2 silver pollinose blotches; underside of ST1+2, T3, and T4 silver pollinose; underside of T5 darker. **Terminalia**: not dissected, as we did not want to dissect the holotype and only known specimen of this species.

**Female**: Unknown.

#### Diagnosis

*Uramya lunula* can be distinguished from all other Neotropical species of *Uramya* by the following combination of traits: dark brown to black antennae, 3 postsutural supra-alar bristles, 2 strong lateral scutellar bristles, weak discal scutellar bristles, underside of scutellum with a tuft of black hairs near basal marginal bristle, abdomen flattened dorsoventrally, ST1+2 lacking silver pollinose spots on either side of mid-dorsal depression, T5 subtriangular, not strongly produced into a long, tail-like process, 2 pairs of median discal bristles on T3, T4 and T5, and silver pollinosity on underside of abdomen.

#### Etymology

The species epithet is derived from the Latin noun “*lunula*”, for little moon, in reference to the crescent-shaped markings present on the abdomen of this species.

#### Distribution

Costa Rica, ACG (Prov. Guanacaste), 405 m.

#### Ecology

*Uramya lunula* has been reared only once, from a *Podalia orsilocha* (Cramer) (Megalopygidae) caterpillar found in ACG rain forest. *Podalia orsilocha* has been reared 175 times from sibling and non-sibling groups in ACG.

### Uramya nitida

Fleming & Wood sp. n.

urn:lsid:zoobank.org:act:CCC8FDFB-32F0-4F61-95DF-2904F4DF455E

#### Materials

- **Type status:** Holotype. **Occurrence:** occurrenceDetails: http://janzen.sas.upenn.edu; catalogNumber: DHJPAR0029599; recordedBy: D.H. Janzen, W. Hallwachs, Petrona Rios; individualID: DHJPAR0029599; individualCount: 1; sex: M; lifeStage: adult; preparations: pinned; otherCatalogNumbers: ASHYM1020-09, 08-SRNP-36272; **Taxon:** scientificName: Uramya nitida; phylum: Arthropoda; class: Insecta; order: Diptera; family: Tachinidae; genus: Uramya; specificEpithet: nitida; scientificNameAuthorship: Fleming & Wood; **Location:** continent: Central America; country: Costa Rica; countryCode: CR; stateProvince: Guanacaste; county: Area de Conservación Guanacaste; locality: Sector Cacao; verbatimLocality: Sendero Ponderosa; verbatimElevation: 1060; verbatimLatitude: 10.915; verbatimLongitude: -85.463; verbatimCoordinateSystem: Decimal; decimalLatitude: 10.915; decimalLongitude: -85.463; **Identification:** identifiedBy: AJ Fleming; dateIdentified: 2015; **Event:** samplingProtocol: reared from caterpillar of Parasa sandrae (Limacodidae); verbatimEventDate: 04-Sep-2008; **Record Level:** language: en; institutionCode: CNC; collectionCode: Insects; basisOfRecord: Pinned Specimen
- **Type status:** Paratype. **Occurrence:** occurrenceDetails: http://janzen.sas.upenn.edu; catalogNumber: DHJPAR0018576; recordedBy: D.H. Janzen, W. Hallwachs, Roster Moraga; individualID: DHJPAR0018576; individualCount: 1; sex: F; lifeStage: adult; preparations: pinned; otherCatalogNumbers: ASTAI1223-07, 97-SRNP-11012; **Taxon:** scientificName: Uramya nitida; phylum: Arthropoda; class: Insecta; order: Diptera; family: Tachinidae; genus: Uramya; specificEpithet: nitida; scientificNameAuthorship: Fleming & Wood; **Location:** continent: Central America; country: Costa Rica; countryCode: CR; stateProvince: Guanacaste; county: Area de Conservación Guanacaste; locality: Sector Cacao; verbatimLocality: Sendero Circular; verbatimElevation: 1185; verbatimLatitude: 10.927; verbatimLongitude: -85.467; verbatimCoordinateSystem: Decimal; decimalLatitude: 10.927; decimalLongitude: -85.467; **Identification:** identifiedBy: AJ Fleming; dateIdentified: 2015; **Event:** samplingProtocol: reared from caterpillar of Parasa sandrae (Limacodidae); verbatimEventDate: 20-Dec-1997; **Record Level:** language: en; institutionCode: CNC; collectionCode: Insects; basisOfRecord: Pinned Specimen
- **Type status:** Paratype. **Occurrence:** occurrenceDetails: http://janzen.sas.upenn.edu; catalogNumber: DHJPAR0018579; recordedBy: D.H. Janzen, W. Hallwachs, Petrona Rios; individualID: DHJPAR0018579; individualCount: 1; sex: M; lifeStage: adult; preparations: pinned; otherCatalogNumbers: ASTAI1226-07, 99-SRNP-702; **Taxon:** scientificName: Uramya nitida; phylum: Arthropoda; class: Insecta; order: Diptera; family: Tachinidae; genus: Uramya; specificEpithet: nitida; scientificNameAuthorship: Fleming & Wood; **Location:** continent: Central America; country: Costa Rica; countryCode: CR; stateProvince: Guanacaste; county: Area de Conservación Guanacaste; locality: Sector Cacao; verbatimLocality: Sendero Derrumbe; verbatimElevation: 1220; verbatimLatitude: 10.929; verbatimLongitude: -85.464; verbatimCoordinateSystem: Decimal; decimalLatitude: 10.929; decimalLongitude: -85.464; **Identification:** identifiedBy: AJ Fleming; dateIdentified: 2015; **Event:** samplingProtocol: reared from caterpillar of Parasa sandrae (Limacodidae); verbatimEventDate: 22-Jun-1999; **Record Level:** language: en; institutionCode: CNC; collectionCode: Insects; basisOfRecord: Pinned Specimen
- **Type status:** Paratype. **Occurrence:** occurrenceDetails: http://janzen.sas.upenn.edu; catalogNumber: DHJPAR0018575; recordedBy: D.H. Janzen, W. Hallwachs, Dunia Garcia; individualID: DHJPAR0018575; individualCount: 1; sex: F; lifeStage: adult; preparations: pinned; otherCatalogNumbers: ASTAI1222-07, 97-SRNP-1516; **Taxon:** scientificName: Uramya nitida; phylum: Arthropoda; class: Insecta; order: Diptera; family: Tachinidae; genus: Uramya; specificEpithet: nitida; scientificNameAuthorship: Fleming & Wood; **Location:** continent: Central America; country: Costa Rica; countryCode: CR; stateProvince: Guanacaste; county: Area de Conservación Guanacaste; locality: Sector Cacao; verbatimLocality: Sendero Derrumbe; verbatimElevation: 1220; verbatimLatitude: 10.929; verbatimLongitude: -85.464; verbatimCoordinateSystem: Decimal; decimalLatitude: 10.929; decimalLongitude: -85.464; **Identification:** identifiedBy: AJ Fleming; dateIdentified: 2015; **Event:** samplingProtocol: reared from caterpillar of Parasa sandrae (Limacodidae); verbatimEventDate: 12-Aug-1997; **Record Level:** language: en; institutionCode: CNC; collectionCode: Insects; basisOfRecord: Pinned Specimen
- **Type status:** Paratype. **Occurrence:** occurrenceDetails: http://janzen.sas.upenn.edu; catalogNumber: DHJPAR0038687; recordedBy: D.H. Janzen, W. Hallwachs, Harry Ramirez; individualID: DHJPAR0038687; individualCount: 1; sex: F; lifeStage: adult; preparations: pinned; otherCatalogNumbers: ASHYD2260-10, 10-SRNP-30531; **Taxon:** scientificName: Uramya nitida; phylum: Arthropoda; class: Insecta; order: Diptera; family: Tachinidae; genus: Uramya; specificEpithet: nitida; scientificNameAuthorship: Fleming & Wood; **Location:** continent: Central America; country: Costa Rica; countryCode: CR; stateProvince: Guanacaste; county: Area de Conservación Guanacaste; locality: Sector Pitilla; verbatimLocality: Sendero Laguna; verbatimElevation: 680; verbatimLatitude: 10.989; verbatimLongitude: -85.423; verbatimCoordinateSystem: Decimal; decimalLatitude: 10.989; decimalLongitude: -85.423; **Identification:** identifiedBy: AJ Fleming; dateIdentified: 2015; **Event:** samplingProtocol: reared from caterpillar of Parasa sandrae (Limacodidae); verbatimEventDate: 24-Mar-2010; **Record Level:** language: en; institutionCode: CNC; collectionCode: Insects; basisOfRecord: Pinned Specimen
- **Type status:** Paratype. **Occurrence:** occurrenceDetails: http://janzen.sas.upenn.edu; catalogNumber: DHJPAR0050588; recordedBy: D.H. Janzen, W. Hallwachs, Calixto Moraga; individualID: DHJPAR0050588; individualCount: 1; sex: M; lifeStage: adult; preparations: pinned; otherCatalogNumbers: ACGBA3180-13, 13-SRNP-30042; **Taxon:** scientificName: Uramya nitida; phylum: Arthropoda; class: Insecta; order: Diptera; family: Tachinidae; genus: Uramya; specificEpithet: nitida; scientificNameAuthorship: Fleming & Wood; **Location:** continent: Central America; country: Costa Rica; countryCode: CR; stateProvince: Guanacaste; county: Area de Conservación Guanacaste; locality: Sector Pitilla; verbatimLocality: Sendero Evangelista; **Identification:** identifiedBy: AJ Fleming; dateIdentified: 2015; **Event:** samplingProtocol: reared from caterpillar of Parasa sandrae (Limacodidae); verbatimEventDate: 11-Feb-2013; **Record Level:** language: en; institutionCode: CNC; collectionCode: Insects; basisOfRecord: Pinned Specimen
- **Type status:** Paratype. **Occurrence:** occurrenceDetails: http://janzen.sas.upenn.edu; catalogNumber: DHJPAR0018577; recordedBy: D.H. Janzen, W. Hallwachs, Petrona Rios; individualID: DHJPAR0018577; individualCount: 1; sex: F; lifeStage: adult; preparations: pinned; otherCatalogNumbers: ASTAI1224-07, 97-SRNP-1483; **Taxon:** scientificName: Uramya nitida; phylum: Arthropoda; class: Insecta; order: Diptera; family: Tachinidae; genus: Uramya; specificEpithet: nitida; scientificNameAuthorship: Fleming & Wood; **Location:** continent: Central America; country: Costa Rica; countryCode: CR; stateProvince: Guanacaste; county: Area de Conservación Guanacaste; locality: Sector Cacao; verbatimLocality: Sendero Nayo; verbatimElevation: 1090; verbatimLatitude: 10.924; verbatimLongitude: -85.47; verbatimCoordinateSystem: Decimal; decimalLatitude: 10.924; decimalLongitude: -85.47; **Identification:** identifiedBy: AJ Fleming; dateIdentified: 2015; **Event:** samplingProtocol: reared from caterpillar of Parasa sandrae (Limacodidae); verbatimEventDate: 09-Aug-1997; **Record Level:** language: en; institutionCode: CNC; collectionCode: Insects; basisOfRecord: Pinned Specimen
- **Type status:** Paratype. **Occurrence:** occurrenceDetails: http://janzen.sas.upenn.edu; catalogNumber: DHJPAR0016677; recordedBy: D.H. Janzen, W. Hallwachs, Petrona Rios; individualID: DHJPAR0016677; individualCount: 1; sex: F; lifeStage: adult; preparations: pinned; otherCatalogNumbers: ASTAP982-07, 06-SRNP-36559; **Taxon:** scientificName: Uramya nitida; phylum: Arthropoda; class: Insecta; order: Diptera; family: Tachinidae; genus: Uramya; specificEpithet: nitida; scientificNameAuthorship: Fleming & Wood; **Location:** continent: Central America; country: Costa Rica; countryCode: CR; stateProvince: Guanacaste; county: Area de Conservación Guanacaste; locality: Sector Cacao; verbatimLocality: Sendero Abajo; verbatimElevation: 1020; verbatimLatitude: 10.925; verbatimLongitude: -85.472; verbatimCoordinateSystem: Decimal; decimalLatitude: 10.925; decimalLongitude: -85.472; **Identification:** identifiedBy: AJ Fleming; dateIdentified: 2015; **Event:** samplingProtocol: reared from caterpillar of Parasa sandrae (Limacodidae); verbatimEventDate: 21-Nov-2006; **Record Level:** language: en; institutionCode: CNC; collectionCode: Insects; basisOfRecord: Pinned Specimen
- **Type status:** Paratype. **Occurrence:** occurrenceDetails: http://janzen.sas.upenn.edu; catalogNumber: DHJPAR0018582; recordedBy: D.H. Janzen, W. Hallwachs, Petrona Rios; individualID: DHJPAR0018582; individualCount: 1; sex: M; lifeStage: adult; preparations: pinned; otherCatalogNumbers: ASTAI1229-07, 98-SRNP-3111; **Taxon:** scientificName: Uramya nitida; phylum: Arthropoda; class: Insecta; order: Diptera; family: Tachinidae; genus: Uramya; specificEpithet: nitida; scientificNameAuthorship: Fleming & Wood; **Location:** continent: Central America; country: Costa Rica; countryCode: CR; stateProvince: Guanacaste; county: Area de Conservación Guanacaste; locality: Sector Cacao; verbatimLocality: Sendero Nayo; verbatimElevation: 1090; verbatimLatitude: 10.924; verbatimLongitude: -85.47; verbatimCoordinateSystem: Decimal; decimalLatitude: 10.924; decimalLongitude: -85.47; **Identification:** identifiedBy: AJ Fleming; dateIdentified: 2015; **Event:** samplingProtocol: reared from caterpillar of Parasa sandrae (Limacodidae); verbatimEventDate: 12-Aug-1998; **Record Level:** language: en; institutionCode: CNC; collectionCode: Insects; basisOfRecord: Pinned Specimen
- **Type status:** Paratype. **Occurrence:** occurrenceDetails: http://janzen.sas.upenn.edu; catalogNumber: DHJPAR0046670; recordedBy: D.H. Janzen, W. Hallwachs, Petrona Rios; individualID: DHJPAR0046670; individualCount: 1; sex: M; lifeStage: adult; preparations: pinned; otherCatalogNumbers: ACGBA843-12, 11-SRNP-33075; **Taxon:** scientificName: Uramya nitida; phylum: Arthropoda; class: Insecta; order: Diptera; family: Tachinidae; genus: Uramya; specificEpithet: nitida; scientificNameAuthorship: Fleming & Wood; **Location:** continent: Central America; country: Costa Rica; countryCode: CR; stateProvince: Guanacaste; county: Area de Conservación Guanacaste; locality: Sector Pitilla; verbatimLocality: Sendero Evangelista; verbatimElevation: 660; verbatimLatitude: 10.987; verbatimLongitude: -85.421; verbatimCoordinateSystem: Decimal; decimalLatitude: 10.987; decimalLongitude: -85.421; **Identification:** identifiedBy: AJ Fleming; dateIdentified: 2015; **Event:** samplingProtocol: reared from caterpillar of Parasa sandrae (Limacodidae); verbatimEventDate: 07-Nov-2011; **Record Level:** language: en; institutionCode: CNC; collectionCode: Insects; basisOfRecord: Pinned Specimen
- **Type status:** Paratype. **Occurrence:** occurrenceDetails: http://janzen.sas.upenn.edu; catalogNumber: DHJPAR0018578; recordedBy: D.H. Janzen, W. Hallwachs, Petrona Rios; individualID: DHJPAR0018578; individualCount: 1; sex: M; lifeStage: adult; preparations: pinned; otherCatalogNumbers: ASTAI1225-07, 02-SRNP-23551; **Taxon:** scientificName: Uramya nitida; phylum: Arthropoda; class: Insecta; order: Diptera; family: Tachinidae; genus: Uramya; specificEpithet: nitida; scientificNameAuthorship: Fleming & Wood; **Location:** continent: Central America; country: Costa Rica; countryCode: CR; stateProvince: Guanacaste; county: Area de Conservación Guanacaste; locality: Sector Cacao; verbatimLocality: Sendero Derrumbe; verbatimElevation: 1220; verbatimLatitude: 10.929; verbatimLongitude: -85.464; verbatimCoordinateSystem: Decimal; decimalLatitude: 10.929; decimalLongitude: -85.464; **Identification:** identifiedBy: AJ Fleming; dateIdentified: 2015; **Event:** samplingProtocol: reared from caterpillar of Parasa sandrae (Limacodidae); verbatimEventDate: 18-Sep-2002; **Record Level:** language: en; institutionCode: CNC; collectionCode: Insects; basisOfRecord: Pinned Specimen
- **Type status:** Paratype. **Occurrence:** occurrenceDetails: http://janzen.sas.upenn.edu; catalogNumber: DHJPAR0018580; recordedBy: D.H. Janzen, W. Hallwachs, Petrona Rios; individualID: DHJPAR0018580; individualCount: 1; sex: M; lifeStage: adult; preparations: pinned; otherCatalogNumbers: ASTAI1227-07, 98-SRNP-3207; **Taxon:** scientificName: Uramya nitida; phylum: Arthropoda; class: Insecta; order: Diptera; family: Tachinidae; genus: Uramya; specificEpithet: nitida; scientificNameAuthorship: Fleming & Wood; **Location:** continent: Central America; country: Costa Rica; countryCode: CR; stateProvince: Guanacaste; county: Area de Conservación Guanacaste; locality: Sector Cacao; verbatimLocality: Sendero Circular; verbatimElevation: 1185; verbatimLatitude: 10.927; verbatimLongitude: -85.467; verbatimCoordinateSystem: Decimal; decimalLatitude: 10.927; decimalLongitude: -85.467; **Identification:** identifiedBy: AJ Fleming; dateIdentified: 2015; **Event:** samplingProtocol: reared from caterpillar of Parasa sandrae (Limacodidae); verbatimEventDate: 19-Aug-1998; **Record Level:** language: en; institutionCode: CNC; collectionCode: Insects; basisOfRecord: Pinned Specimen
- **Type status:** Paratype. **Occurrence:** occurrenceDetails: http://janzen.sas.upenn.edu; catalogNumber: DHJPAR0018581; recordedBy: D.H. Janzen, W. Hallwachs, Petrona Rios; individualID: DHJPAR0018581; individualCount: 1; sex: M; lifeStage: adult; preparations: pinned; otherCatalogNumbers: ASTAI1228-07, 02-SRNP-23316; **Taxon:** scientificName: Uramya nitida; phylum: Arthropoda; class: Insecta; order: Diptera; family: Tachinidae; genus: Uramya; specificEpithet: nitida; scientificNameAuthorship: Fleming & Wood; **Location:** continent: Central America; country: Costa Rica; countryCode: CR; stateProvince: Guanacaste; county: Area de Conservación Guanacaste; locality: Sector Cacao; verbatimLocality: Sendero Derrumbe; verbatimElevation: 1220; verbatimLatitude: 10.929; verbatimLongitude: -85.464; verbatimCoordinateSystem: Decimal; decimalLatitude: 10.929; decimalLongitude: -85.464; **Identification:** identifiedBy: AJ Fleming; dateIdentified: 2015; **Event:** samplingProtocol: reared from caterpillar of Parasa sandrae (Limacodidae); verbatimEventDate: 19-Aug-2002; **Record Level:** language: en; institutionCode: CNC; collectionCode: Insects; basisOfRecord: Pinned Specimen
- **Type status:** Paratype. **Occurrence:** occurrenceDetails: http://janzen.sas.upenn.edu; catalogNumber: DHJPAR0027843; recordedBy: D.H. Janzen, W. Hallwachs, Petrona Rios; individualID: DHJPAR0027843; individualCount: 1; sex: F; lifeStage: adult; preparations: pinned; otherCatalogNumbers: ASHYE080-08, 08-SRNP-35937; **Taxon:** scientificName: Uramya nitida; phylum: Arthropoda; class: Insecta; order: Diptera; family: Tachinidae; genus: Uramya; specificEpithet: nitida; scientificNameAuthorship: Fleming & Wood; **Location:** continent: Central America; country: Costa Rica; countryCode: CR; stateProvince: Guanacaste; county: Area de Conservación Guanacaste; locality: Sector Cacao; verbatimLocality: Sendero Circular; verbatimElevation: 1185; verbatimLatitude: 10.927; verbatimLongitude: -85.467; verbatimCoordinateSystem: Decimal; decimalLatitude: 10.927; decimalLongitude: -85.467; **Identification:** identifiedBy: AJ Fleming; dateIdentified: 2015; **Event:** samplingProtocol: reared from caterpillar of Parasa sandrae (Limacodidae); verbatimEventDate: 13-Aug-2008; **Record Level:** language: en; institutionCode: CNC; collectionCode: Insects; basisOfRecord: Pinned Specimen

#### Description

**Male** (Fig. 13): 5–8 mm. **Head** (Fig. 13b): antenna: first flagellomere ranging in color from dark orange to light brown, slighlty lighter on upper third, adjacent to pedicel; pedicel orange; arista 1.5X as long as first flagellomere, dark brown and minutely pubescent; palpus dark yellow, and haired; fronto-orbital plate, parafacial and gena silver pollinose; gena silver pollinose with few fine hairs along lower margin. **Thorax** (Fig. 13a, c): entirely gray pollinose; dorsum of thorax and scutellum black pilose; sternopleura, hypopleura, pteropleura, and ventral surface of abdomen yellow-white pilose; 2 katepisternal bristles; 3 postsutural supra-alar bristles; postpronotum and anepisternum bearing fine black hairs; scutellum bearing 2 discal bristles; underside of scutellum bearing a tuft of black hairs near basal marginal bristle. Legs: coxae orange/yellow, fore femur gray pollinose, with a light covering of thin dark hairs; ground color of femora black basally and yellow apically; mid and hind legs of yellow ground color. Wings: smoky gray translucent; veins not infuscate. **Abdomen** (Fig. 13a): 1 pair of median marginal bristles on ST1+2; row of marginal bristles on T3 and T4; median discal bristles on T3, T4 and a row on T5; abdomen brown black dorsally, lighter brown laterally; ventral margins of tergites bearing a light yellow fringe; posterior margins of T3 and T4 with gray pollinosity. **Terminalia** (Fig. 13d, e, f): terminalia yellow and visible in pinned specimens; sternite 5 with two small lobes; inner margin covered in dense pollinosity, appearing darker than surrounding cuticle; apical edges of lobes of sternite 5 bearing many medium-length, stout bristles interspersed with longer bristles close to apical margin; sternite 5 with wide V-shaped median cleft, 0.6X length of sternite from lobe apex to base; cercus sharply pointed, distinctly tapered; apical section of cercus 2.4X as long as upper lobe; not strongly curved when viewed laterally, with only a slight upward hook at its tip; surstylus oblong, curved and scythe-like in lateral view, with short bristles covering almost its entire surface, and with tips not lobed when viewed dorsally; surstyli not angled inwards, parallel; surstylus 1.2X as long as cercus.

**Female** (Fig. 14): 6–7 mm. As male, except first flagellomere and pedicel bright orange, and legs varying from yellow to orange.

#### Diagnosis

*Uramya nitida* can be distinguished from all other Neotropical species of *Uramya* by the following combination of traits: light-colored, orange-brown antennae, 2 postsutural supra-alar bristles, underside of scutellum with a tuft of black hairs near basal marginal bristle, 1 pair of median marginal bristles on ST1+2, and a row of marginal bristles on T3 and T4.

#### Etymology

*Uramya nitida* is derived from the Latin noun "*nitidus*", meaning bright or glossy, in reference to the glossy appearance of the abdomen under certain angles of light.

#### Distribution

Costa Rica, ACG (Prov. Guanacaste), 660–1220 m.

#### Ecology

*Uramya nitida* has been reared 18 times from *Parasa sandrae* Corrales & Epstein (Limacodidae), in a sample of 497 non-sibling, wild-caught larvae in both cloud and rain forest.

### Uramya pannosa

Fleming & Wood sp. n.

urn:lsid:zoobank.org:act:37EB49A9-2CAB-4297-A451-B391B7D2634C

#### Materials

- **Type status:** Holotype. **Occurrence:** occurrenceDetails: http://janzen.sas.upenn.edu; catalogNumber: DHJPAR0018572; recordedBy: D.H. Janzen, W. Hallwachs, Harry Ramirez; individualID: DHJPAR0018572; individualCount: 1; sex: M; lifeStage: adult; preparations: pinned; otherCatalogNumbers: ASTAI1219-07, 02-SRNP-23893; **Taxon:** scientificName: Uramya pannosa; phylum: Arthropoda; class: Insecta; order: Diptera; family: Tachinidae; genus: Uramya; specificEpithet: pannosa; scientificNameAuthorship: Fleming & Wood; **Location:** continent: Central America; country: Costa Rica; countryCode: CR; stateProvince: Guanacaste; county: Area de Conservación Guanacaste; locality: Sector Cacao; verbatimLocality: Sendero Toma Agua; verbatimElevation: 1140; verbatimLatitude: 10.928; verbatimLongitude: -85.467; verbatimCoordinateSystem: Decimal; decimalLatitude: 10.928; decimalLongitude: -85.467; **Identification:** identifiedBy: AJ Fleming; dateIdentified: 2015; **Event:** samplingProtocol: reared from caterpillar of Parasa macrodonta (Limacodidae); verbatimEventDate: 01-Dec-2002; **Record Level:** language: en; institutionCode: CNC; collectionCode: Insects; basisOfRecord: Pinned Specimen
- **Type status:** Paratype. **Occurrence:** occurrenceDetails: http://janzen.sas.upenn.edu; catalogNumber: DHJPAR0018571; recordedBy: D.H. Janzen, W. Hallwachs, Roster Moraga; individualID: DHJPAR0018571; individualCount: 1; sex: F; lifeStage: adult; preparations: pinned; otherCatalogNumbers: ASTAI1218-07, 97-SRNP-11106; **Taxon:** scientificName: Uramya pannosa; phylum: Arthropoda; class: Insecta; order: Diptera; family: Tachinidae; genus: Uramya; specificEpithet: pannosa; scientificNameAuthorship: Fleming & Wood; **Location:** continent: Central America; country: Costa Rica; countryCode: CR; stateProvince: Guanacaste; county: Area de Conservación Guanacaste; locality: Sector Cacao; verbatimLocality: Sendero Derrumbe; verbatimElevation: 1220; verbatimLatitude: 10.929; verbatimLongitude: -85.464; verbatimCoordinateSystem: Decimal; decimalLatitude: 10.929; decimalLongitude: -85.464; **Identification:** identifiedBy: AJ Fleming; dateIdentified: 2015; **Event:** samplingProtocol: reared from caterpillar of Parasa macrodonta (Limacodidae); verbatimEventDate: 10-Jan-1998; **Record Level:** language: en; institutionCode: CNC; collectionCode: Insects; basisOfRecord: Pinned Specimen
- **Type status:** Paratype. **Occurrence:** occurrenceDetails: http://janzen.sas.upenn.edu; catalogNumber: DHJPAR0042603; recordedBy: D.H. Janzen, W. Hallwachs, Dunia Garcia; individualID: DHJPAR0042603; individualCount: 1; sex: F; lifeStage: adult; preparations: pinned; otherCatalogNumbers: ASHYH361-11, 11-SRNP-35195; **Taxon:** scientificName: Uramya pannosa; phylum: Arthropoda; class: Insecta; order: Diptera; family: Tachinidae; genus: Uramya; specificEpithet: pannosa; scientificNameAuthorship: Fleming & Wood; **Location:** continent: Central America; country: Costa Rica; countryCode: CR; stateProvince: Guanacaste; county: Area de Conservación Guanacaste; locality: Sector Cacao; verbatimLocality: Sendero Derrumbe; verbatimElevation: 1220; verbatimLatitude: 10.929; verbatimLongitude: -85.464; verbatimCoordinateSystem: Decimal; decimalLatitude: 10.929; decimalLongitude: -85.464; **Identification:** identifiedBy: AJ Fleming; dateIdentified: 2015; **Event:** samplingProtocol: reared from caterpillar of Parasa macrodonta (Limacodidae); verbatimEventDate: 12-Apr-2011; **Record Level:** language: en; institutionCode: CNC; collectionCode: Insects; basisOfRecord: Pinned Specimen
- **Type status:** Paratype. **Occurrence:** occurrenceDetails: http://janzen.sas.upenn.edu; catalogNumber: DHJPAR0018574; recordedBy: D.H. Janzen, W. Hallwachs, Petrona Rios; individualID: DHJPAR0018574; individualCount: 1; sex: F; lifeStage: adult; preparations: pinned; otherCatalogNumbers: ASTAI1221-07, 05-SRNP-30344; **Taxon:** scientificName: Uramya pannosa; phylum: Arthropoda; class: Insecta; order: Diptera; family: Tachinidae; genus: Uramya; specificEpithet: pannosa; scientificNameAuthorship: Fleming & Wood; **Location:** continent: Central America; country: Costa Rica; countryCode: CR; stateProvince: Guanacaste; county: Area de Conservación Guanacaste; locality: Sector Pitilla; verbatimLocality: Sendero Orosilito; verbatimElevation: 900; verbatimLatitude: 10.983; verbatimLongitude: -85.436; verbatimCoordinateSystem: Decimal; decimalLatitude: 10.983; decimalLongitude: -85.436; **Identification:** identifiedBy: AJ Fleming; dateIdentified: 2015; **Event:** samplingProtocol: reared from caterpillar of Parasa macrodonta (Limacodidae); verbatimEventDate: 27-Feb-2005; **Record Level:** language: en; institutionCode: CNC; collectionCode: Insects; basisOfRecord: Pinned Specimen
- **Type status:** Paratype. **Occurrence:** occurrenceDetails: http://janzen.sas.upenn.edu; catalogNumber: DHJPAR0017074; recordedBy: D.H. Janzen, W. Hallwachs, Calixto Moraga; individualID: DHJPAR0017074; individualCount: 1; sex: F; lifeStage: adult; preparations: pinned; otherCatalogNumbers: ASTAP512-07, 06-SRNP-65874; **Taxon:** scientificName: Uramya pannosa; phylum: Arthropoda; class: Insecta; order: Diptera; family: Tachinidae; genus: Uramya; specificEpithet: pannosa; scientificNameAuthorship: Fleming & Wood; **Location:** continent: Central America; country: Costa Rica; countryCode: CR; stateProvince: Guanacaste; county: Area de Conservación Guanacaste; locality: Sector Pitilla; verbatimLocality: Sendero Orosilito; verbatimElevation: 900; verbatimLatitude: 10.983; verbatimLongitude: -85.436; verbatimCoordinateSystem: Decimal; decimalLatitude: 10.983; decimalLongitude: -85.436; **Identification:** identifiedBy: AJ Fleming; dateIdentified: 2015; **Event:** samplingProtocol: reared from caterpillar of Parasa macrodonta (Limacodidae); verbatimEventDate: 02-Mar-2007; **Record Level:** language: en; institutionCode: CNC; collectionCode: Insects; basisOfRecord: Pinned Specimen
- **Type status:** Paratype. **Occurrence:** occurrenceDetails: http://janzen.sas.upenn.edu; catalogNumber: DHJPAR0018573; recordedBy: D.H. Janzen, W. Hallwachs, Petrona Rios; individualID: DHJPAR0018573; individualCount: 1; sex: F; lifeStage: adult; preparations: pinned; otherCatalogNumbers: ASTAI1220-07, 05-SRNP-30344; **Taxon:** scientificName: Uramya pannosa; phylum: Arthropoda; class: Insecta; order: Diptera; family: Tachinidae; genus: Uramya; specificEpithet: pannosa; scientificNameAuthorship: Fleming & Wood; **Location:** continent: Central America; country: Costa Rica; countryCode: CR; stateProvince: Guanacaste; county: Area de Conservación Guanacaste; locality: Sector Pitilla; verbatimLocality: Sendero Orosilito; verbatimElevation: 900; verbatimLatitude: 10.983; verbatimLongitude: -85.436; verbatimCoordinateSystem: Decimal; decimalLatitude: 10.983; decimalLongitude: -85.436; **Identification:** identifiedBy: AJ Fleming; dateIdentified: 2015; **Event:** samplingProtocol: reared from caterpillar of Parasa macrodonta (Limacodidae); verbatimEventDate: 27-Feb-2005; **Record Level:** language: en; institutionCode: CNC; collectionCode: Insects; basisOfRecord: Pinned Specimen

#### Description

**Male** (Fig. 15): 15 mm. **Head** (Fig. 15b): antenna: first flagellomere dark brown on lower 2/3,turning to brownish orange on upper third and adjacent to pedicel; pedicel dark orange; arista 1.5X as long as first flagellomere, dark brown and minutely pubescent; palpus dark orange and haired; fronto-orbital plate, parafacial and gena silver pollinose; gena silver pollinose with a cluster of hairs along lower 1/3 to 1/2; facial ridge darkened, with few fine hairs along lower margin; frontogenal suture black. **Thorax** (Fig. 15a, c): entirely gray pollinose; dorsum of thorax and scutellum black pilose; sternopleura, hypopleura, pteropleura, and ventral surface of abdomen yellow-white pilose; 2 katepisternal bristles; 2 postsutural supra-alar bristles; postpronotum and anepisternum bearing fine black hairs; underside of scutellum bearing a tuft of black hairs near basal marginal bristle. Legs: fore femur gray pollinose, densely covered in thin dark hairs interspersed among the bristles; mid and hind tibiae dark brown to black basally, apically dark orange. Wings: smoky gray, with dense infuscation around each vein. **Abdomen** (Fig. 15a): median marginal bristles only on ST1+2; 2 pairs of median discal bristles on T3 and T4; ground color of abdomen brown-black up to tergite 5; silver pollinosity present on anteriormost half of T3, T4 and T5; ST1+2 with lateral gray pollinose spots on posterior half; band of short black hairs surrounding discal bristles on T3 and T4, abdomen otherwise appearing devoid of hairs. **Terminalia** (Fig. 15d, e, f): sternite 5 with two small lobes; inner margin covered in dense pollinosity, appearing darker than surrounding cuticle; apical edges of lobes of sternite 5 bearing many medium-length, stout bristles interspersed with longer bristles close to apical margin; sternite 5 with wide V-shaped median cleft, 0.42X length of sternite from lobe apex to base; cercus sharply pointed, distinctly tapered; apical section of cercus 1.4X as long as upper lobe; strongly curved when viewed laterally with a slight upward hook at its tip; surstylus oblong, curved and scythe-like in lateral view; posterodorsal half of surstylus haired, apex with almost no short apical bristles; tip of surstylus not lobed when viewed dorsally; surstylus strongly angled inwards, 1.4X as long as cercus.

**Female** (Fig. 16): 10–12 mm. As male, except arista 1.7X as long as first flagellomere, abdomen normally haired, median marginals present on segments ST1+2, T3, T4, and T5, and median discal bristles on T3, T4, and T5.

#### Diagnosis

*Uramya pannosa* can be distinguished from all other Neotropical species of *Uramya* by the following combination of traits: pedicel orange, 2 postsutural supra-alar bristles, underside of scutellum with a tuft of black hairs near basal marginal bristle, overall absence of abdominal hairs, only 1 pair of median marginal bristles on ST1+2, and 2 pairs of median discal bristles on T3 and T4.

#### Etymology

The species epithet is derived from the Latin adjective “ *pannosus* ”, meaining tattered or shabby, in reference to the irregular and ragged appearance of the abdominal banding.

#### Distribution

Costa Rica, ACG (Prov. Guanacaste), 900–1220 m.

#### Ecology

*Uramya pannosa* has been reared 5 times from *Parasa macrodonta* Hering & Hopp (Limacodidae) in both cloud and rain forest, and is the only species of tachinid to have been reared from this species of caterpillar in 166 non-sibling rearings.

### Uramya penicillata

Fleming & Wood sp. n.

urn:lsid:zoobank.org:act:0A577F6C-3BF0-44C7-956D-4BB87C453671

#### Materials

- **Type status:** Holotype. **Occurrence:** occurrenceDetails: http://janzen.sas.upenn.edu; catalogNumber: DHJPAR0018604; recordedBy: D.H. Janzen & W. Hallwachs, Manuel Pereira; individualID: DHJPAR0018604; individualCount: 1; sex: M; lifeStage: adult; preparations: pinned; otherCatalogNumbers: ASTAI1251-07 ,03-SRNP-23649; **Taxon:** scientificName: Uramya penicillata; phylum: Arthropoda; class: Insecta; order: Diptera; family: Tachinidae; genus: Uramya; specificEpithet: penicillata; scientificNameAuthorship: Fleming & Wood; **Location:** continent: Central America; country: Costa Rica; countryCode: CR; stateProvince: Guanacaste; county: Area de Conservación Guanacaste; locality: Sector Cacao; verbatimLocality: Sendero Cima; verbatimElevation: 1460; verbatimLatitude: 10.933; verbatimLongitude: -85.457; verbatimCoordinateSystem: Decimal; decimalLatitude: 10.933; decimalLongitude: -85.457; **Identification:** identifiedBy: AJ Fleming; dateIdentified: 2015; **Event:** samplingProtocol: reared from caterpillar of *Isochaetes dwagsi* (Limacodidae); verbatimEventDate: Dec-02-2003; **Record Level:** language: en; institutionCode: CNC; collectionCode: Insects; basisOfRecord: Pinned Specimen

#### Description

**Male** (Fig. 17): 10 mm. **Head** (Fig. 17b): antenna: pedicel orange; arista light brown and minutely pubescent; fronto-orbital plate, parafacial and gena brassy pollinose; gena with few fine hairs along lower margin; facial ridge darkened; frontogenal suture black. **Thorax** (Fig. 17a, c): entirely gray pollinose; surfaces of dorsum of thorax and scutellum covered with conspicuous short black hairs; sternopleura, hypopleura, pteropleura, and ventral surface of abdomen yellow-white pilose; 2 katepisternal bristles; 3 postsutural supra-alar bristles, 2nd postsutural supra-alar 4X as long as 1st postsutural supra-alar; postpronotum bearing fine black hairs and anepisternum with fine yellow-white hairs; scutellum bearing two pairs of discal bristles; underside of scutellum bearing a tuft of black hairs near basal marginal bristle. Legs: coxae and femora of reddish ground color, tibiae with yellow ground color; femora covered in long black hairs interspersed among darker hairs and bristles; tarsi all black. Wings: smoky gray translucent; veins not infuscate. **Abdomen** (Fig. 17a): 1 pair of median marginal bristles on ST1+2, a row of marginal bristles on T3, T4 and T5; median discal bristles on T3, T4 and T5; ground color of abdomen black, with transverse bands of silver pollen on anterior half of T3, T4, and T5; underside of abdomen entirely covered in silver pollinosity. **Terminalia**: damage to the holotype by scavengers made the terminalia unavailable; however, other diagnostic characters are sufficient to define the species.

**Female**: Unknown.

#### Diagnosis

*Uramya penicillata* is distinguished from all other Neotropical species of *Uramya* by the following combination of traits: light colored pedicel, 3 postsutural supra-alar bristles, the first of which is 4X smaller than the second, underside of scutellum with a tuft of black hairs near basal marginal bristle, rows of marginal bristles on T3 and T4, and transverse bands of silver pollinosity extending across tergites to underside of abdomen.

#### Etymology

The species epithet is derived from the Latin noun “*penicillus*”, for paintbrush, in reference to the brush-like tuft of hairs present along the underside of the scutellum in this species.

#### Distribution

Costa Rica, ACG (Prov. Guanacaste), 1460 m

#### Ecology

*Uramya penicillata* has been reared only once, from an *Isochaetes dwagsi* Corrales & Epstein (Limacodidae) caterpillar found in cloud forest. *Isochaetes dwagsi* has been reared 503 times from sibling and non-sibling groups in ACG.

### Uramya sibinivora

Guimarães, 1980

#### Materials

- **Type status:** Other material. **Occurrence:** occurrenceDetails: http://janzen.sas.upenn.edu; catalogNumber: DHJPAR0018553; recordedBy: D.H. Janzen, W. Hallwachs, gusaneros; individualID: DHJPAR0018553; individualCount: 1; sex: M; lifeStage: adult; preparations: pinned; otherCatalogNumbers: ASTAI1200-07, 93-SRNP-2131; **Taxon:** scientificName: Uramya sibinivora; phylum: Arthropoda; class: Insecta; order: Diptera; family: Tachinidae; genus: Uramya; specificEpithet: sibinivora; scientificNameAuthorship: Guimarães, 1980; **Location:** continent: Central America; country: Costa Rica; countryCode: CR; stateProvince: Guanacaste; county: Area de Conservación Guanacaste; locality: Sector Santa Rosa; verbatimLocality: Cafetal; verbatimElevation: 280; verbatimLatitude: 10.858; verbatimLongitude: -85.611; verbatimCoordinateSystem: Decimal; decimalLatitude: 10.858; decimalLongitude: -85.611; **Identification:** identifiedBy: AJ Fleming; dateIdentified: 2015; **Event:** samplingProtocol: reared from caterpillar of Parasa wellesca (Limacodidae); **Record Level:** language: en; institutionCode: CNC; collectionCode: Insects; basisOfRecord: Pinned Specimen
- **Type status:** Other material. **Occurrence:** occurrenceDetails: http://janzen.sas.upenn.edu; catalogNumber: DHJPAR0018555; recordedBy: D.H. Janzen, W. Hallwachs, Osvaldo Espinoza; individualID: DHJPAR0018555; individualCount: 1; sex: M; lifeStage: adult; preparations: pinned; otherCatalogNumbers: ASTAI1202-07, 98-SRNP-6948; **Taxon:** scientificName: Uramya sibinivora; phylum: Arthropoda; class: Insecta; order: Diptera; family: Tachinidae; genus: Uramya; specificEpithet: sibinivora; scientificNameAuthorship: Guimarães, 1980; **Location:** continent: Central America; country: Costa Rica; countryCode: CR; stateProvince: Alajuela; county: Area de Conservación Guanacaste; locality: Sector San Cristobal; verbatimLocality: Vado Rio Cucaracho; verbatimElevation: 640; verbatimLatitude: 10.87; verbatimLongitude: -85.392; verbatimCoordinateSystem: Decimal; decimalLatitude: 10.87; decimalLongitude: -85.392; **Identification:** identifiedBy: AJ Fleming; dateIdentified: 2015; **Event:** samplingProtocol: reared from caterpillar of Parasa wellesca (Limacodidae); verbatimEventDate: 26-Aug-1998; **Record Level:** language: en; institutionCode: CNC; collectionCode: Insects; basisOfRecord: Pinned Specimen
- **Type status:** Other material. **Occurrence:** occurrenceDetails: http://janzen.sas.upenn.edu; catalogNumber: DHJPAR0018556; recordedBy: D.H. Janzen, W. Hallwachs, Gloria Sihezar; individualID: DHJPAR0018556; individualCount: 1; sex: M; lifeStage: adult; preparations: pinned; otherCatalogNumbers: ASTAI1203-07, 97-SRNP-6403; **Taxon:** scientificName: Uramya sibinivora; phylum: Arthropoda; class: Insecta; order: Diptera; family: Tachinidae; genus: Uramya; specificEpithet: sibinivora; scientificNameAuthorship: Guimarães, 1980; **Location:** continent: Central America; country: Costa Rica; countryCode: CR; stateProvince: Alajuela; county: Area de Conservación Guanacaste; locality: Sector San Cristobal; verbatimLocality: Quebrada Cementerio; verbatimElevation: 700; verbatimLatitude: 10.871; verbatimLongitude: -85.387; verbatimCoordinateSystem: Decimal; decimalLatitude: 10.871; decimalLongitude: -85.387; **Identification:** identifiedBy: AJ Fleming; dateIdentified: 2015; **Event:** samplingProtocol: reared from caterpillar of Parasa wellesca (Limacodidae); verbatimEventDate: 17-Aug-1997; **Record Level:** language: en; institutionCode: CNC; collectionCode: Insects; basisOfRecord: Pinned Specimen
- **Type status:** Other material. **Occurrence:** occurrenceDetails: http://janzen.sas.upenn.edu; catalogNumber: DHJPAR0018554; recordedBy: D.H. Janzen, W. Hallwachs, gusaneros; individualID: DHJPAR0018554; individualCount: 1; sex: M; lifeStage: adult; preparations: pinned; otherCatalogNumbers: ASTAI1201-07, 93-SRNP-2132; **Taxon:** scientificName: Uramya sibinivora; phylum: Arthropoda; class: Insecta; order: Diptera; family: Tachinidae; genus: Uramya; specificEpithet: sibinivora; scientificNameAuthorship: Guimarães, 1980; **Location:** continent: Central America; country: Costa Rica; countryCode: CR; stateProvince: Guanacaste; county: Area de Conservación Guanacaste; locality: Sector Santa Rosa; verbatimLocality: Cafetal; verbatimElevation: 280; verbatimLatitude: 10.858; verbatimLongitude: -85.611; verbatimCoordinateSystem: Decimal; decimalLatitude: 10.858; decimalLongitude: -85.611; **Identification:** identifiedBy: AJ Fleming; dateIdentified: 2015; **Event:** samplingProtocol: reared from caterpillar of Parasa wellesca (Limacodidae); verbatimEventDate: 29-Jun-1993; **Record Level:** language: en; institutionCode: CNC; collectionCode: Insects; basisOfRecord: Pinned Specimen
- **Type status:** Other material. **Occurrence:** occurrenceDetails: http://janzen.sas.upenn.edu; catalogNumber: DHJPAR0018552; recordedBy: D.H. Janzen, W. Hallwachs, Petrona Rios; individualID: DHJPAR0018552; individualCount: 1; sex: M; lifeStage: adult; preparations: pinned; otherCatalogNumbers: ASTAI1199-07, 04-SRNP-30224; **Taxon:** scientificName: Uramya sibinivora; phylum: Arthropoda; class: Insecta; order: Diptera; family: Tachinidae; genus: Uramya; specificEpithet: sibinivora; scientificNameAuthorship: Guimarães, 1980; **Location:** continent: Central America; country: Costa Rica; countryCode: CR; stateProvince: Guanacaste; county: Area de Conservación Guanacaste; locality: Sector Pitilla; verbatimLocality: Loaiciga; verbatimElevation: 445; verbatimLatitude: 11.02; verbatimLongitude: -85.413; verbatimCoordinateSystem: Decimal; decimalLatitude: 11.02; decimalLongitude: -85.413; **Identification:** identifiedBy: AJ Fleming; dateIdentified: 2015; **Event:** samplingProtocol: reared from caterpillar of Euclea mesoamericana (Limacodidae); verbatimEventDate: 16-Feb-2004; **Record Level:** language: en; institutionCode: CNC; collectionCode: Insects; basisOfRecord: Pinned Specimen

#### Description

**Male** (Fig. 18): 8–15 mm. **Head** (Fig. 18b): antenna: first flagellomere dark brown on lower 2/3, turning to a slightly lighter brownish-orange on upper third and adjacent to pedicel; pedicel dark orange; arista 1.5X as long as first flagellomere, dark brown and minutely pubescent; palpus yellow, and haired; fronto-orbital plate, parafacial and gena silver pollinose. **Thorax** (Fig. 18a, c): entirely gray pollinose; dorsum of thorax, sternopleura, hypopleura, and pteropleura yellow-white pilose; 3 postsutural supra-alar bristles; 2 katepisternal bristles; postpronotum and anepisternum bearing fine, yellow-white hairs; underside of scutellum bearing a tuft of white hairs near basal marginal bristle. Legs: fore femur gray pollinose; mid and hind femora dark brown to black in ground color, contrasting with the dark orange tibiae; fore femur with fine white hairs. Wings: smoky gray, with a slight, smoky amber infuscation delineating each vein. **Abdomen** (Fig. 18a): ventral surface of abdomen yellow-white pollinose; median marginal bristles on all abdominal tergites (ST1+2, T3, T4, and T5); 2 pairs of median discal bristles on T3, anteriormost pair 1/2 the diameter of posteriormost pair; 1 pair of median discal bristles on T4 and T5; ground color of abdomen orange-brown up to tergite 5, where it darkens to brown-black; silver pollinosity covering anteriormost half of T3, T4 and T5; ST1+2 with lateral gray pollinose spots on posterior half. **Terminalia** (Fig. 18d, e, f): sternite 5 with two small lobes, inner margin covered in dense pollinosity appearing darker than surrounding cuticle, apical edges of lobe of sternite 5 bearing short, stout bristles interspersed with longer bristles close to apical margin; sternite 5 with a wide V-shaped median cleft, 0.46X length of sternite from lobe apex to base; cercus sharply pointed and distinctly tapered; apical section 1.7X as long as upper lobe; surstylus equilaterally oblong and scythe-like in lateral view; posterodorsal half of surstylus haired, apex with few short bristles but otherwise almost bare; tips of surstyli with a slight lobe when viewed dorsally; strongly angled inwards; 1.4X as long as cercus.

**Female**: no female specimen was available to us for study.

#### Diagnosis

*Uramya sibinivora* can be distinguished from all other Neotropical species of *Uramya* by the following combination of traits: conspicuous white pilosity covering the katepisternum, meron, and anepimeron, ventral surface of abdomen, dorsum of the thorax, scutellum white pilose, only one pair of median discal bristles on T3, and the shape of the surstylus.

#### Distribution

Costa Rica: ACG (Provs. Alajuela and Guanacaste), 280–1,080 m; Paraguay: Guairá, Villarica; Venezuela: Edo. Nueva Esparta, El Valle.

#### Ecology

Within the context of the ACG inventory, *Uramya sibinivora* has been reared 6 times from *Parasa wellesca* Dyar and *Euclea mesoamericana* Corrales & Epstein (Limacodidae).

## Identification Keys

### Revised key to the males of *Uramya* Robineau-Desvoidy of Central and South America

**Table:** 

1	Thorax either yellow-white or black pilose dorsally; katepisternum, meron, anepimeron, and ventral surface of abdomen yellow-white pilose	2
–	Thorax black pilose dorsally; katepisternum, meron and anepimeron black pilose; ventral surface of abdomen black pilose	11
2	Dorsum of thorax and scutellum yellow-white pilose, matching sides and ventral surface of abdomen; postpronotum and anepisternum with fine, yellow-white hairs	3
–	Dorsum of thorax and scutellum black pilose, contrasting with white pilose sides of thorax and ventral surface of abdomen	5
3	2 pairs of median discals on T3, and a row of median marginals on T4; surstyli slender	***U. plaumanni*** Guimarães
–	Only 1 pair of median discals on T3 and T4; surstyli falciform	4
4	Abdomen cylindrical and elongate, narrower than thorax at basein dorsal view; surstyli long and scythe-like; 2 katepisternal bristles; row of marginal bristles on T3 and T4	***U. sibinivora*** Guimarães
–	Abdomen subtriangular, at base in dorsal view; surstyli uniformly wide, tapering to a pointed tip; 3 katepisternal bristles; 1 pair of marginal bristles on T3 and T4	***U. insolita*** Guimarães
5	First postusutural supra-alar bristle absent; median marginal bristles only on ST1+2; two pairs of median discals on T3 and T4	***U. pannosa* sp. nov.**
–	Three postsutural supra-alar bristles; median marginal bristles present on all tergites; one or two pairs of discal bristles on T3 and T4	6
6	Pedicel orange	7
–	Pedicel dark brown	9
7	One pair of median marginals on ST1+2, T3 and T4; femora of dark ground color, covered in gray pollinosity; tibiae of yellow or dark brown ground color	***U. penicillata* sp. nov.**
–	One pair of median marginals on ST1+2, an incomplete row of marginal bristles on T3, T4 and T5; legs of yellow ground color, covered in black hairs; coxae orange	8
8	Sides of thorax and underside of abdomen densely pilose; legs conspicuously yellow, thinly dark haired; T3 and T4 densely covered in erect hairs more than half as long as discal bristles; T5 lacking row of discal bristles.	***U. setiventris*** (Wulp)
–	Sides of thorax and underside of abdomen moderately pilose; legs densely covered in dark hair, making them appear brown; T3 and T4 densely covered in appressed hairs not more than 1/3 as long as discal bristles; row of discal bristles present on T5.	***U. nitida* sp. nov.**
9	3 katepisternal bristles	***U. infracta* sp. nov.**
–	2 katepisternal bristles	10
10	ST1+2 with gray pollinosity dorsolaterally; underside of scutellum with a tuft of black hairs near basal marginal bristle; ST1+2 with 2 strong median marginal bristles and a row of weaker marginal bristles, extending to lateral marginal bristles; T3 and T4 with a row of marginal bristles	***U. constricta* sp. nov.**
–	Abdominal ST1+2 lacking gray pollinosity dorsolaterally; only one pair of median marginals on each of ST1+2 and T3, and T4 with a row of marginal bristles; underside of scutellum with a tuft of white hairs near basal marginal bristle	***U. albosetulosa* sp. nov.**
11	Wings yellow along base, brown on outer costal portion; calypters strongly infuscate	12
–	Wings brownish costobasally; calypters pale yellow to white, not strongly infuscate	18
12	ST1+2, T3, T4, and T5 segments with conspicuous patches of white pollinosity dorsolaterally	***U. octomaculata*** (Townsend)
–	At most 3 of the 5 visible abdominal segments with conspicuous patches of white pollinosity dorsolaterally or abdomen devoid of white pollinosity.	13
13	Abdomen completely devoid of white pollinosity	***U. umbratilis*** (Reinhard)
–	Abdomen with patches of white pollinosity	14
14	Abdomen with tergal banding broken into distinct, white pollinose patches on ST1+2, T3 and T4; T5 testaceous pollinose	***U. quadrimaculata*** (Macquart)
–	Abdomen with tergal banding broken into distinct, white pollinose patches on T3–T5, ST1+2 bare.	15
15	Thorax with greenish-gold pollinosity	***U. penai*** (Guimarães)
–	Thorax with grayish-white pollinosity	16
16	Wing with strongly contrasting colors: yellow along basal 1/3 and brown on outer 2/3 of costal portion; abdomen reddish-black with white pollinosity along sides of tergites	***U. indita*** (Walker)
–	Wing coloration not strongly contrasting; abdomen not distinctly reddish black, lacking white pollinosity on sides of tergites	17
17	Abdominal T5 sharply pointed; thorax with 3 katepisternal bristles and 3 postsutural dorsocentrals; wing coloration overall very faint, pale yellow at base and with costal portion faintly brown	***U. acuminata*** (Wulp)
–	Abdominal T5 broadly rounded; thorax with 2 katepisternal bristles and 4 postsutural dorsocentrals; wing bright yellow at base, with brown costal region	***U. venusta*** (Wulp)
18	Abdominal T3 and T4 with a continuous transverse band of white pollen on anterior 1/4, fading towards apex; T5 not pollinose	***U. nitens*** (Schiner)
–	Abdominal T3 and T4 with thick silver pollen on sides, broken by a longitudinal dorsocentral stripe, present on basal 2/3 or less; T5 pollinose	19
19	T5 uniformly brown pollinose	***U. townsendi*** Guimarães
–	T5 brownish pollinose from middle to apex, white pollinose on sides	20
20	Scutellum uniformly white pollinose, bearing at least two strong lateral bristles and at least two pairs of discal bristles	21
–	Scutellum brownish pollinose on anterior half, with two strong lateral bristles and discal bristles weak to absent (if discals are present, they are almost indistinct from surrrounding hairs)	22
21	Two strong, distinct discal scutellar bristles; anatergite bare	***U. brevicauda*** (Curran)
–	Discal scutellar bristles almost indistinct, appearing as thickened scutellar hairs; anatergite with a small patch of black hairs	***U. lativittata*** **sp. nov.**
22	Abdominal T4 bearing a distinct ventrolateral tuft of hairs.	***U. fasciata*** (Macquart)
–	Abdominal T4 with hairs uniform in length.	23
23	Abdomen cylindrical; T5 elongated into a long tail-like process, at least 2–3X as long as T4; T3 with a pair of strong, median discal bristles; arista long and pubescent on basal half	***U. producta*** Robineau-Desvoidy
–	Abdomen more or less flattened dorsoventrally; T5 not produced into a long, tail-like process; if pointed, then less than 2–3X as long as T4; arista bare to minutely pubescent on basal half	24
24	Ventral surface of abdomen yellow-white pilose	***U. longa*** (Walker)
–	Ventral surface of abdomen black pilose	25
25	Abdominal ST1+2 bearing silver pollinose spots, lacking silver pollinosity along posterior margin; wing veins strongly infuscate, giving a dark grey tonality to the leading edge of the wing.	***U. contraria* sp. nov.**
–	Abdominal ST1+2 lacking silver pollinose spots, but with a thin transverse band of silver pollinosity along posterior margin; wing veins not strongly infuscate, infuscation only visible on basal halves of R_4+5_ and M.	***U. lunula* sp. nov.**

This key builds on the work accomplished by Guimarães (1980). As observed by Guimarães (1980), females show less morphological differences between species. As a result, our key is based on male morphological characters only. For female character states, see individual species descriptions.

## Analysis

**Figure 19.**
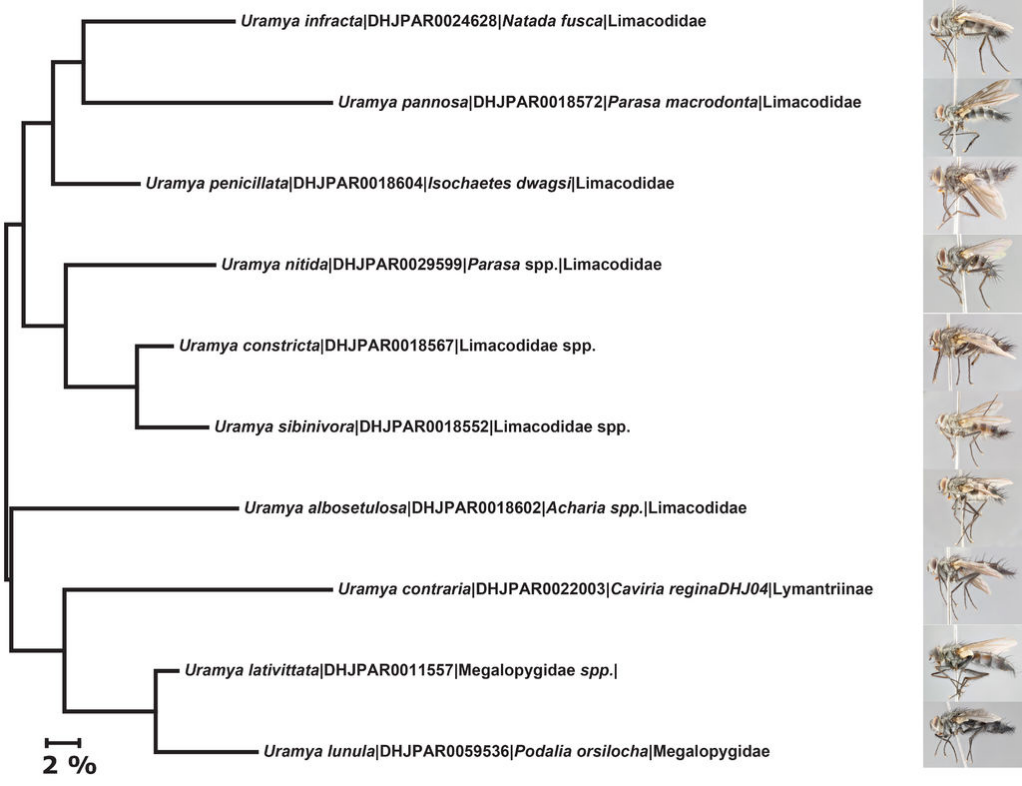
Neighbor Joining tree (NJ – Saitou and Nei 1987) comparing the species of *Uramya* present in ACG. Tree based on the Kimura-2-parameter (Kimura 1980) made using MEGA6 (Tamura et al. 2013) for a single specimen from each of the *Uramya* species discussed here. Tip labels include: species name|sample accession|species parasitized|family of parasitized caterpillar, and the image of a male in lateral view. Each sample presented here is the holotype for the species described here, except *U. sibinivora.* This phenogram shows the low intra-specific and high inter-specific variation of CO1 barcode sequences.

Fig. 19 is a Neighbor Joining tree (NJ) (Saitou and Nei 1987) for the *Uramya* holotypes reared and DNA-barcoded by this inventory to date. The DNA barcode sequences obtained from the ten species of ACG *Uramya* displayed the strong AT bias characteristic of insect mitochondrial DNA (mean percent GC content 30.33%, SE 0.1) and displayed no insertions or deletions. Within-species variation was low compared to between-species variation. All values of DNA barcode variation were calculated within BOLD and can be re-calculated in the future as more specimens or species are added to the DNA library.

## Supplementary Material

XML Treatment for Uramya

XML Treatment for Uramya albosetulosa

XML Treatment for Uramya constricta

XML Treatment for Uramya contraria

XML Treatment for Uramya infracta

XML Treatment for Uramya lativittata

XML Treatment for Uramya lunula

XML Treatment for Uramya nitida

XML Treatment for Uramya pannosa

XML Treatment for Uramya penicillata

XML Treatment for Uramya sibinivora

## Figures and Tables

**Figure 1a.**
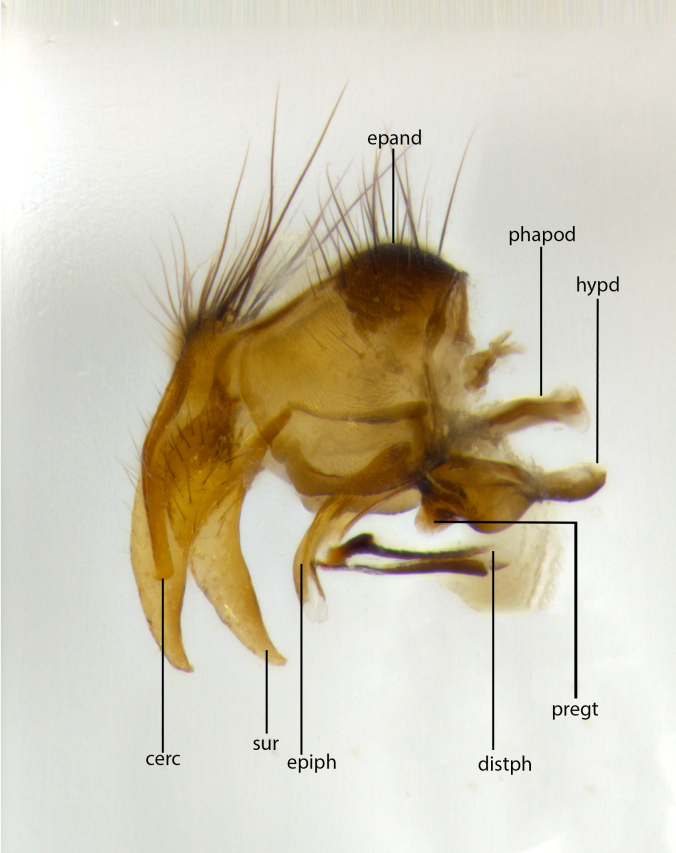
Abbreviations: cerc = cercus; distph = distiphallus; epand = epandrium; epiph = epiphallus; hypd = hypandrium; phapod = phalloapodeme; pregt = pregonite; sur = surstylus.

**Figure 1b.**
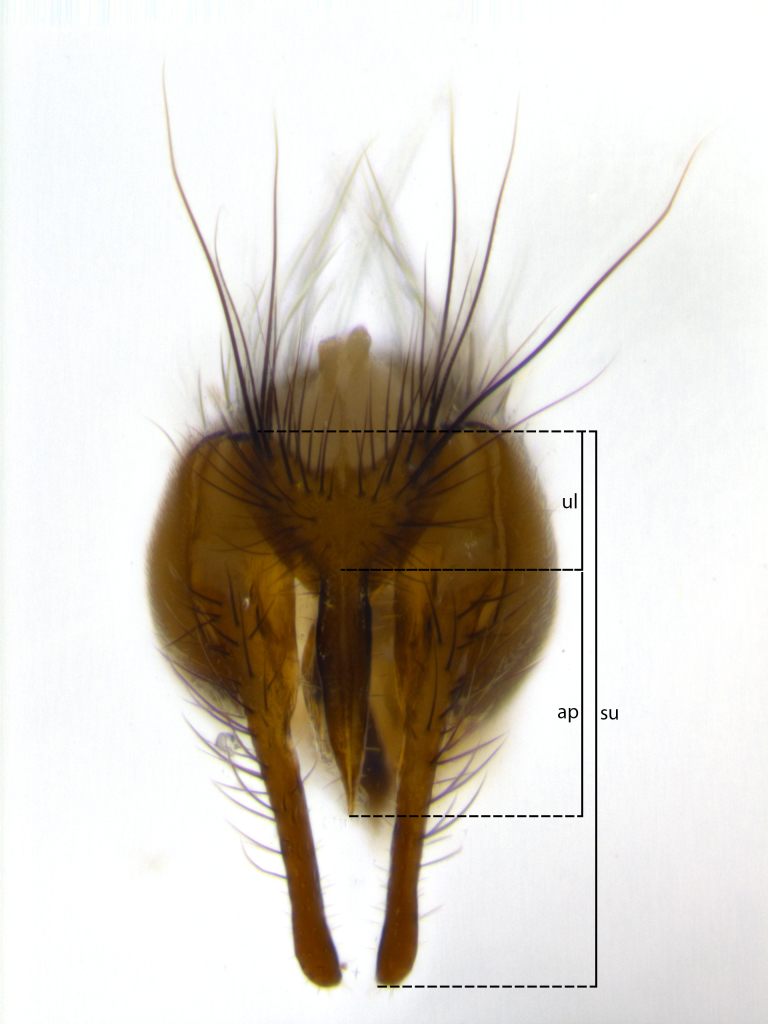
Abbreviations for sections measured: ap = apical section of cercus; ul = upper lobe of cercus; sur = surstylus.

**Figure 3a.**
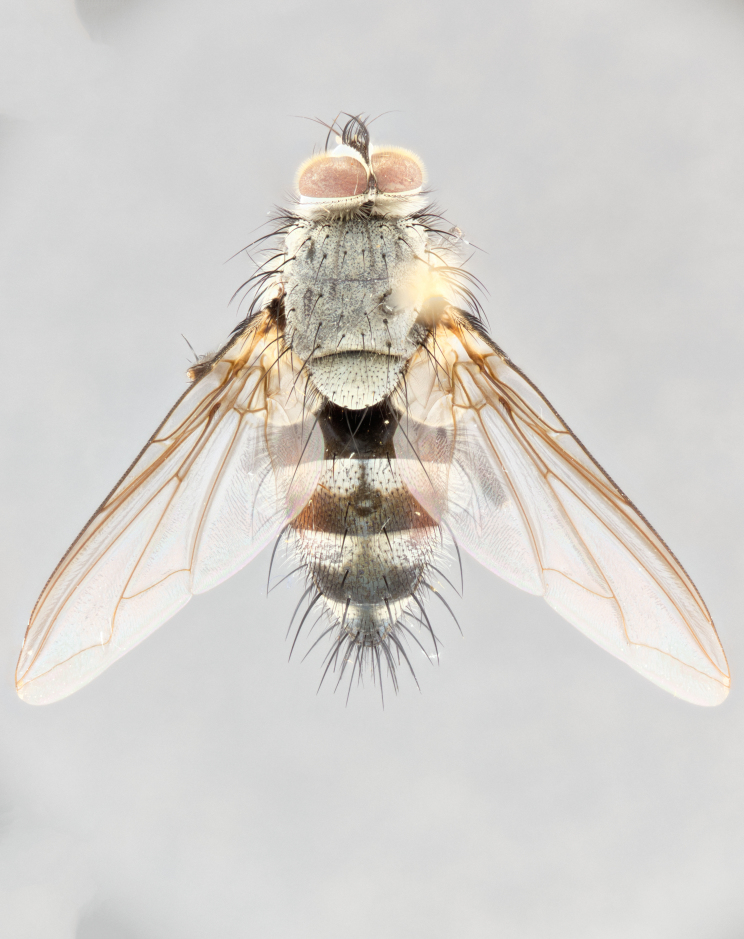
habitus in dorsal view

**Figure 3b.**
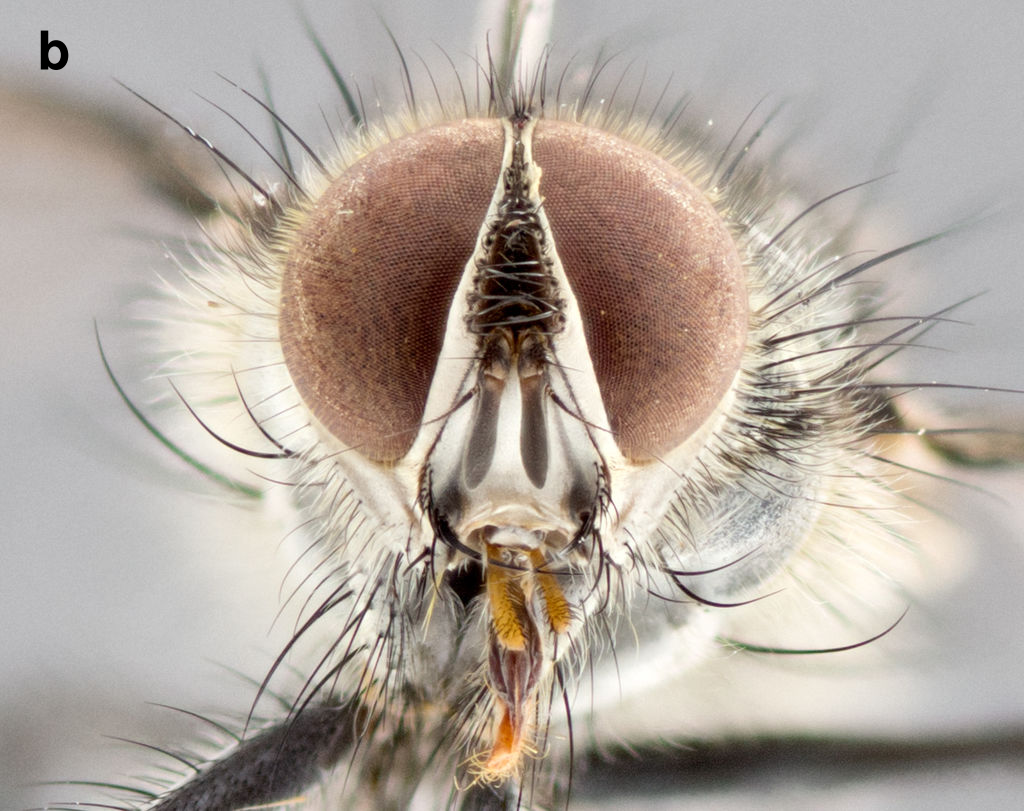
head in frontal view

**Figure 3c.**
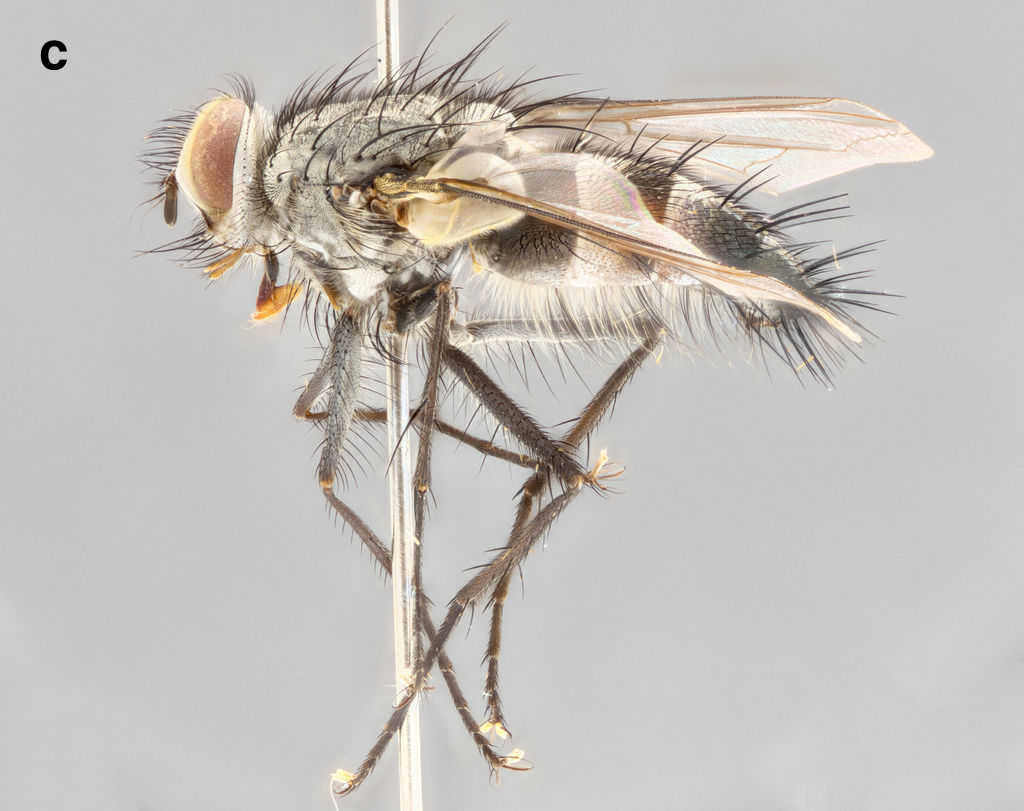
habitus in lateral view

**Figure 3d.**
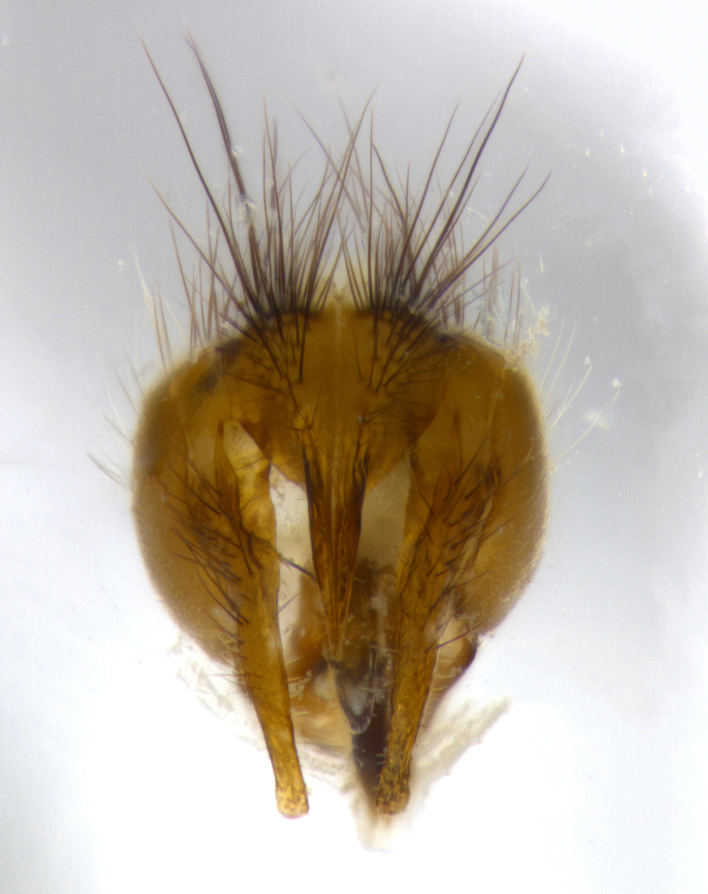
terminalia in dorsal view

**Figure 3e.**
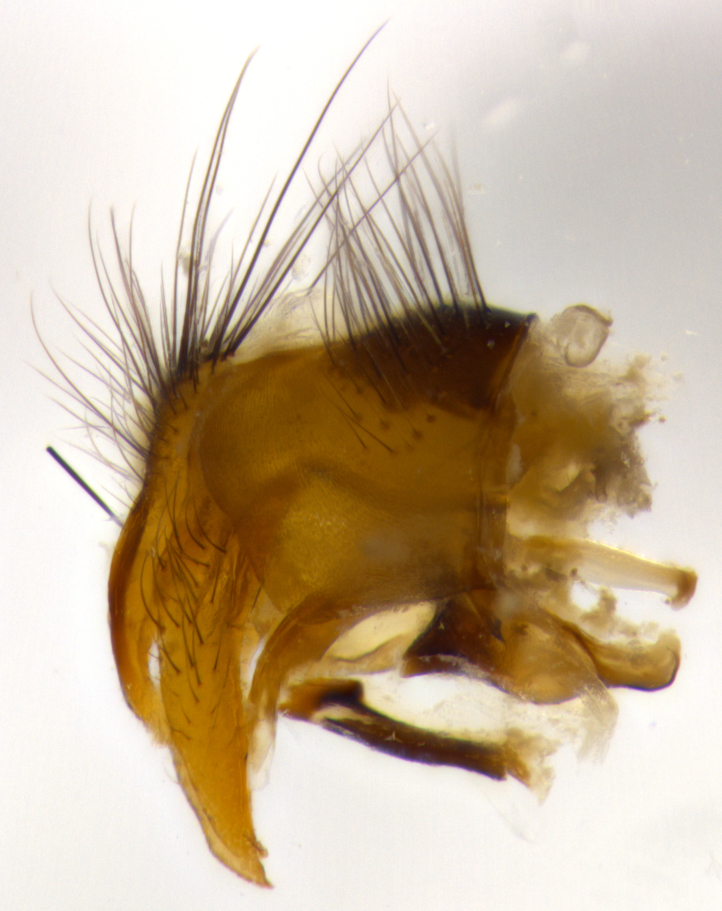
terminalia in lateral view

**Figure 3f.**
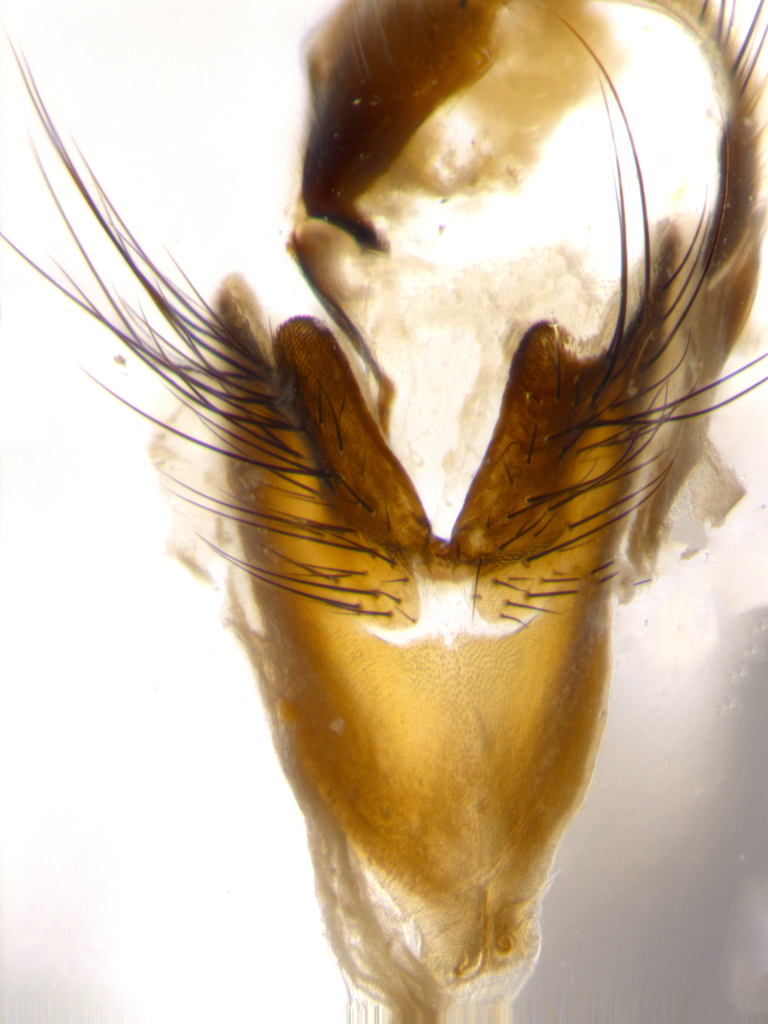
sternite 5 in ventral view

**Figure 4a.**
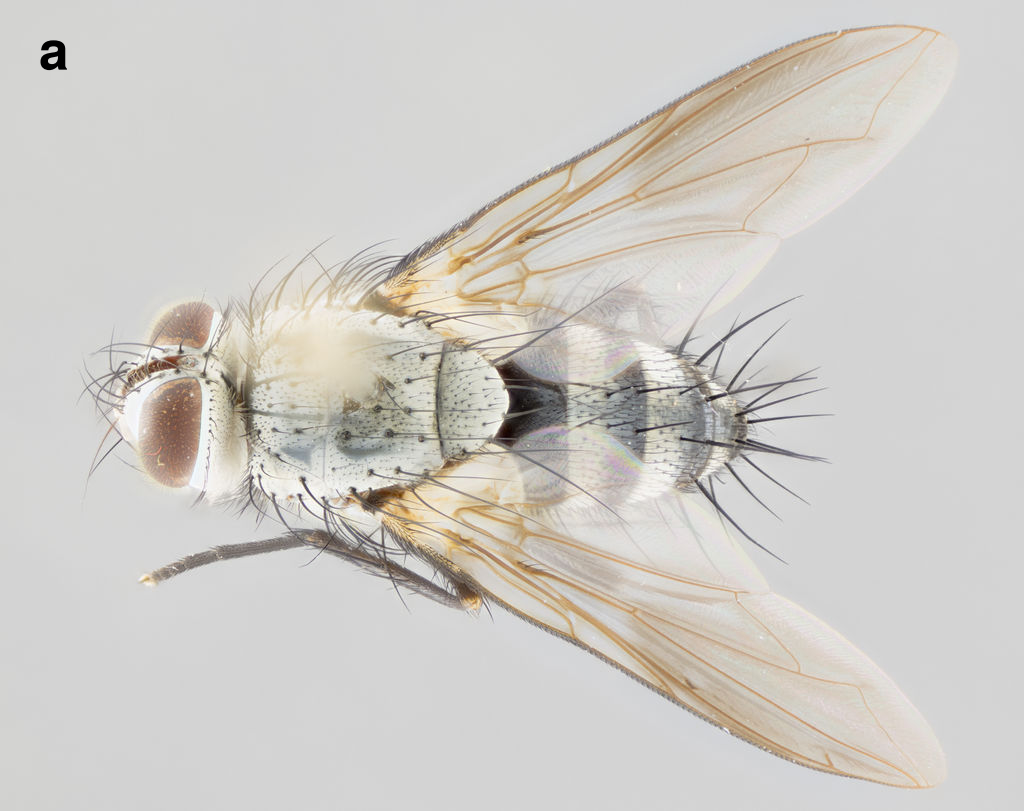
habitus in dorsal view

**Figure 4b.**
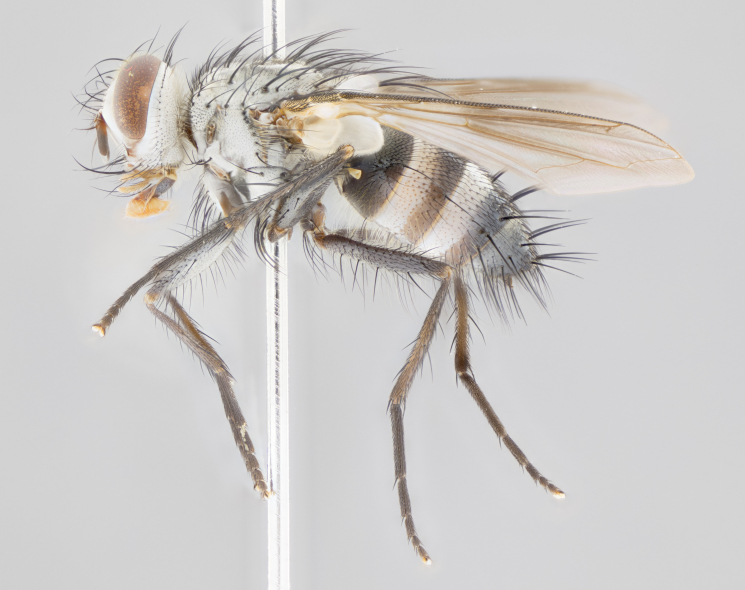
habitus in lateral view

**Figure 5a.**
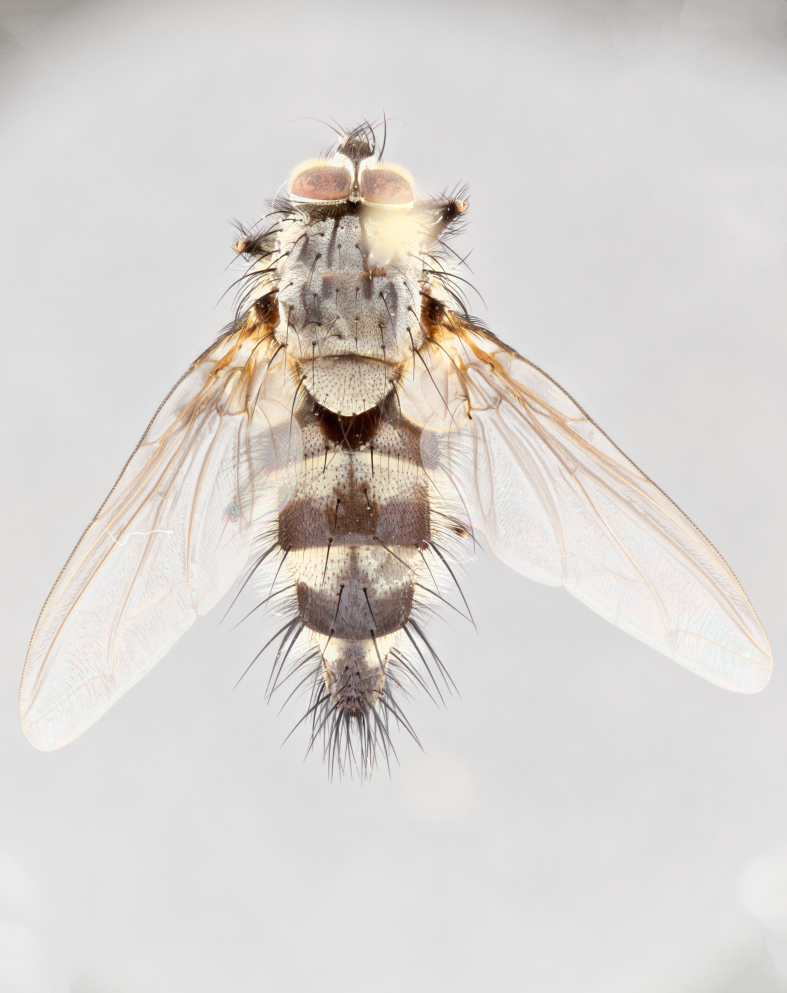
habitus in dorsal view

**Figure 5b.**
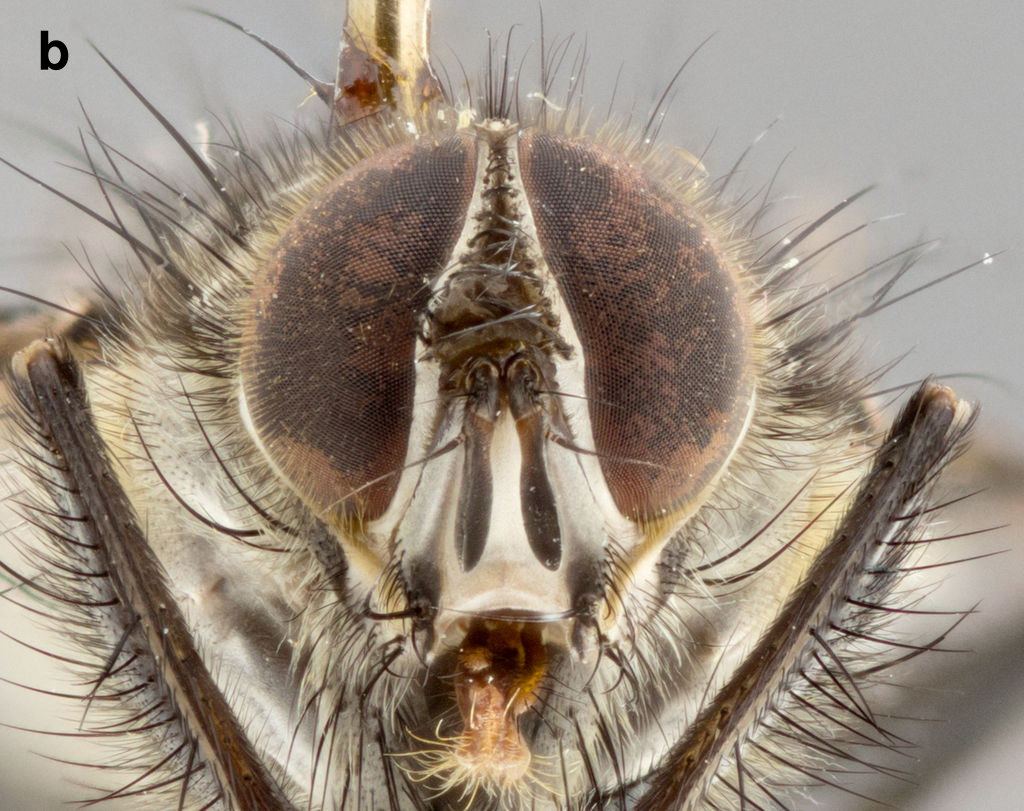
head in frontal view

**Figure 5c.**
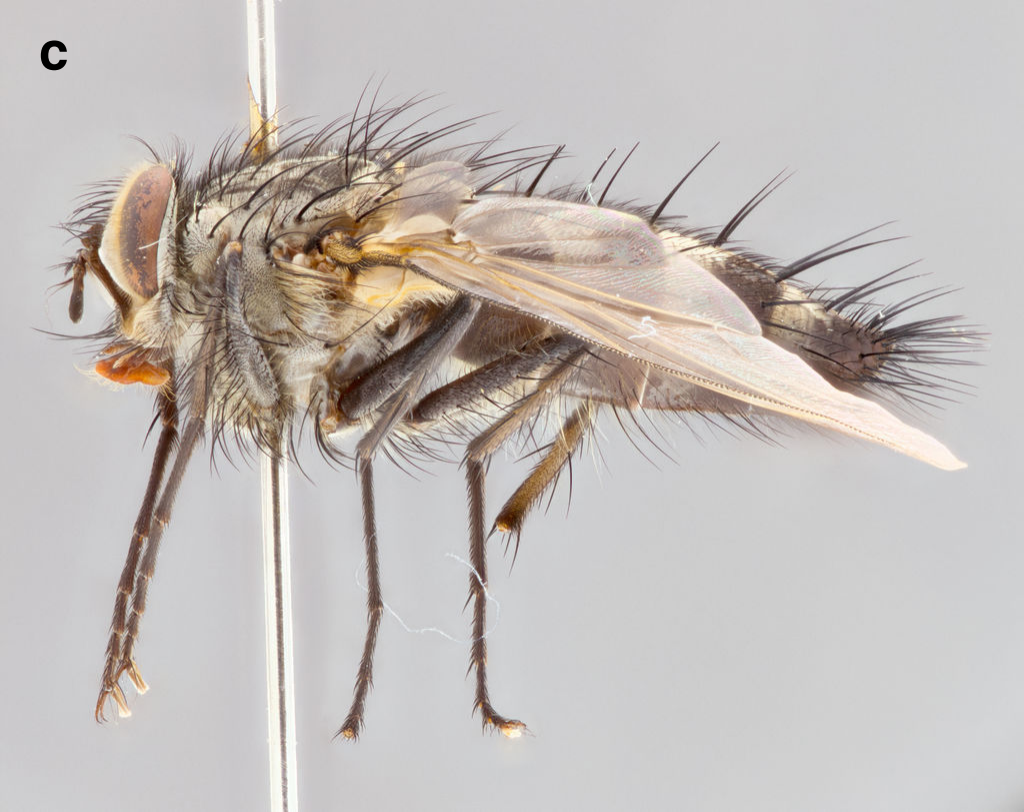
habitus in lateral view

**Figure 5d.**
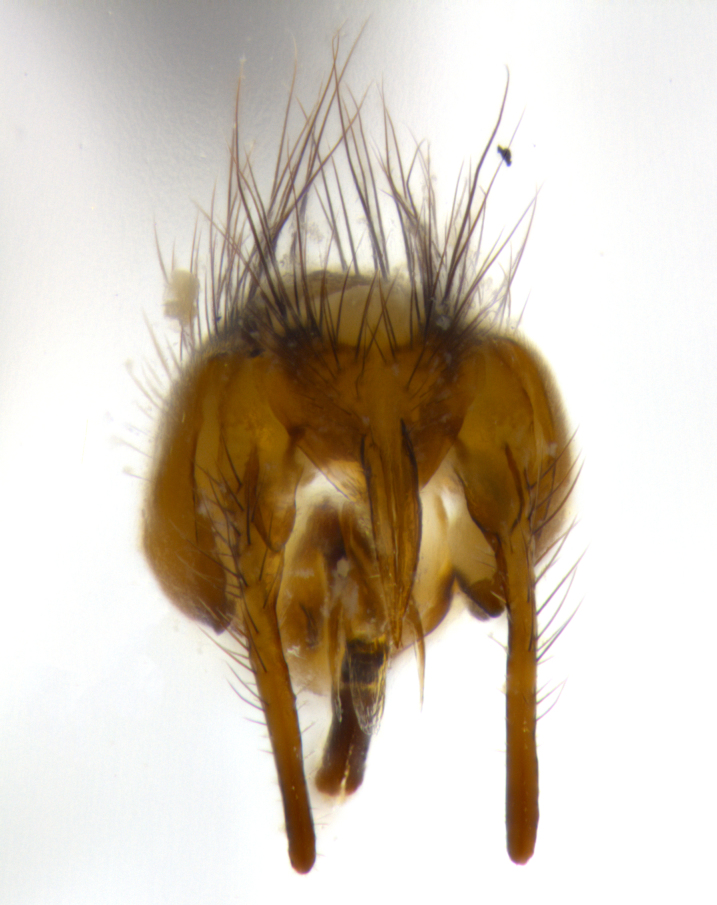
terminalia in dorsal view

**Figure 5e.**
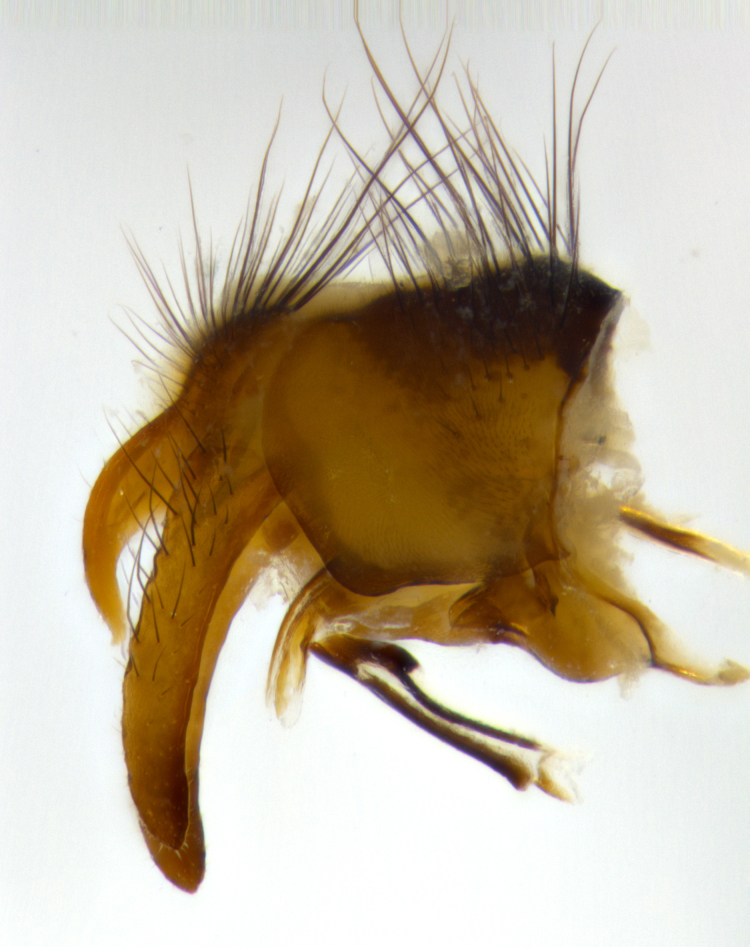
terminalia in lateral view

**Figure 5f.**
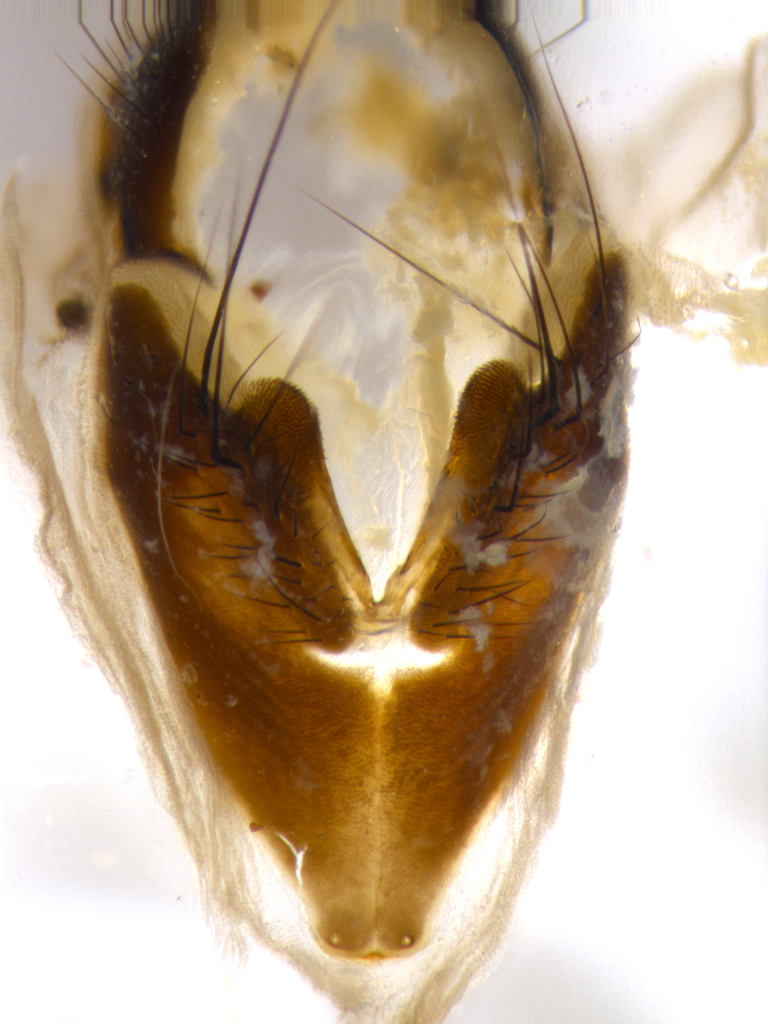
sternite 5 in ventral view

**Figure 6a.**
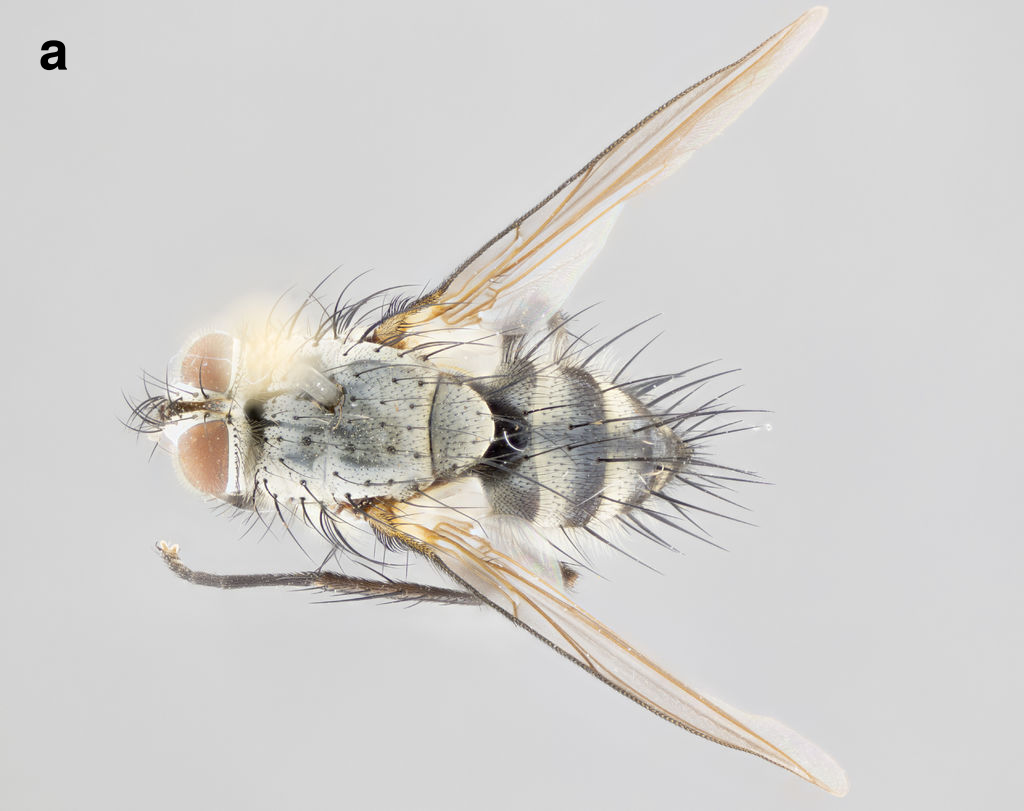
habitus in dorsal view

**Figure 6b.**
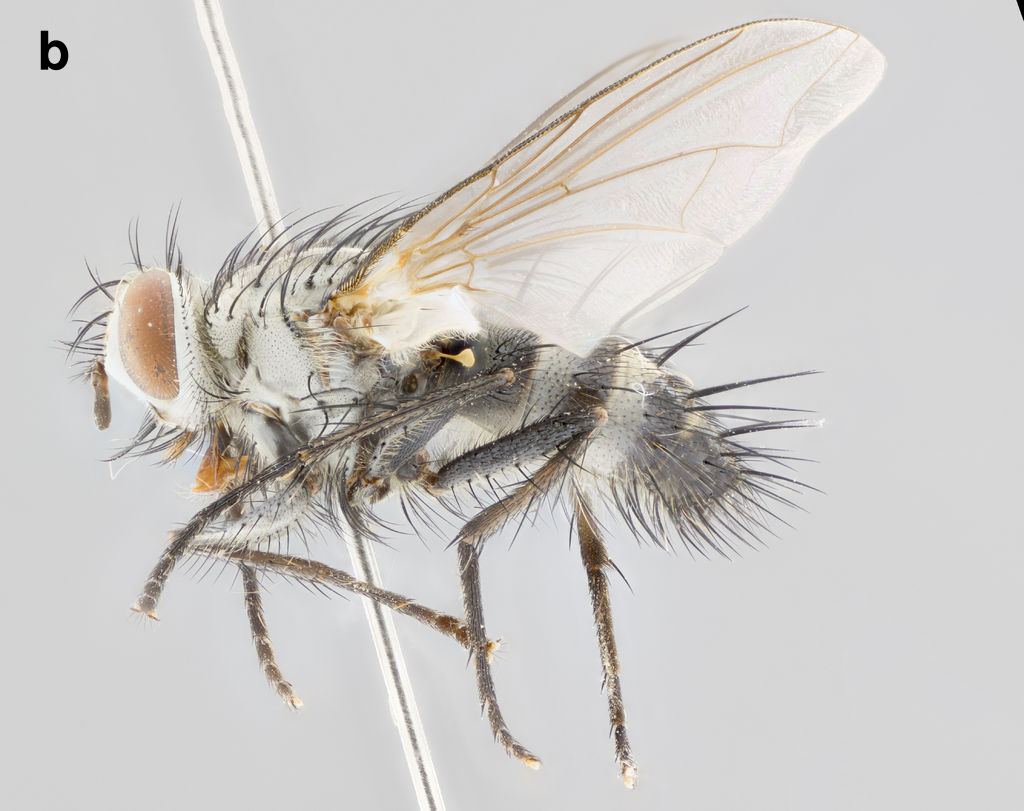
habitus in lateral view

**Figure 7a.**
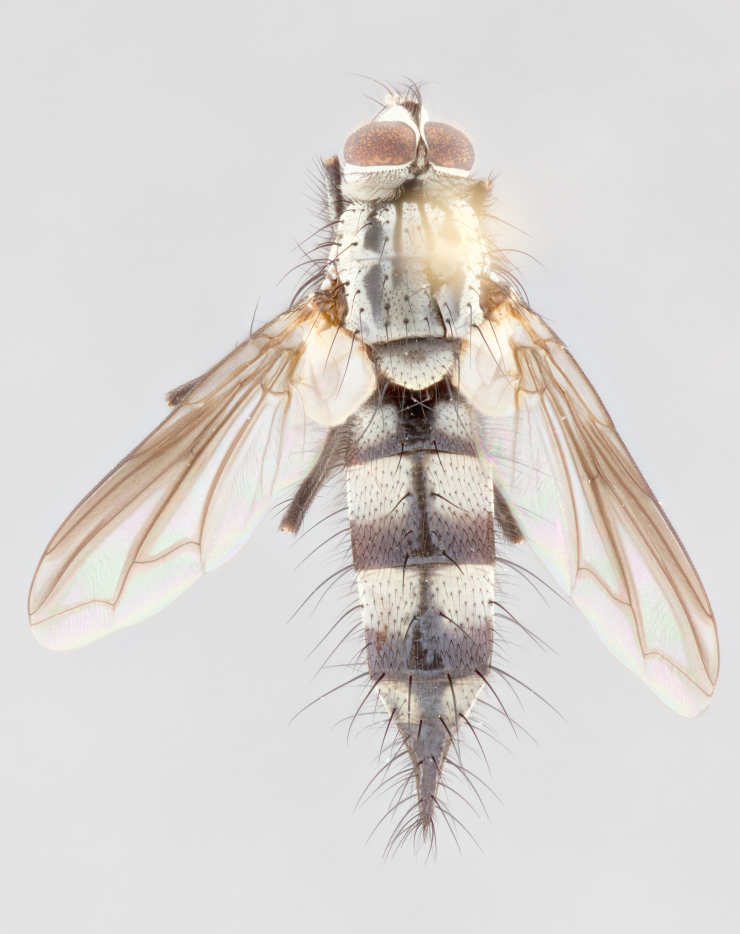
habitus in dorsal view

**Figure 7b.**
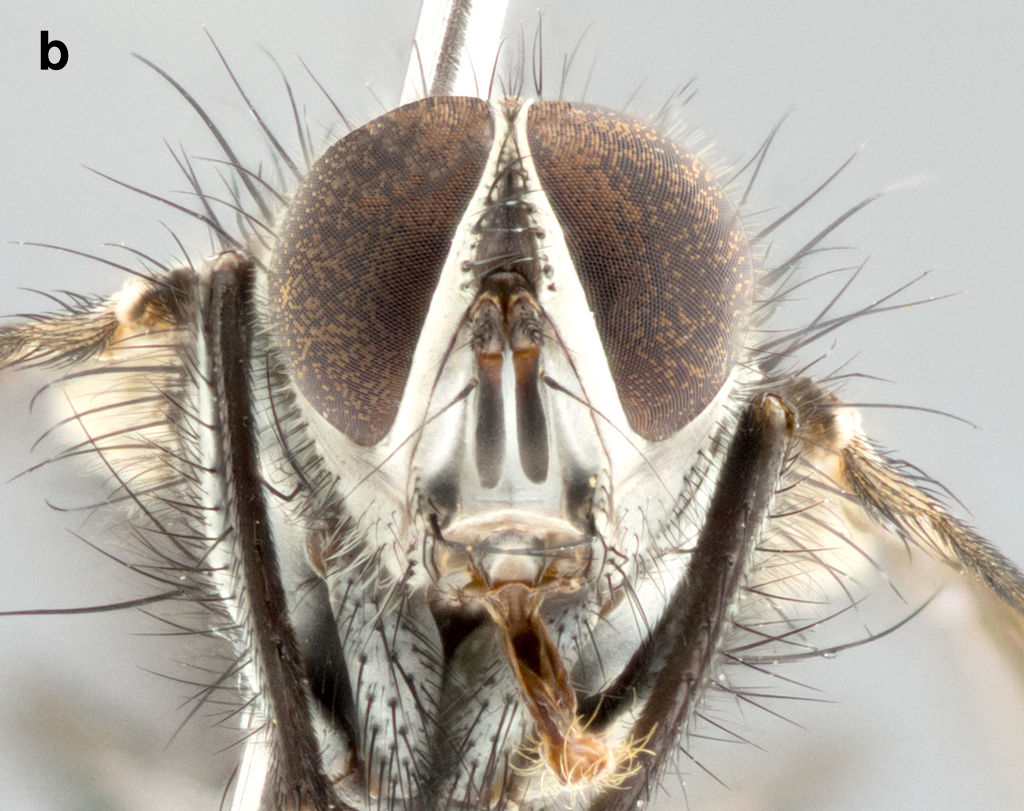
head in frontal view

**Figure 7c.**
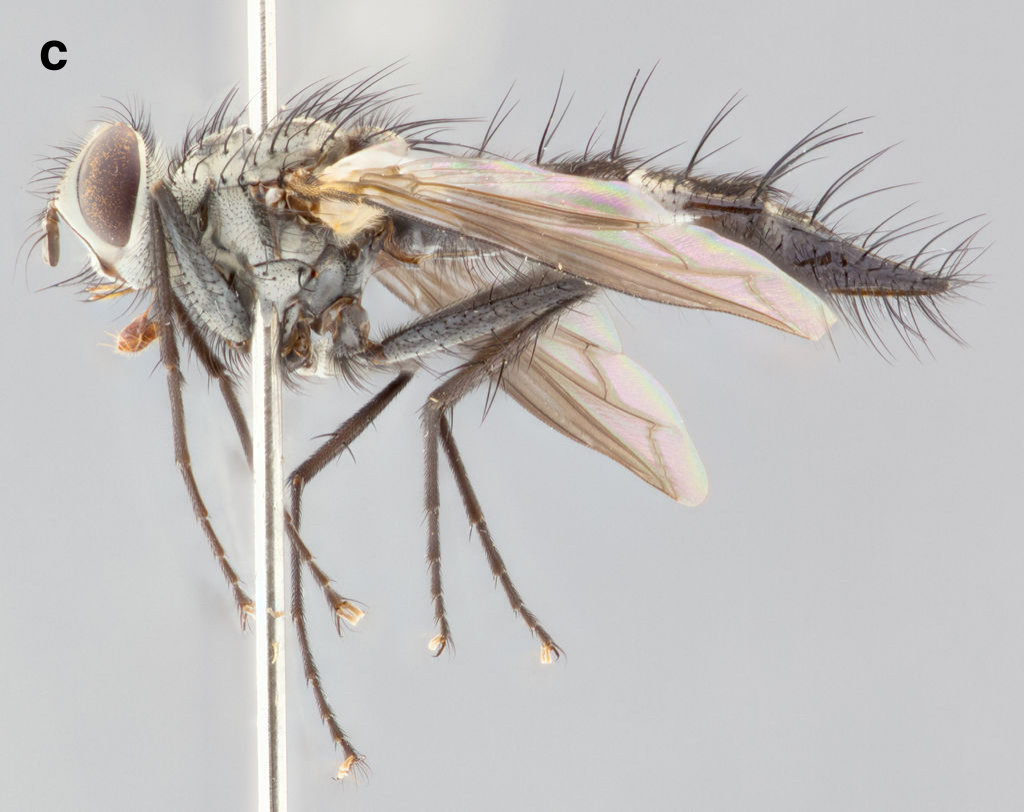
habitus in lateral view

**Figure 7d.**
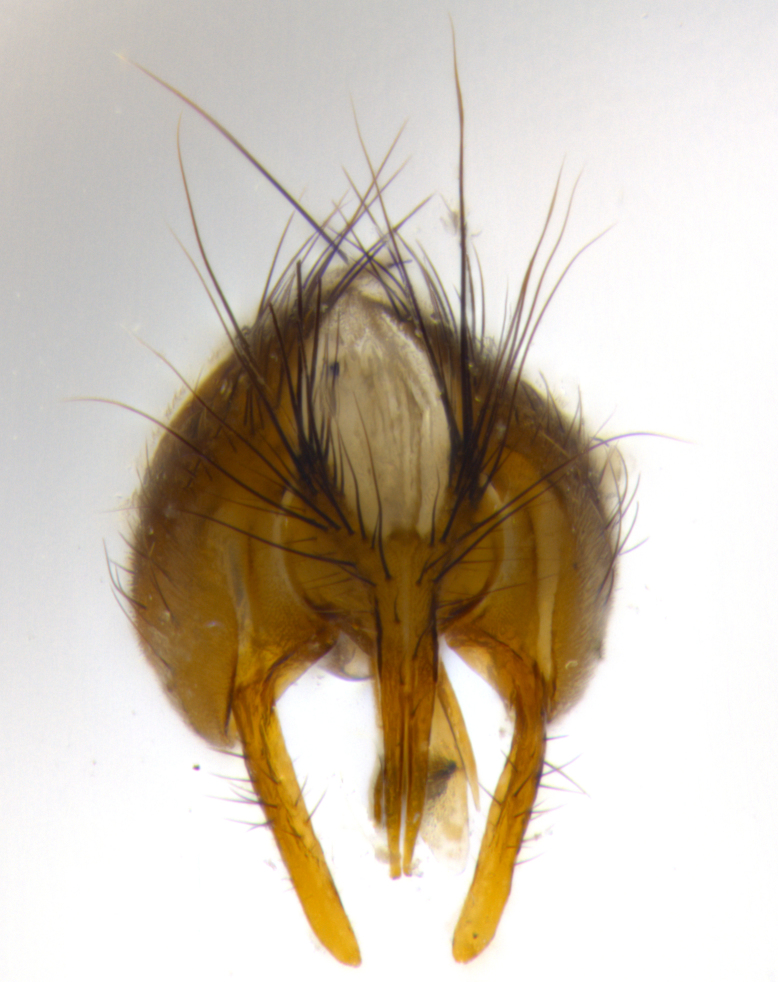
terminalia in dorsal view

**Figure 7e.**
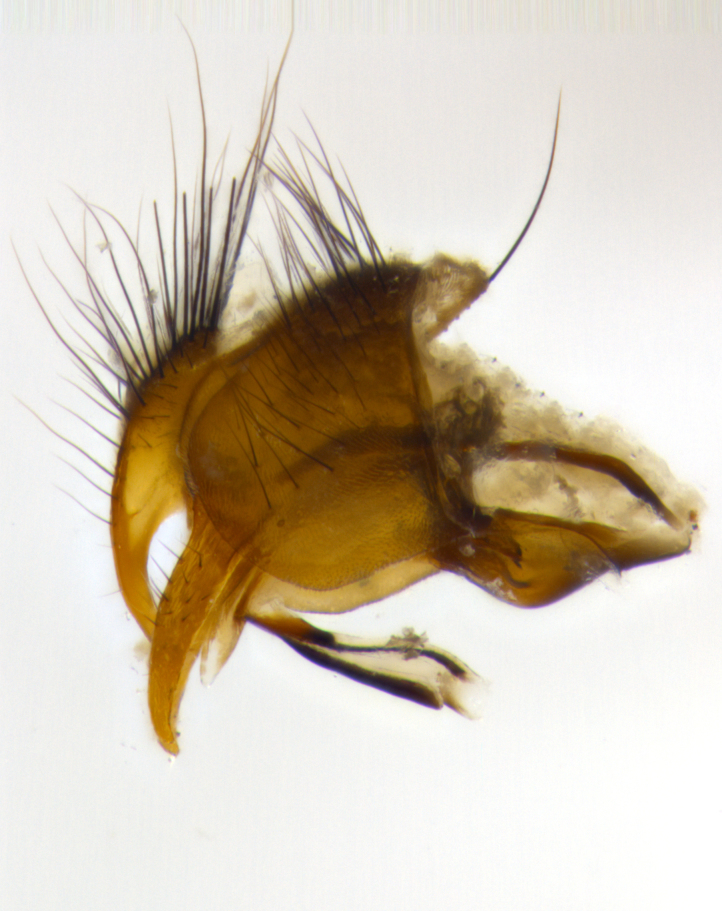
terminalia in lateral view

**Figure 7f.**
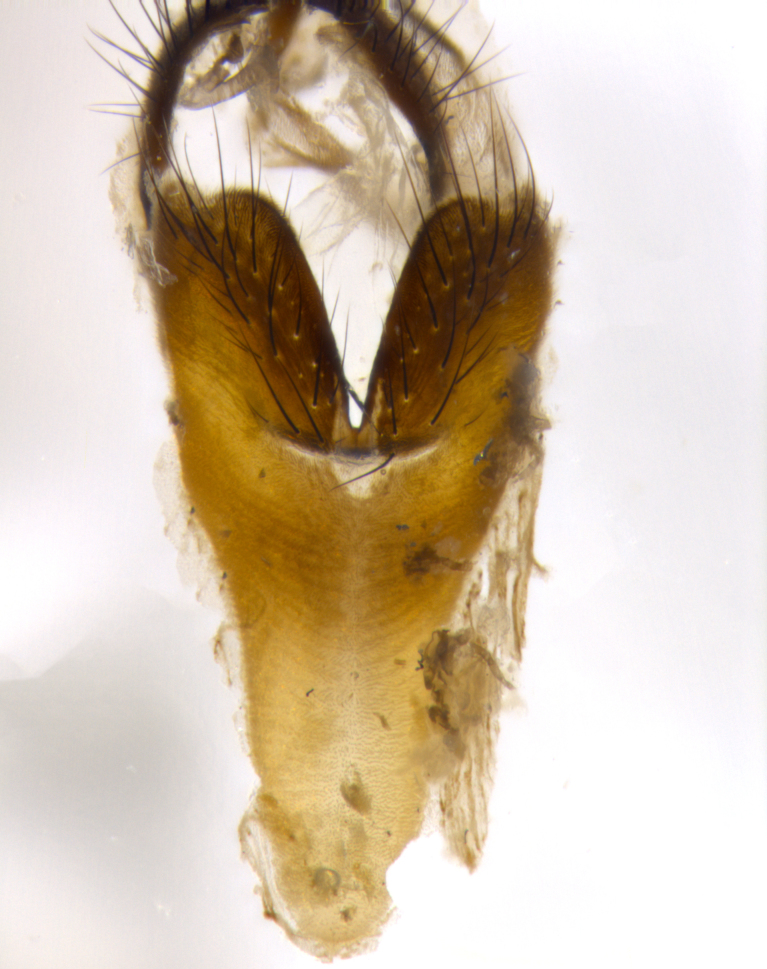
sternite 5 in ventral view

**Figure 8a.**
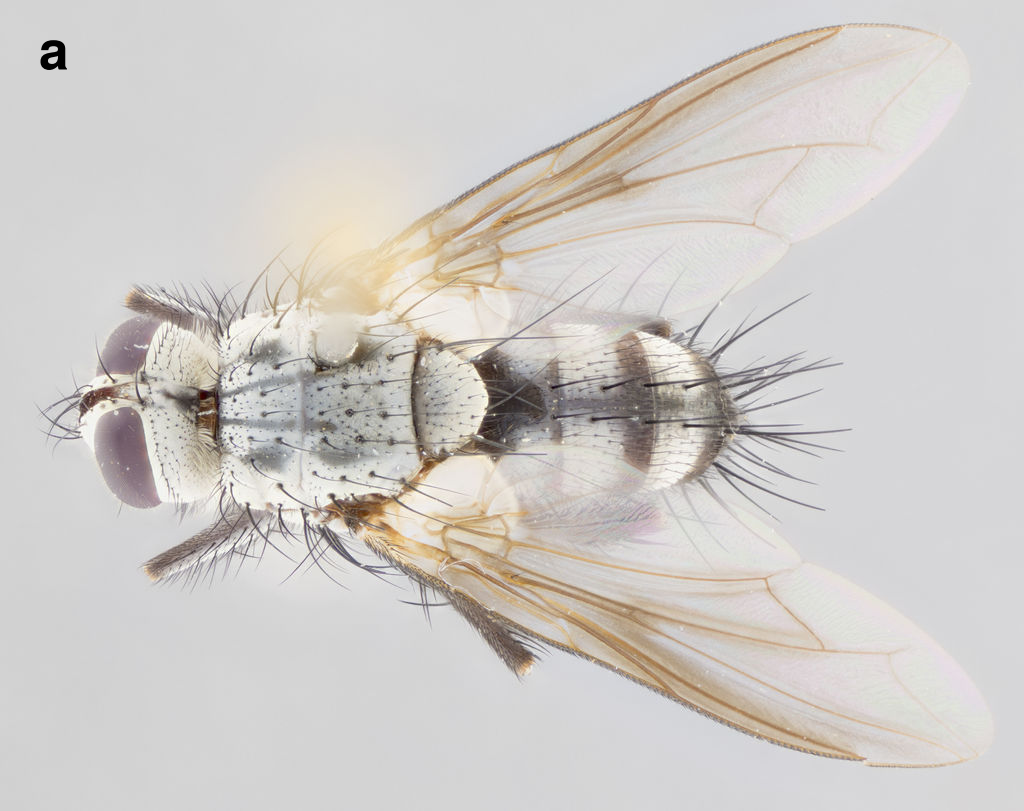
habitus in dorsal view

**Figure 8b.**
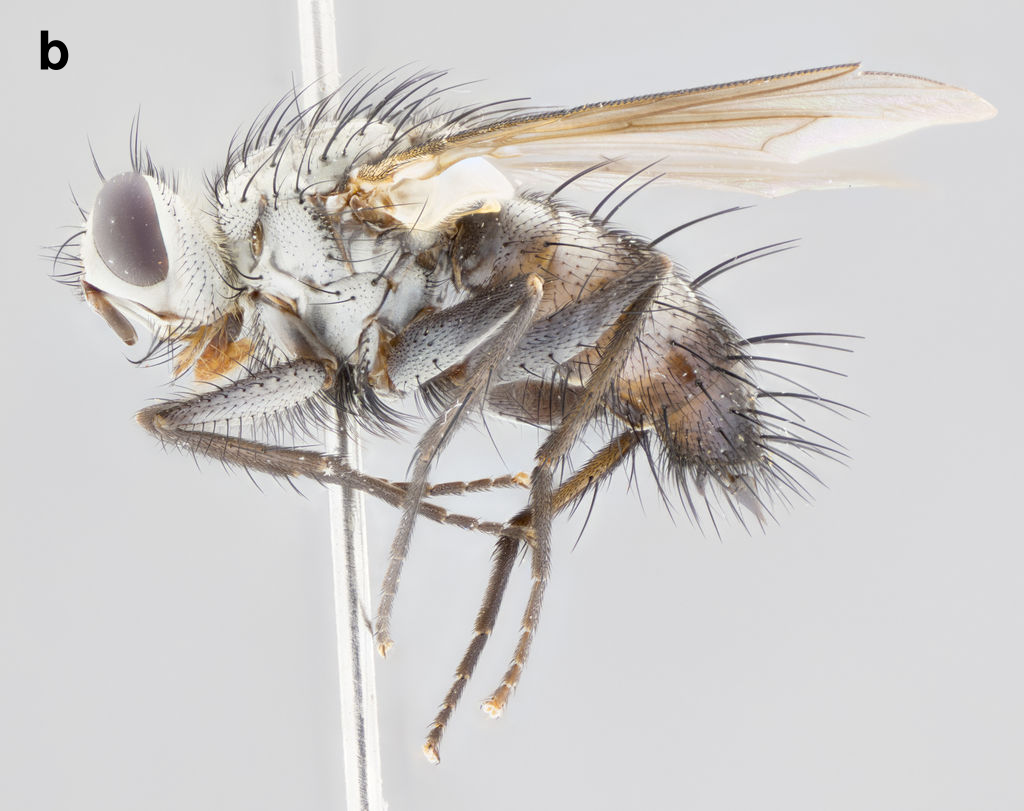
habitus in lateral view

**Figure 9a.**
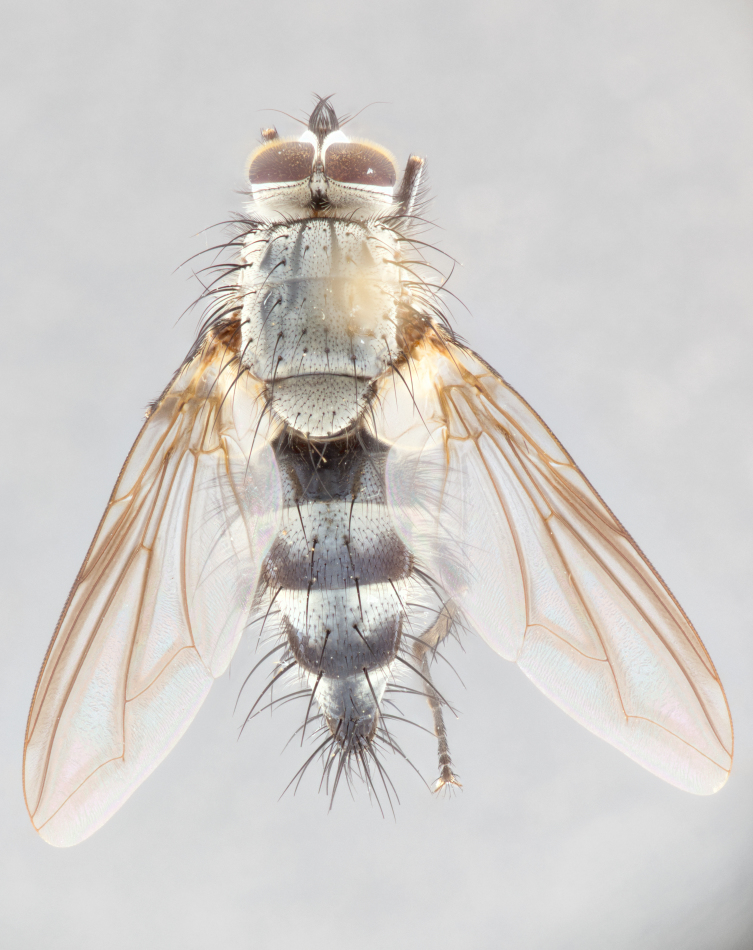
habitus in dorsal view

**Figure 9b.**
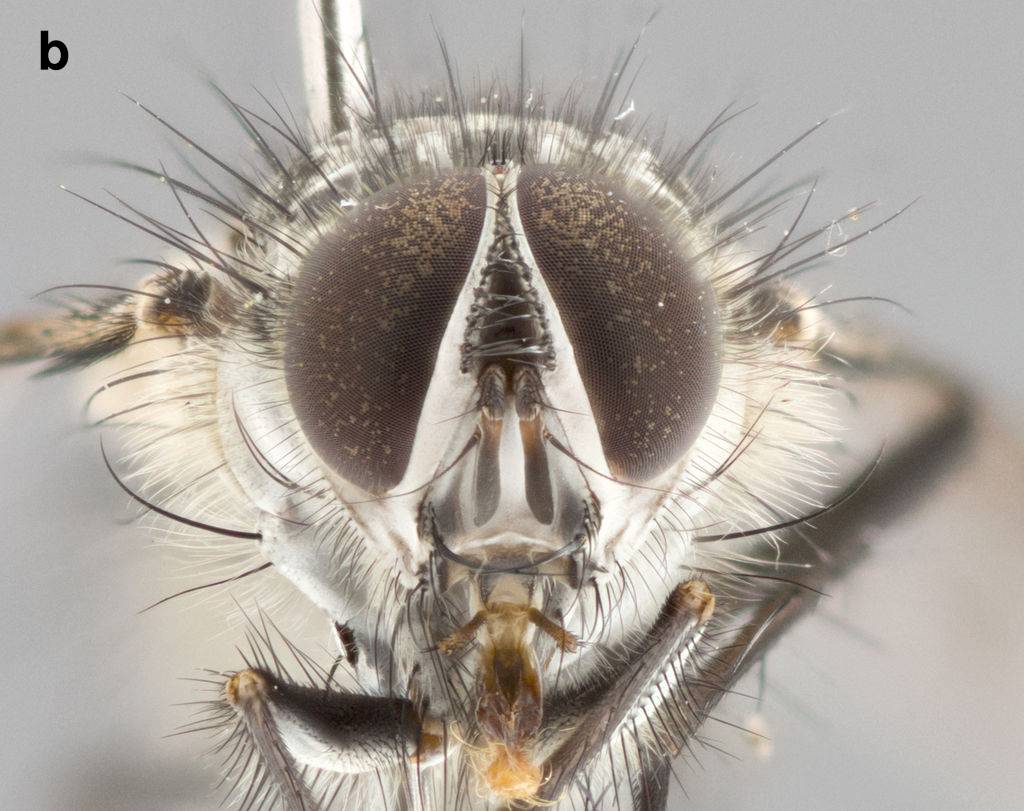
head in frontal view

**Figure 9c.**
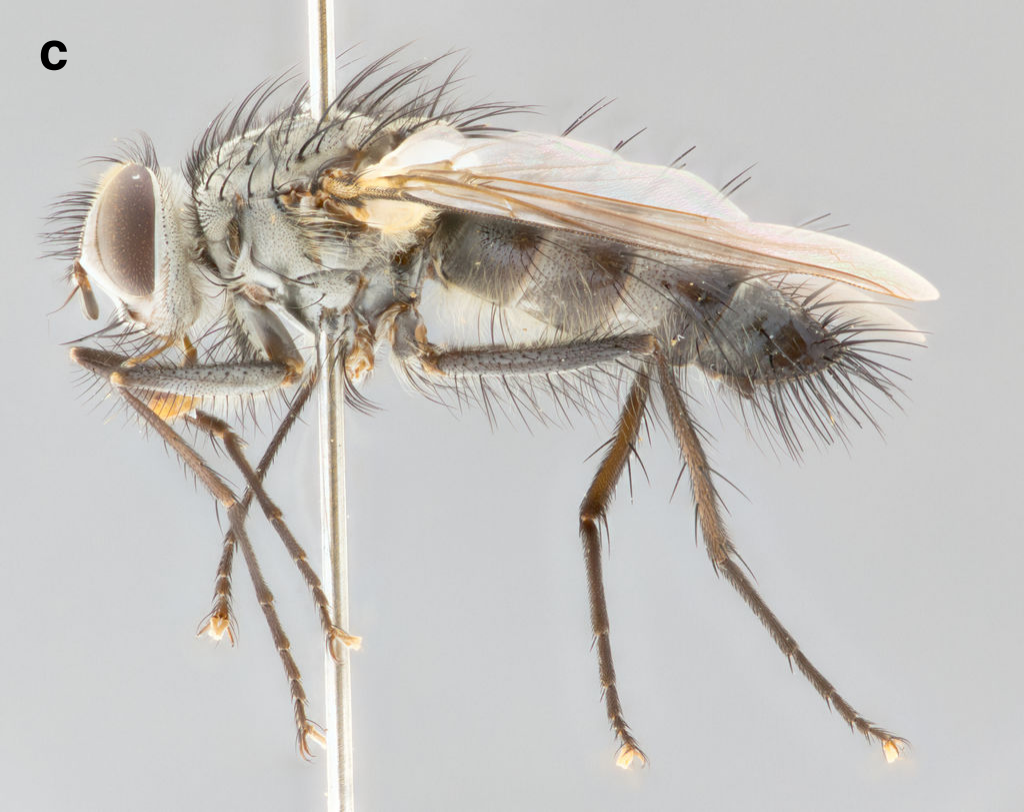
habitus in lateral view

**Figure 9d.**
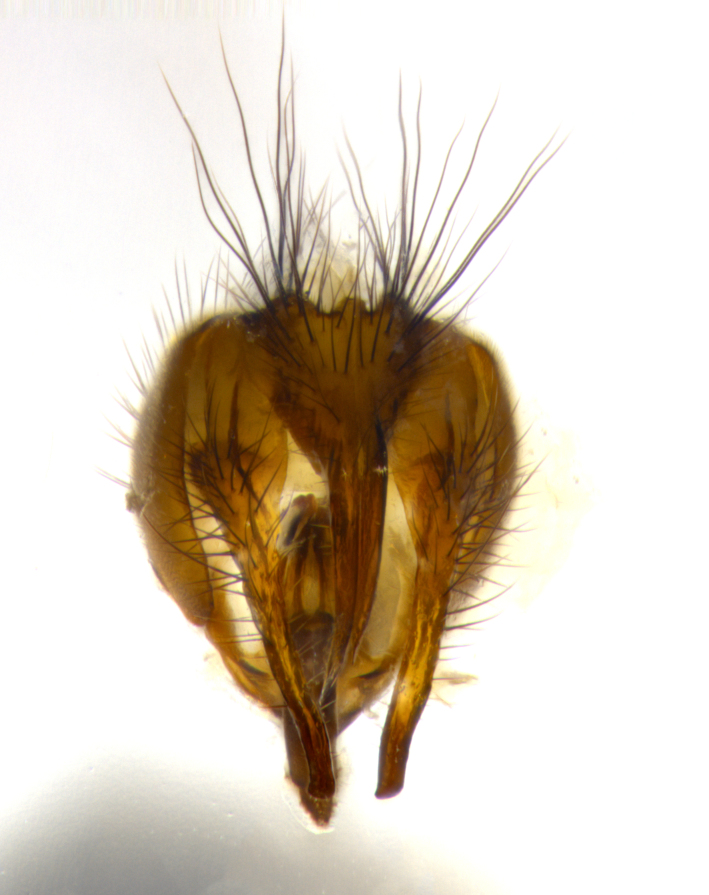
terminalia in dorsal view

**Figure 9e.**
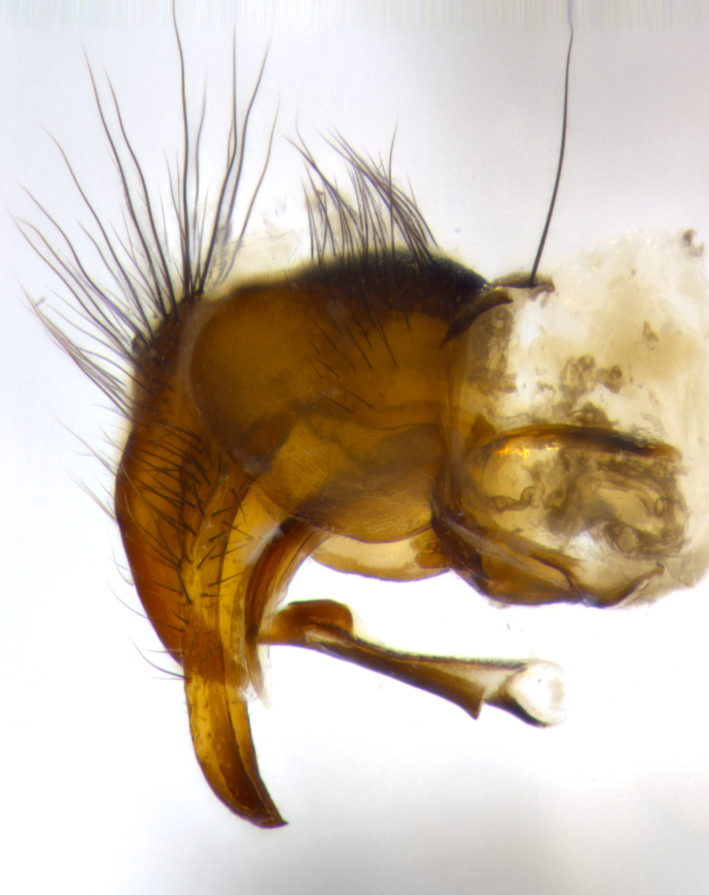
terminalia in lateral view

**Figure 9f.**
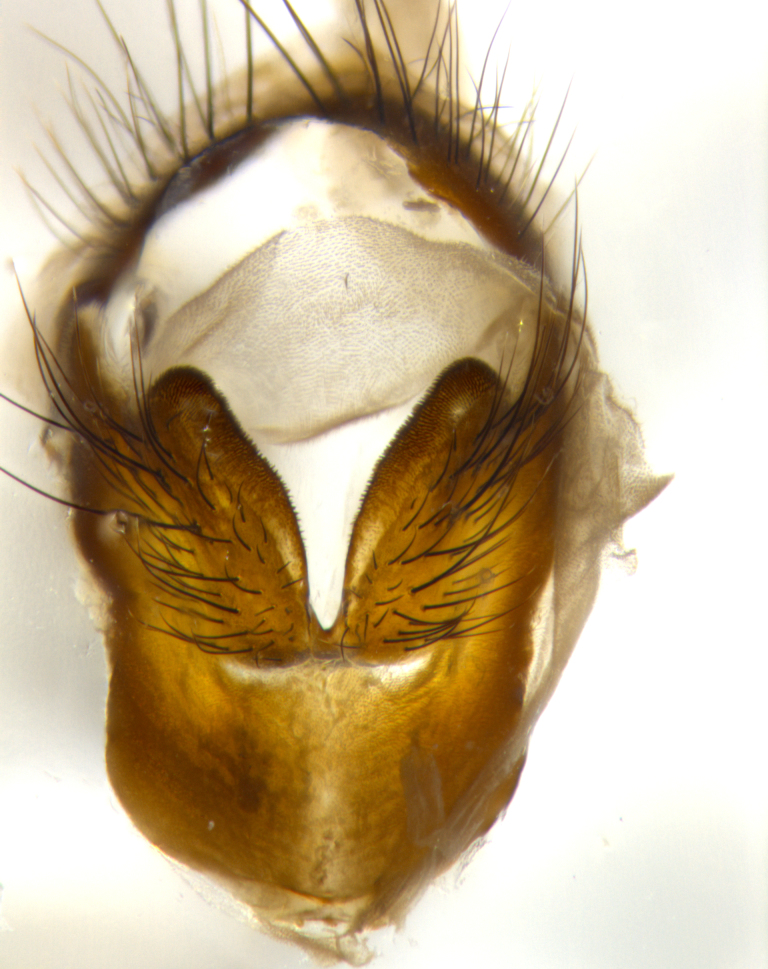
sternite 5 in ventral view

**Figure 10a.**
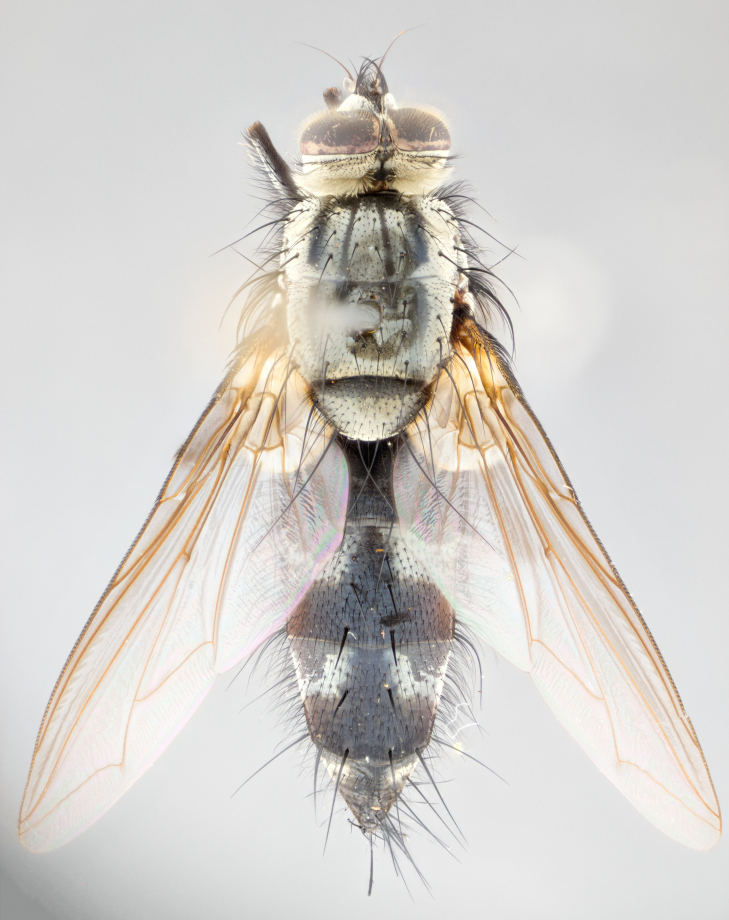
habitus in dorsal view

**Figure 10b.**
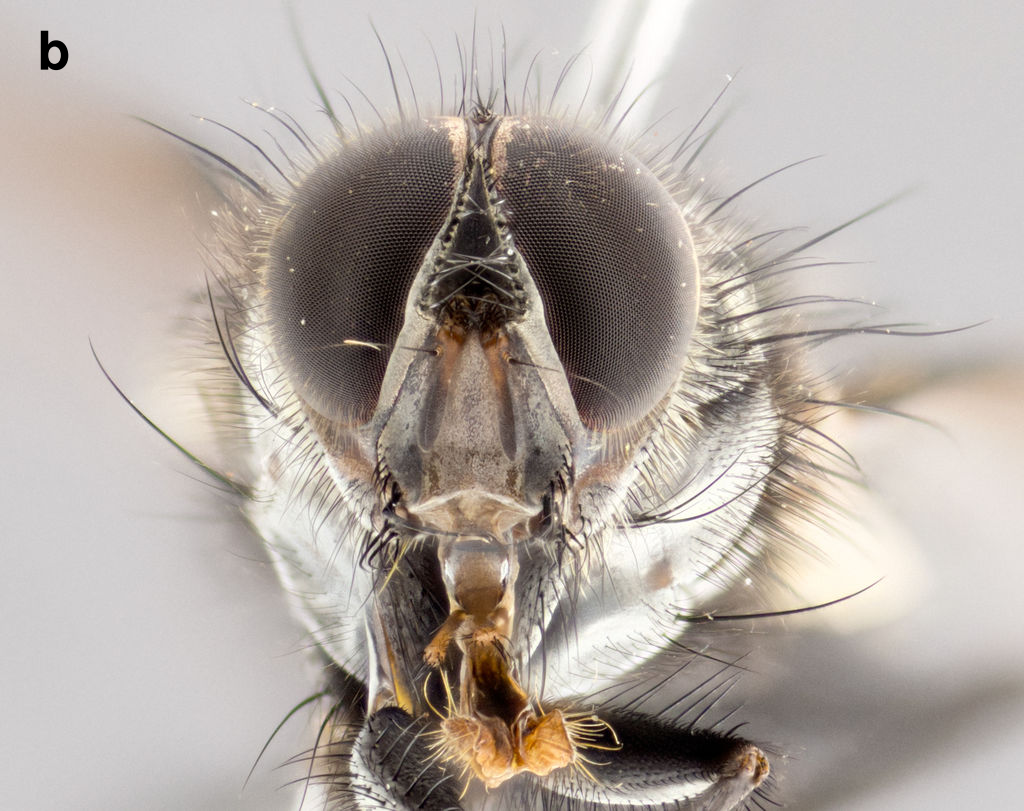
head in frontal view

**Figure 10c.**
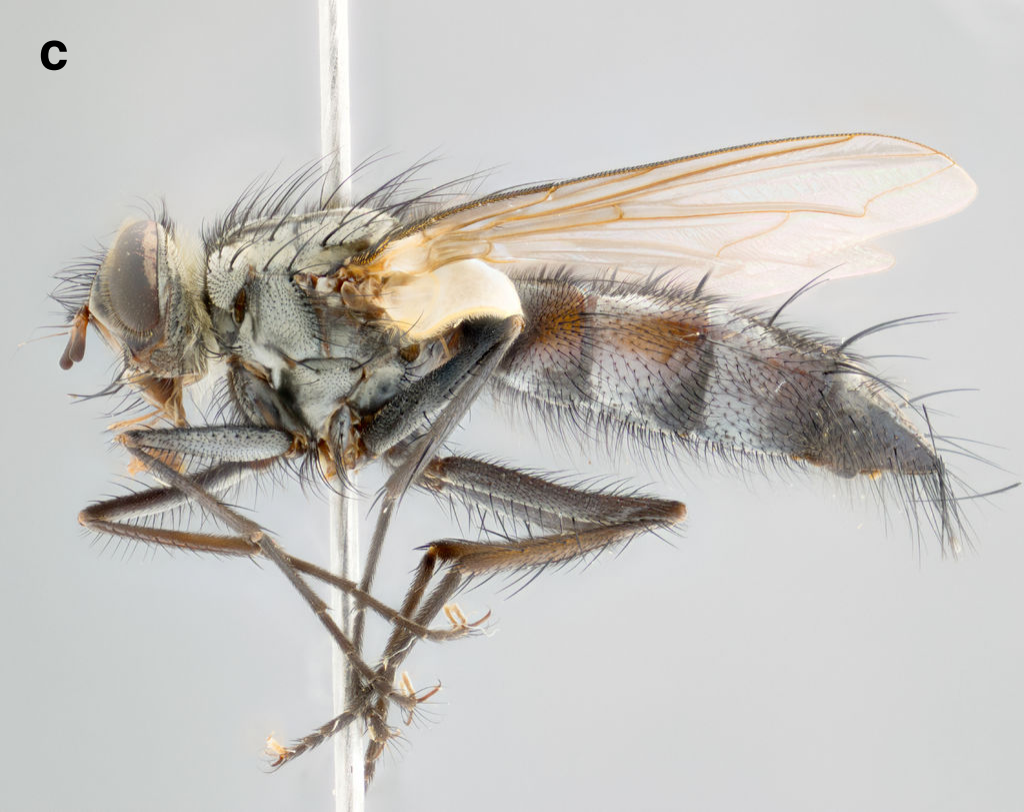
habitus in lateral view

**Figure 10d.**
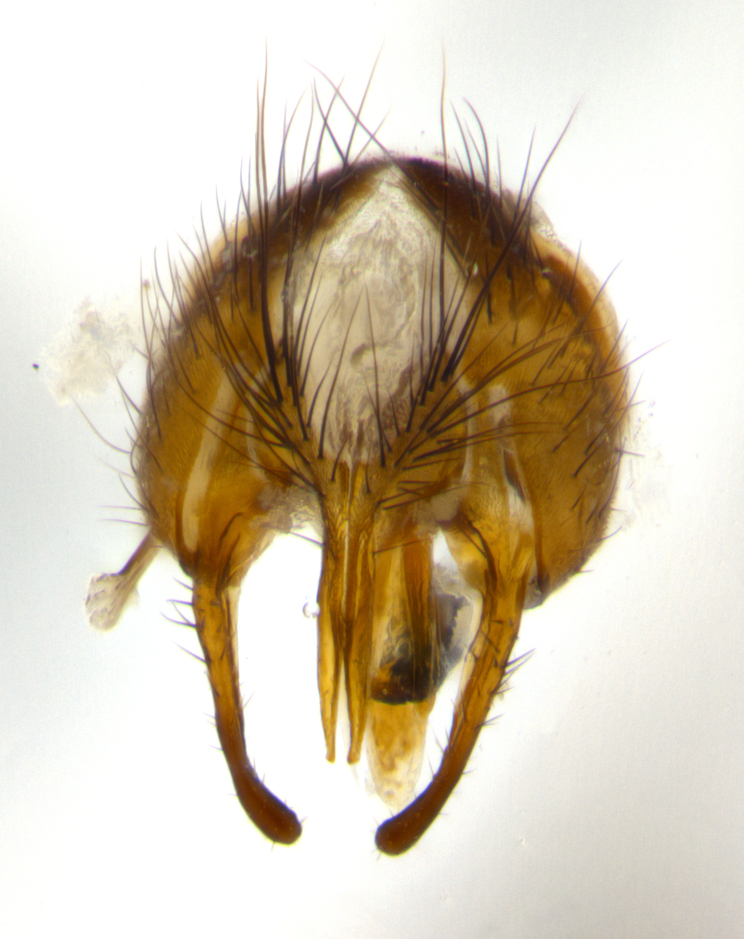
terminalia in dorsal view

**Figure 10e.**
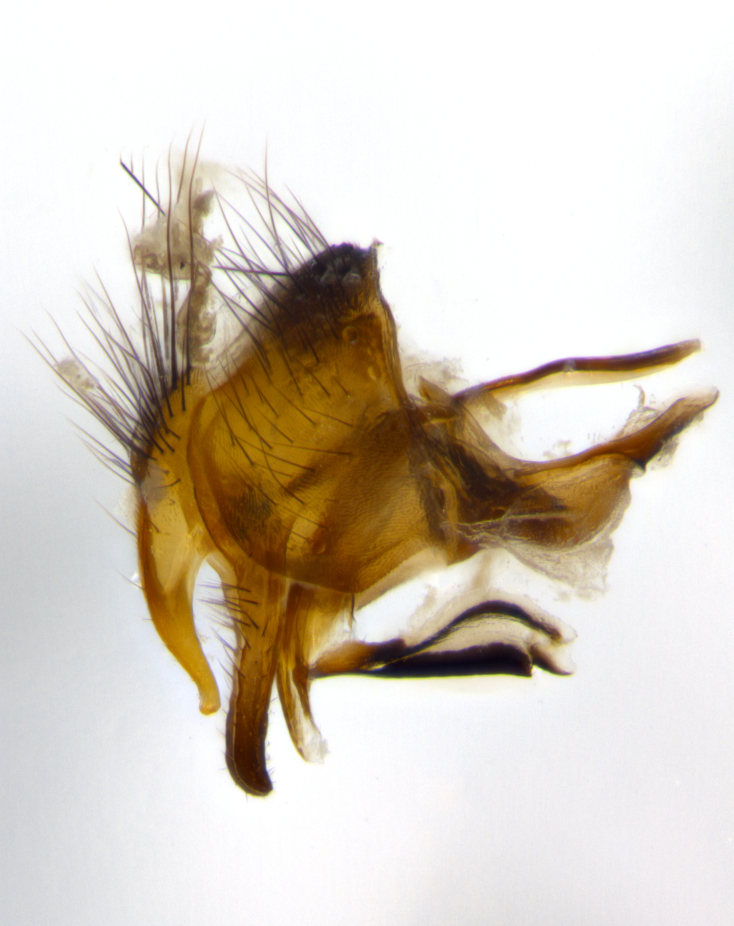
terminalia in lateral view

**Figure 10f.**
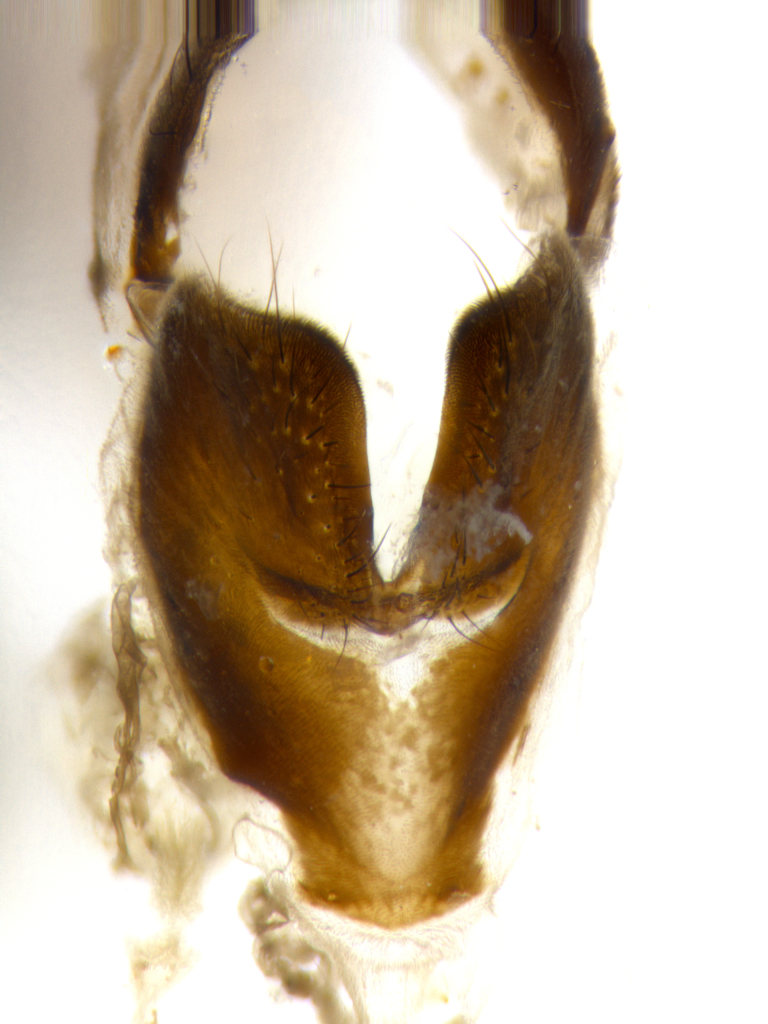
sternite 5 in ventral view

**Figure 11a.**
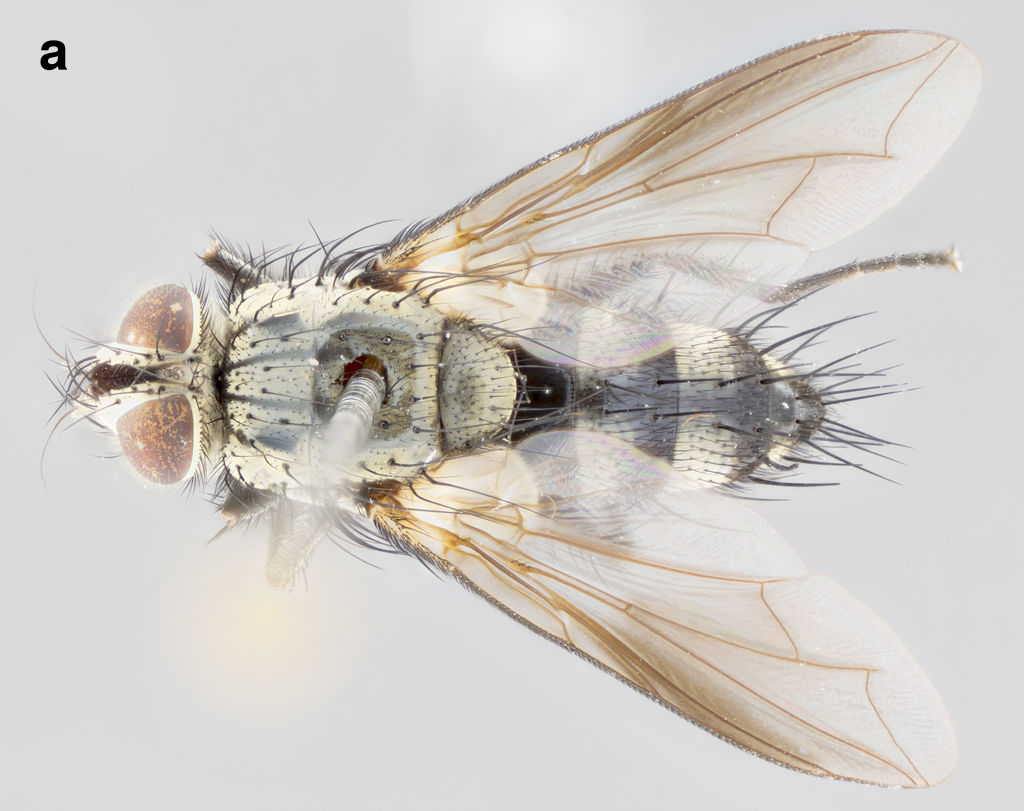
habitus in dorsal view

**Figure 11b.**
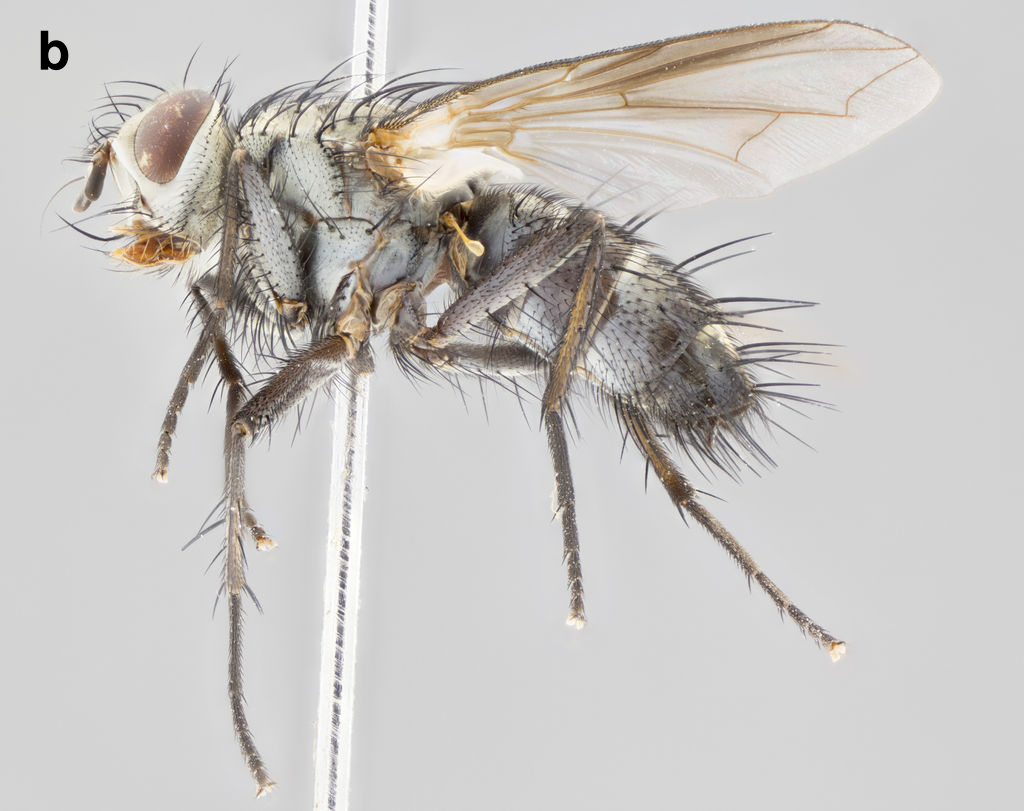
habitus in lateral view

**Figure 12a.**
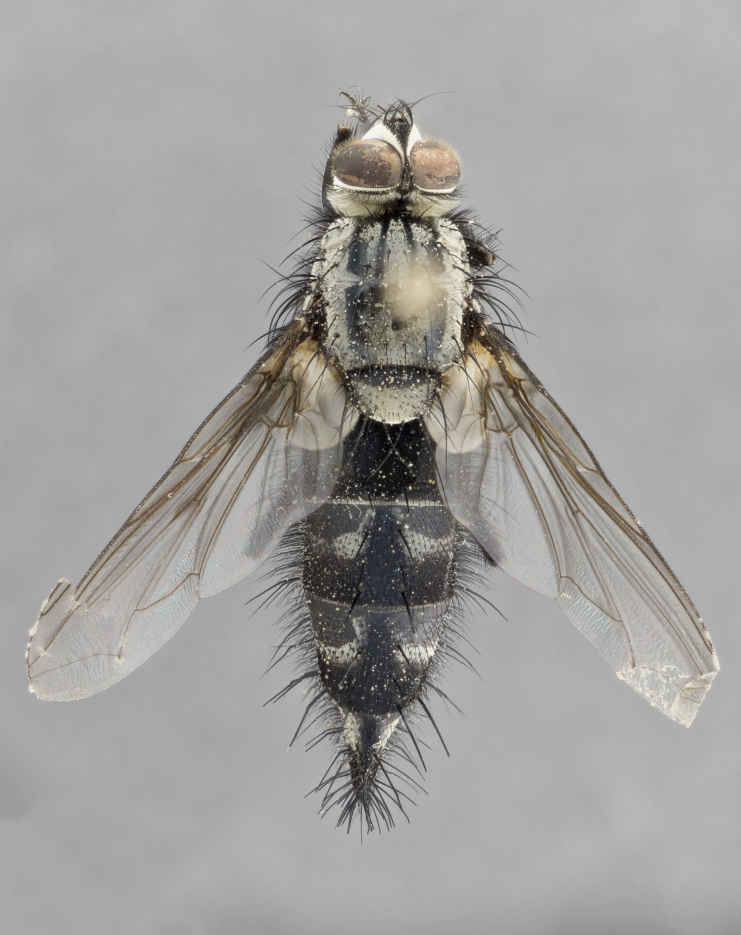
habitus in dorsal view

**Figure 12b.**
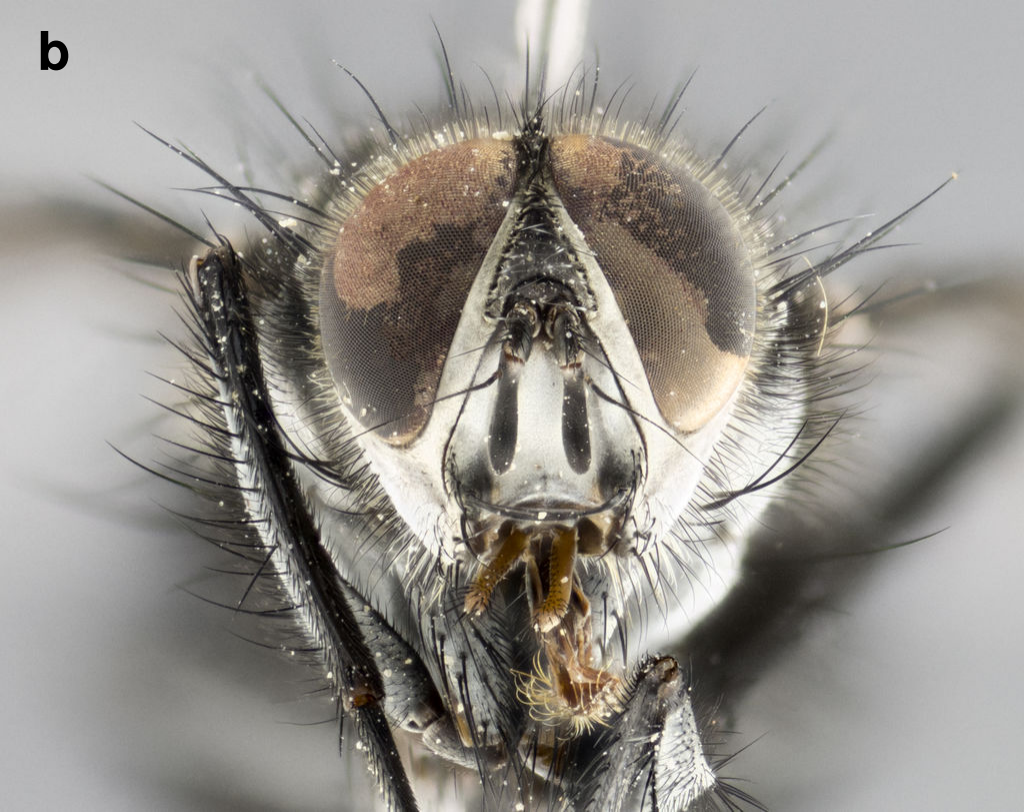
head in frontal view

**Figure 12c.**
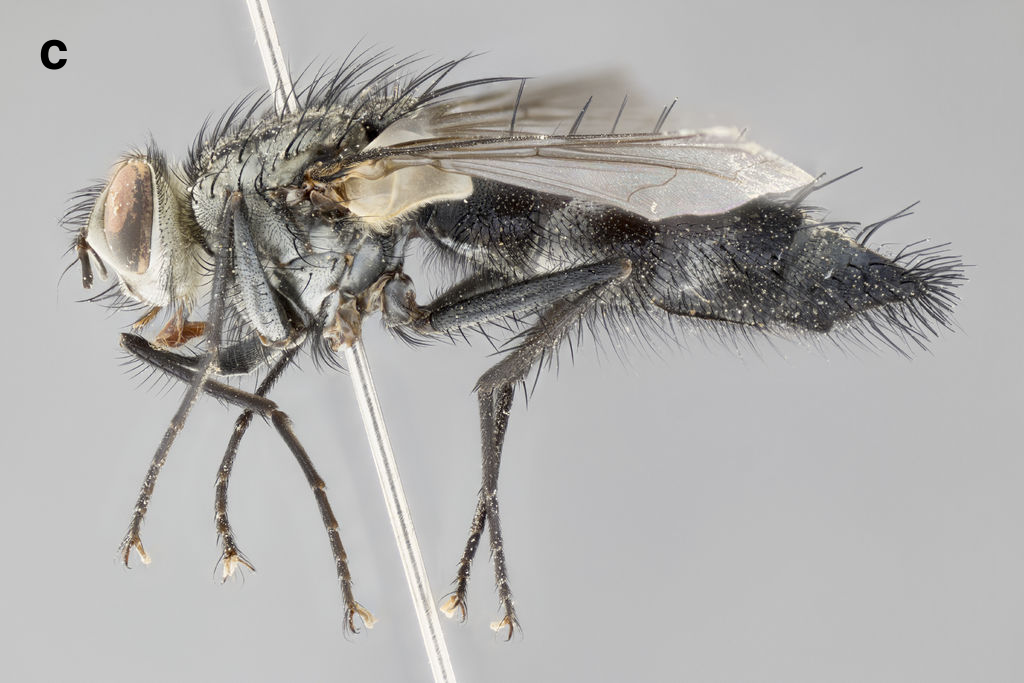
habitus in lateral view

**Figure 13a.**
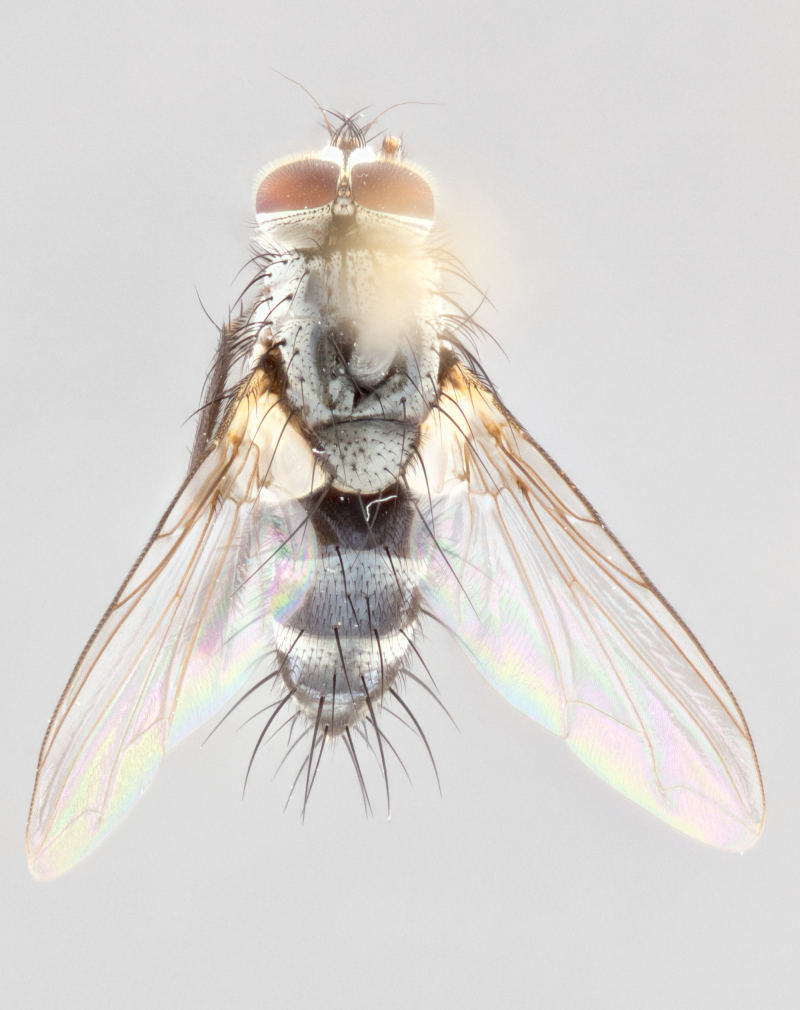
habitus in dorsal view

**Figure 13b.**
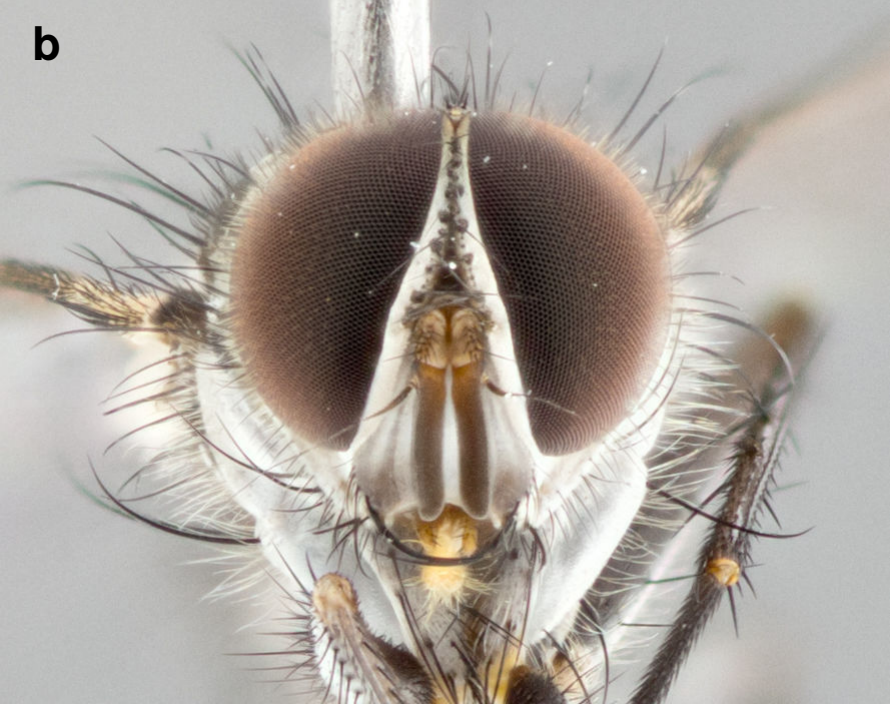
head in frontal view

**Figure 13c.**
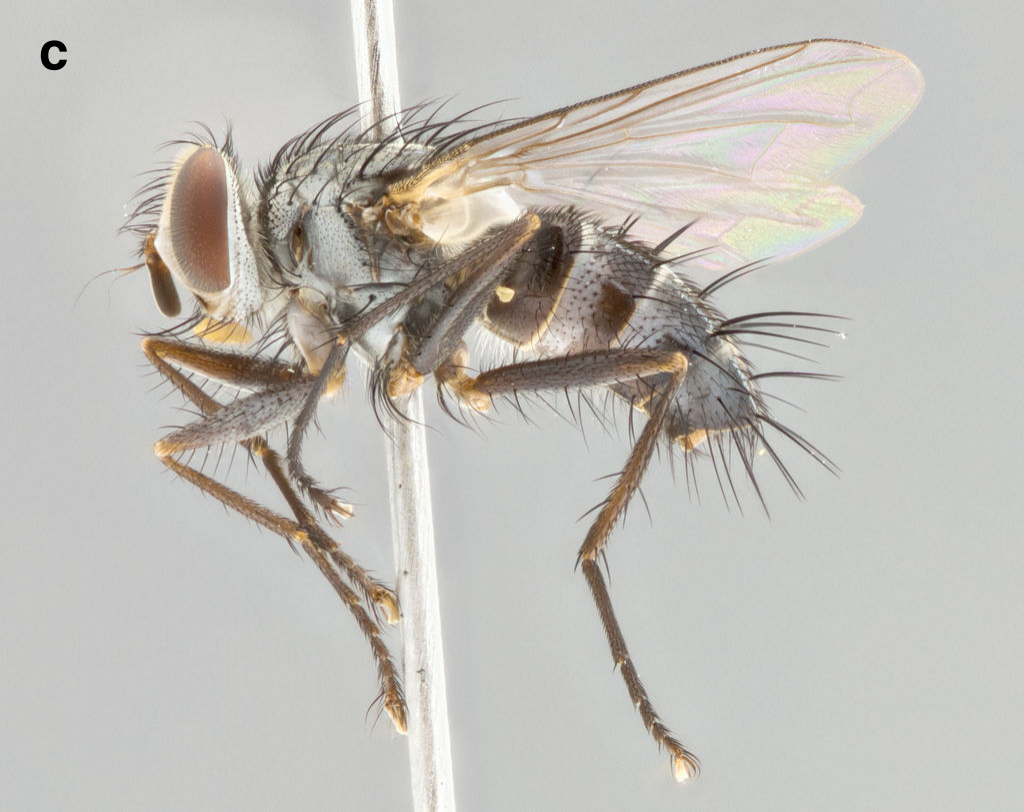
habitus in lateral view

**Figure 13d.**
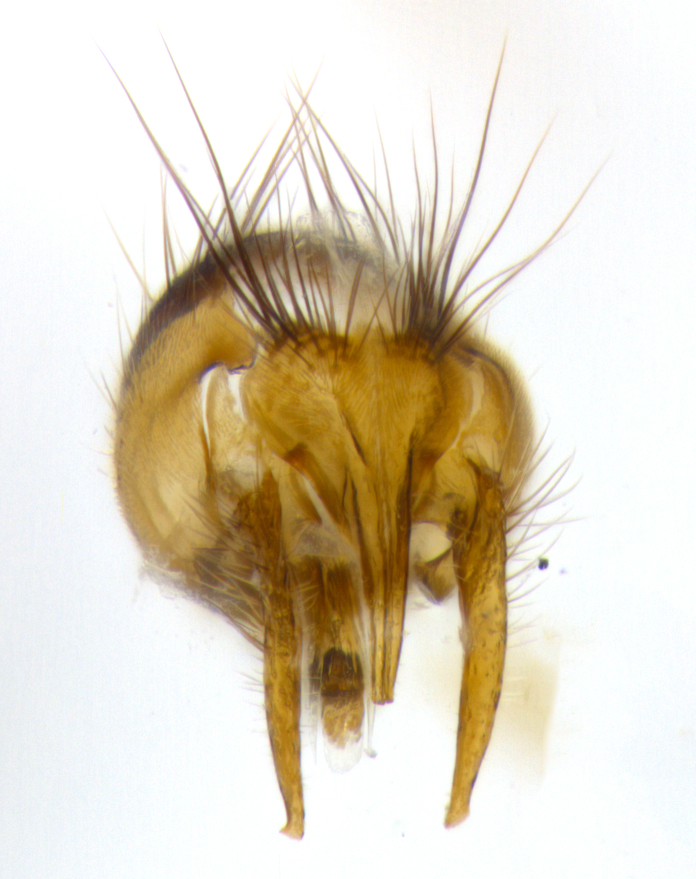
terminalia in dorsal view

**Figure 13e.**
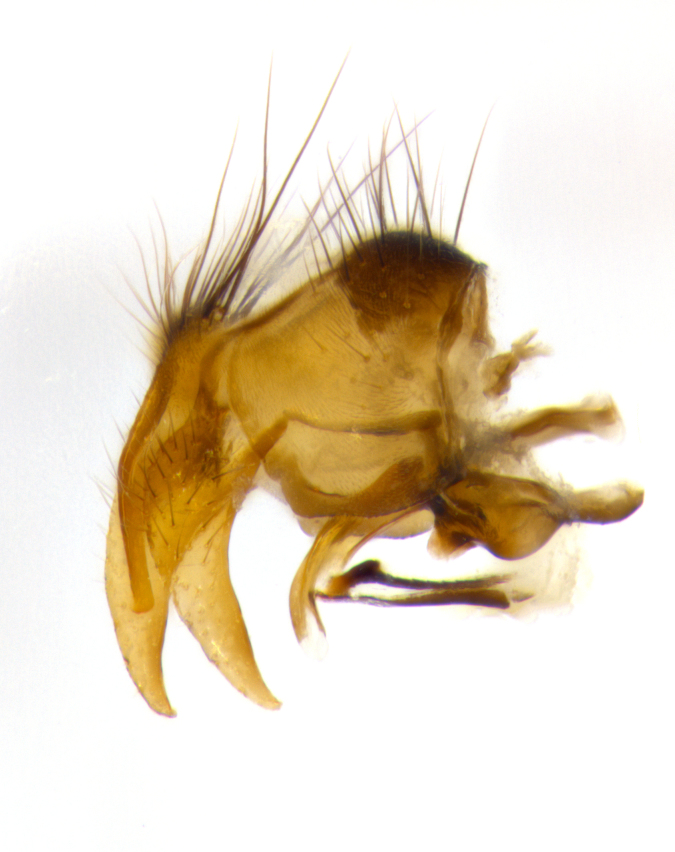
terminalia in lateral view

**Figure 13f.**
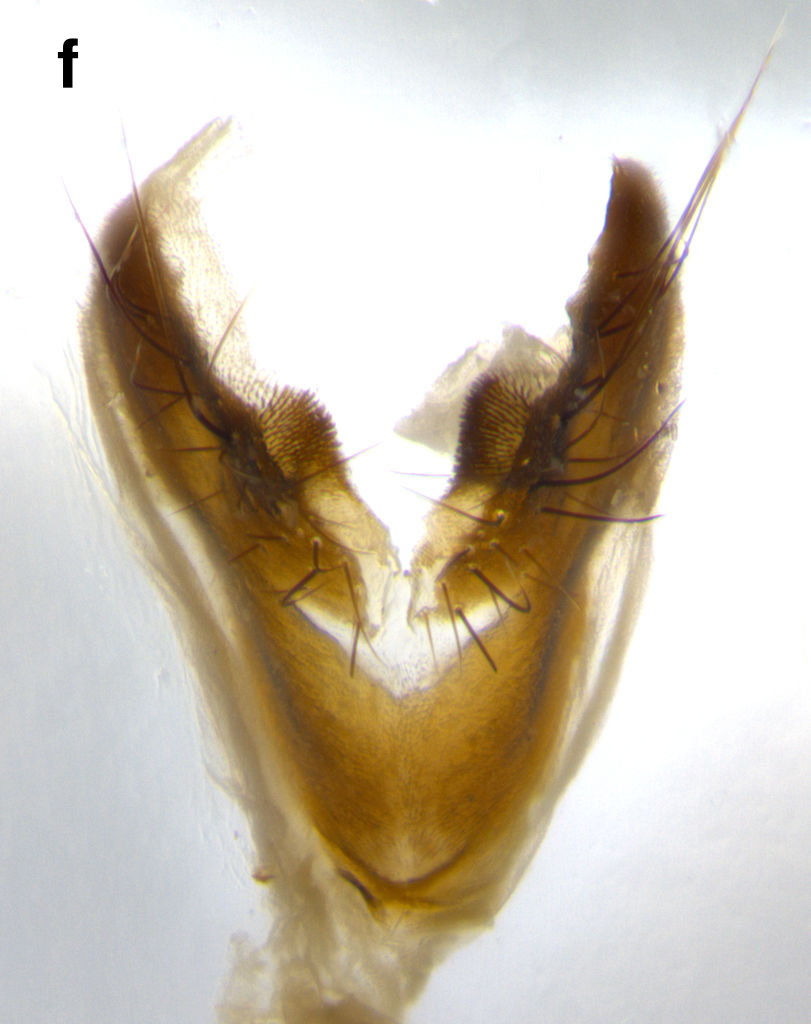
sternite 5 in ventral view

**Figure 14a.**
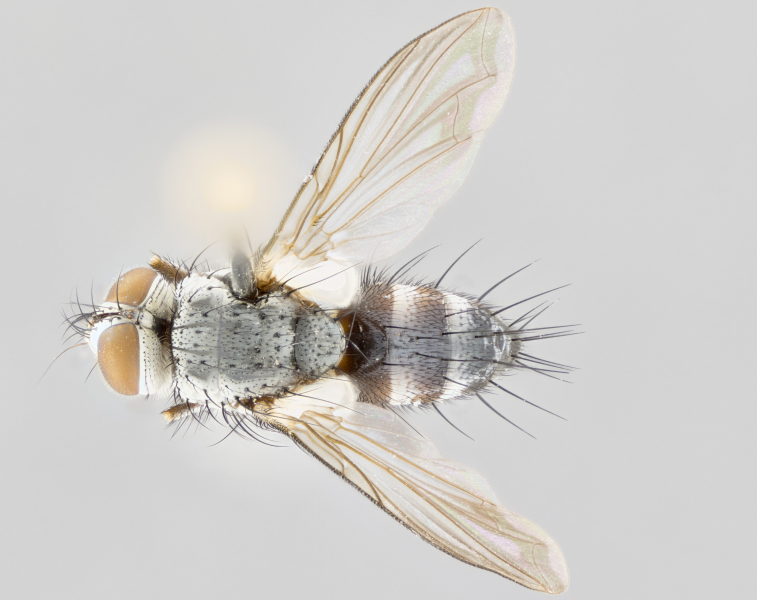
habitus in dorsal view

**Figure 14b.**
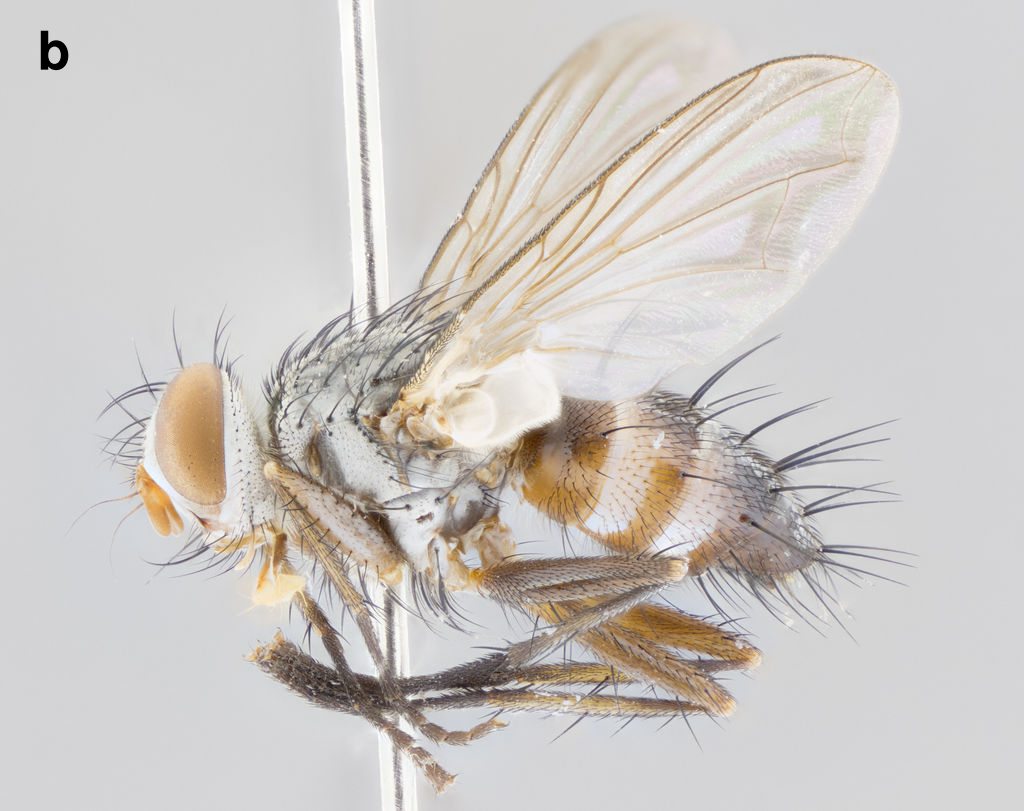
habitus in lateral view

**Figure 15a.**
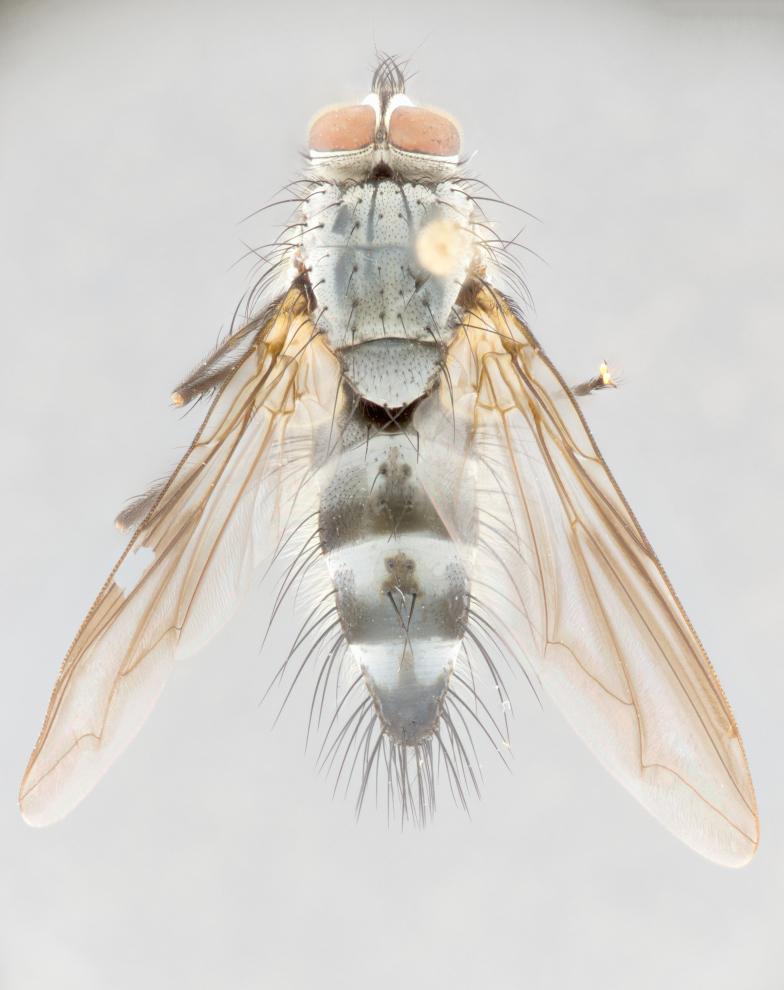
habitus in dorsal view

**Figure 15b.**
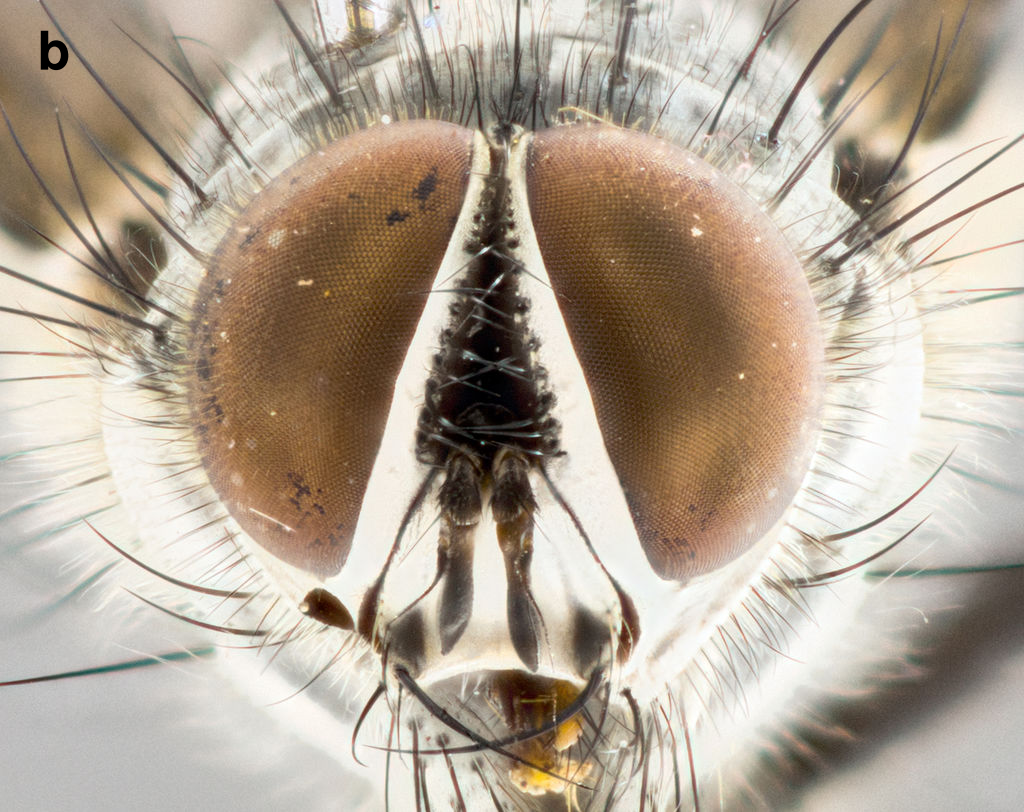
head in frontal view

**Figure 15c.**
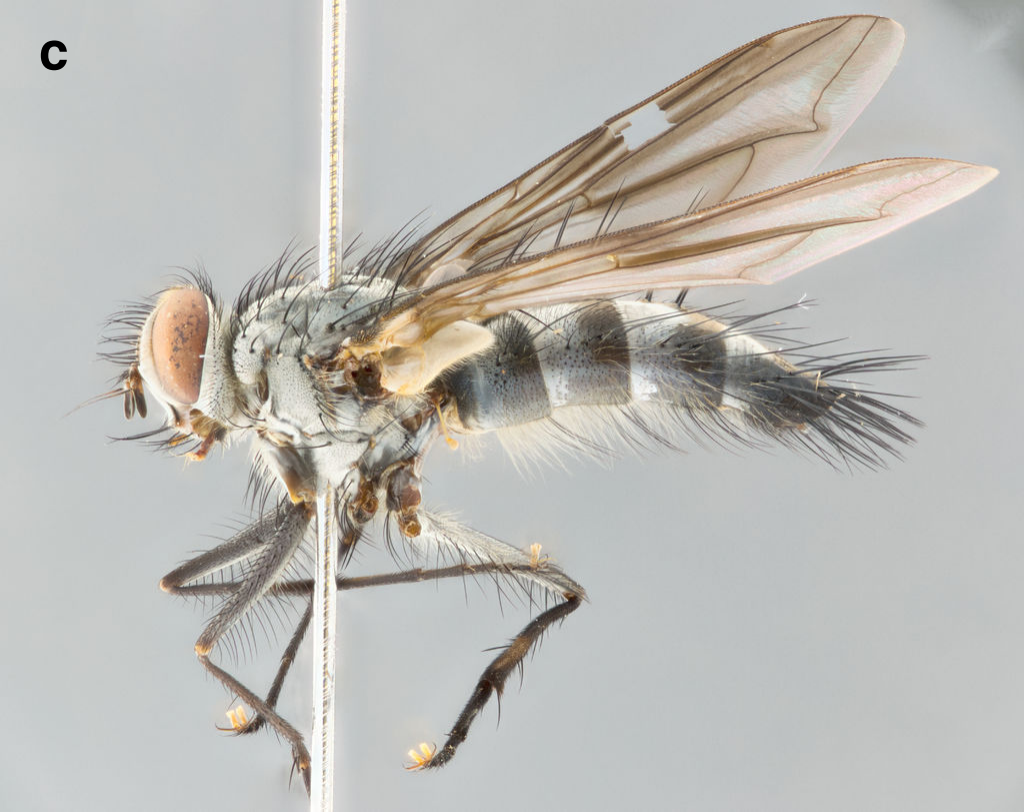
habitus in lateral view

**Figure 15d.**
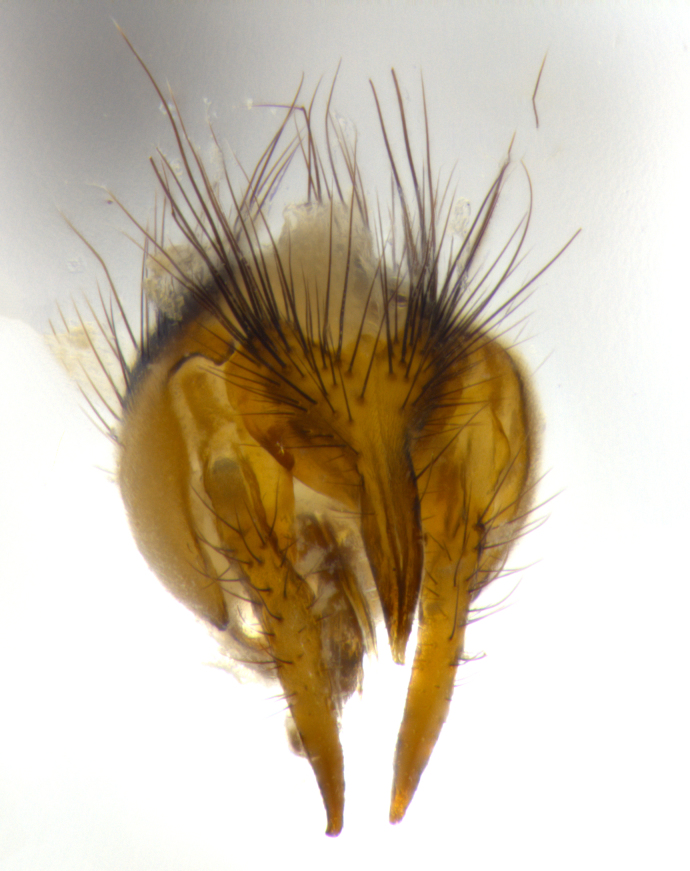
terminalia in dorsal view

**Figure 15e.**
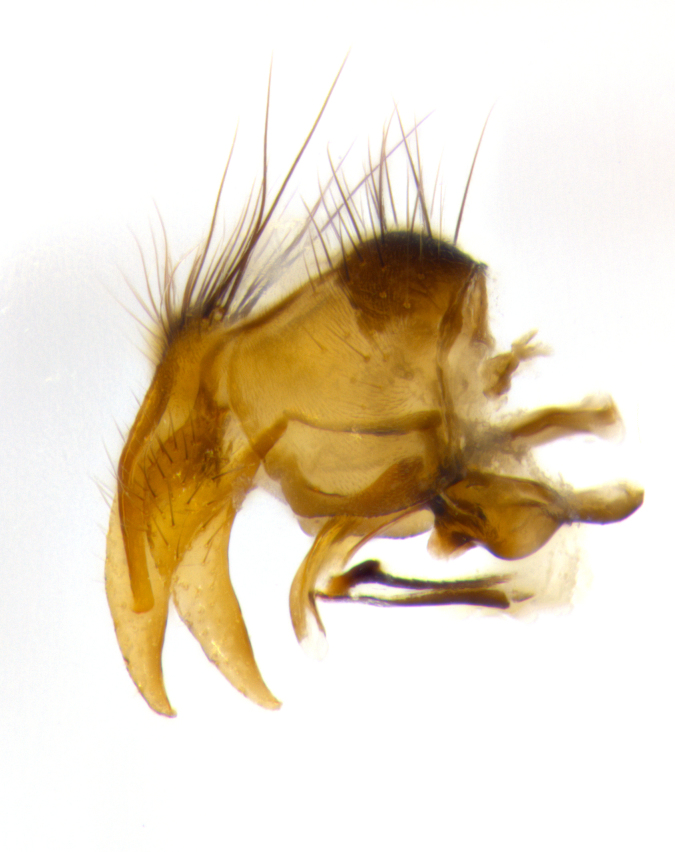
terminalia in lateral view

**Figure 15f.**
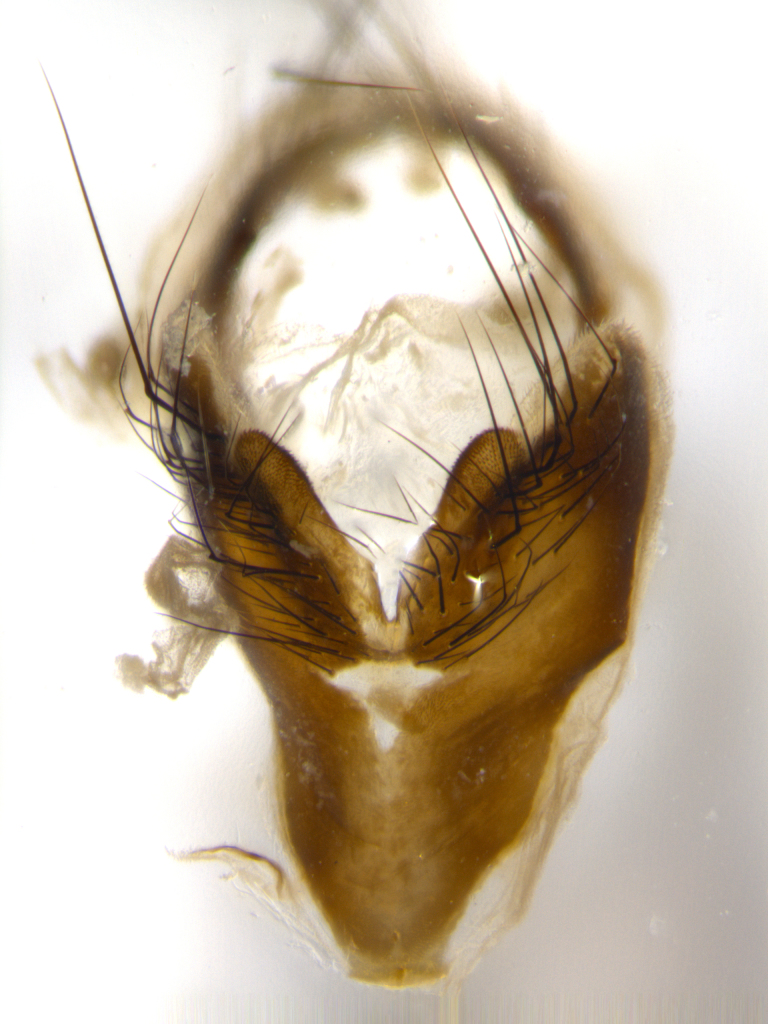
sternite 5 in ventral view

**Figure 16a.**
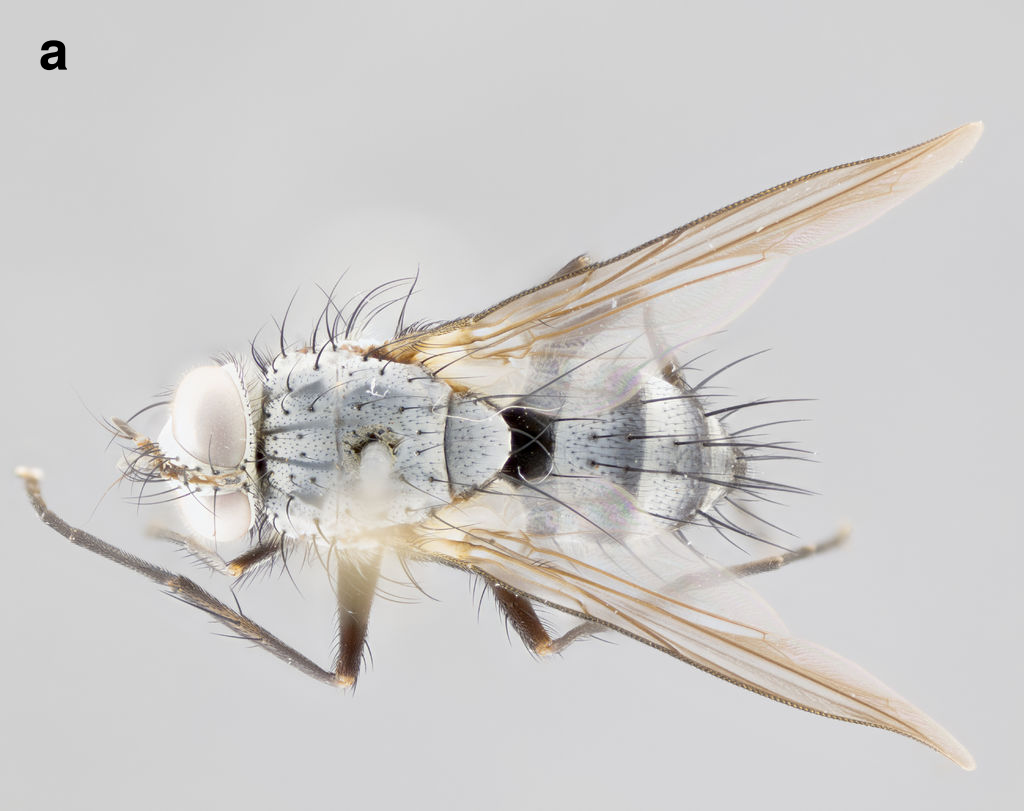
habitus in dorsal view

**Figure 16b.**
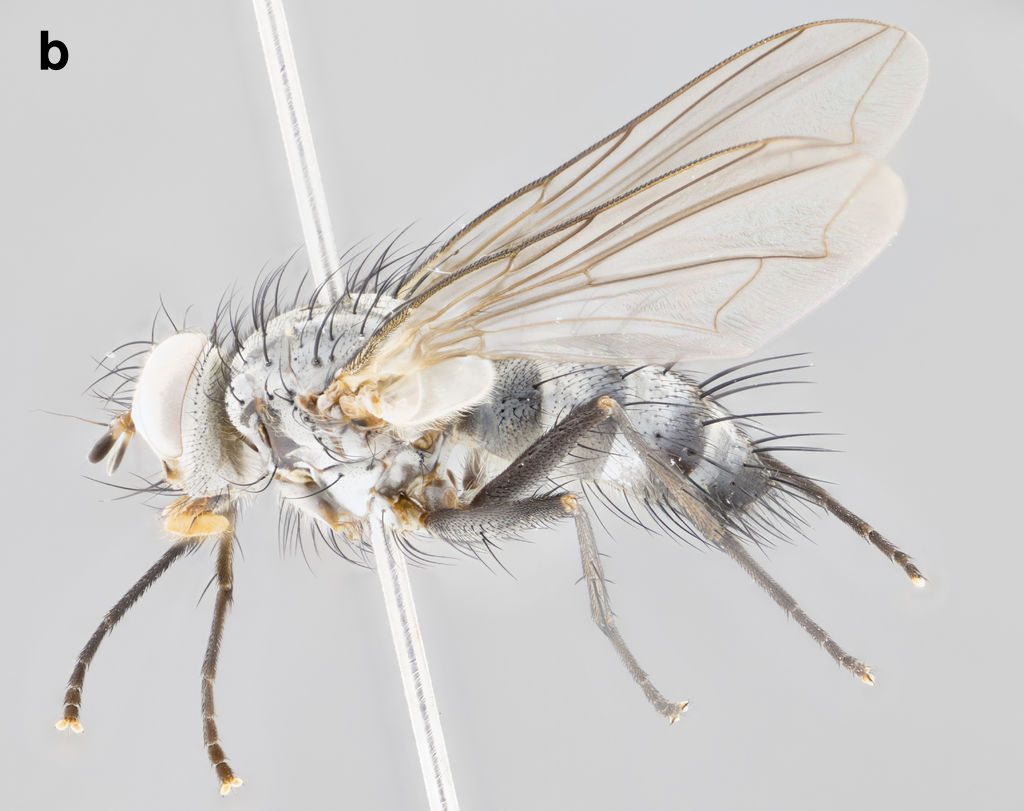
habitus in lateral view

**Figure 17a.**
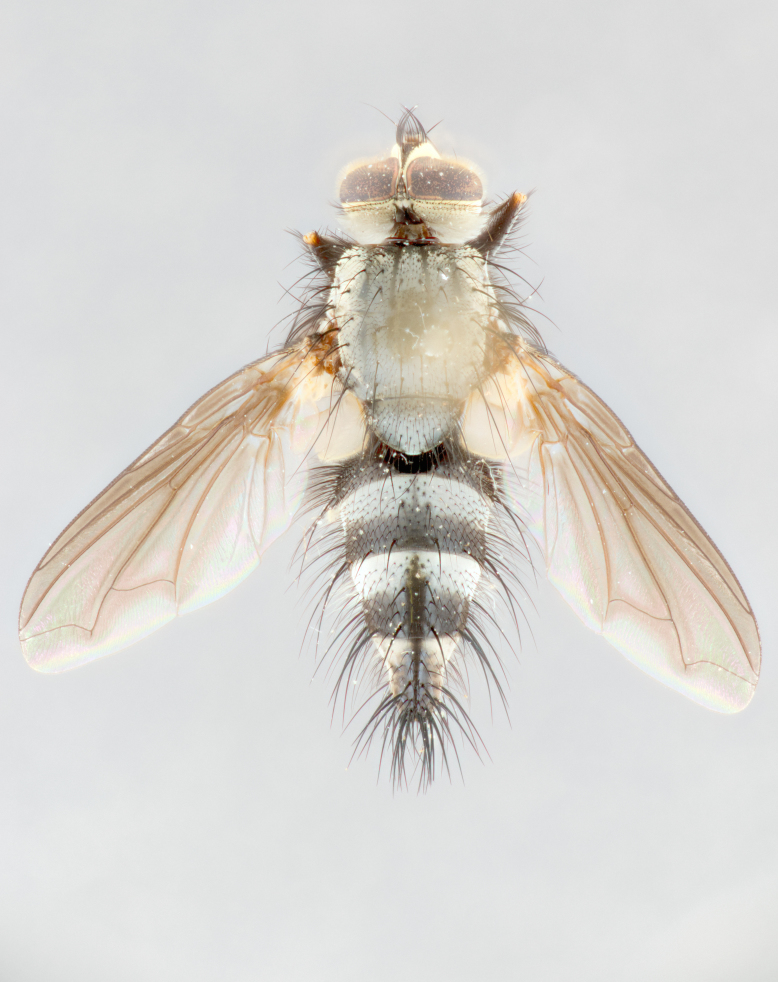
habitus in dorsal view

**Figure 17b.**
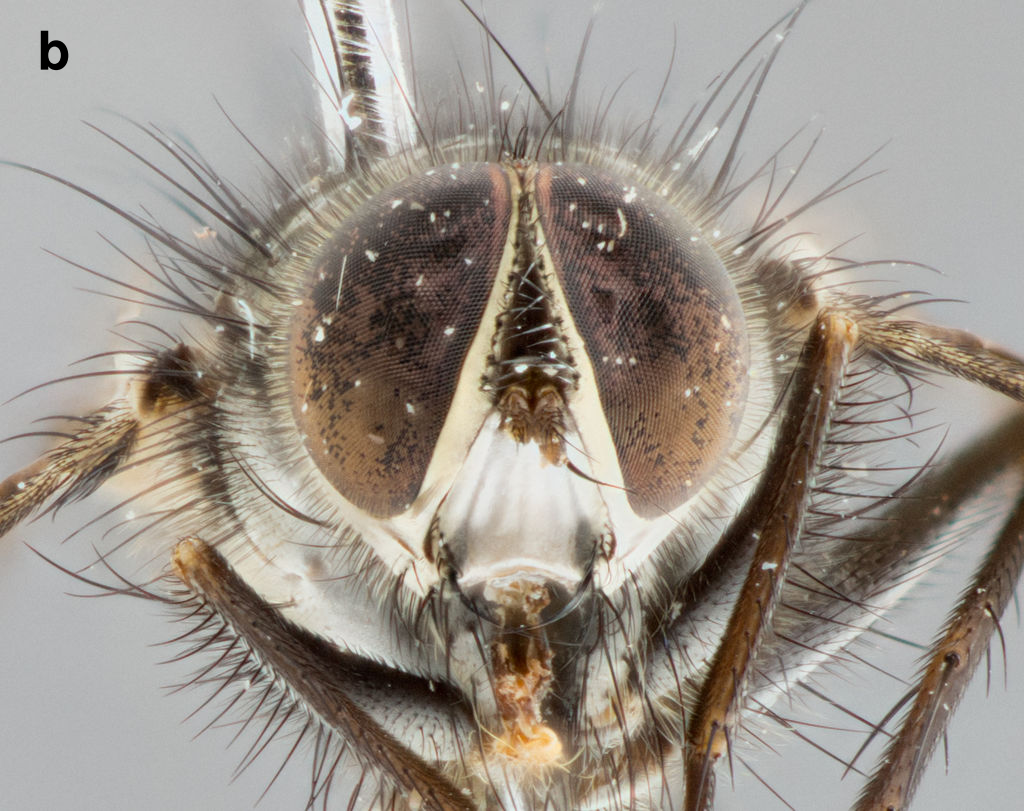
head in frontal view

**Figure 17c.**
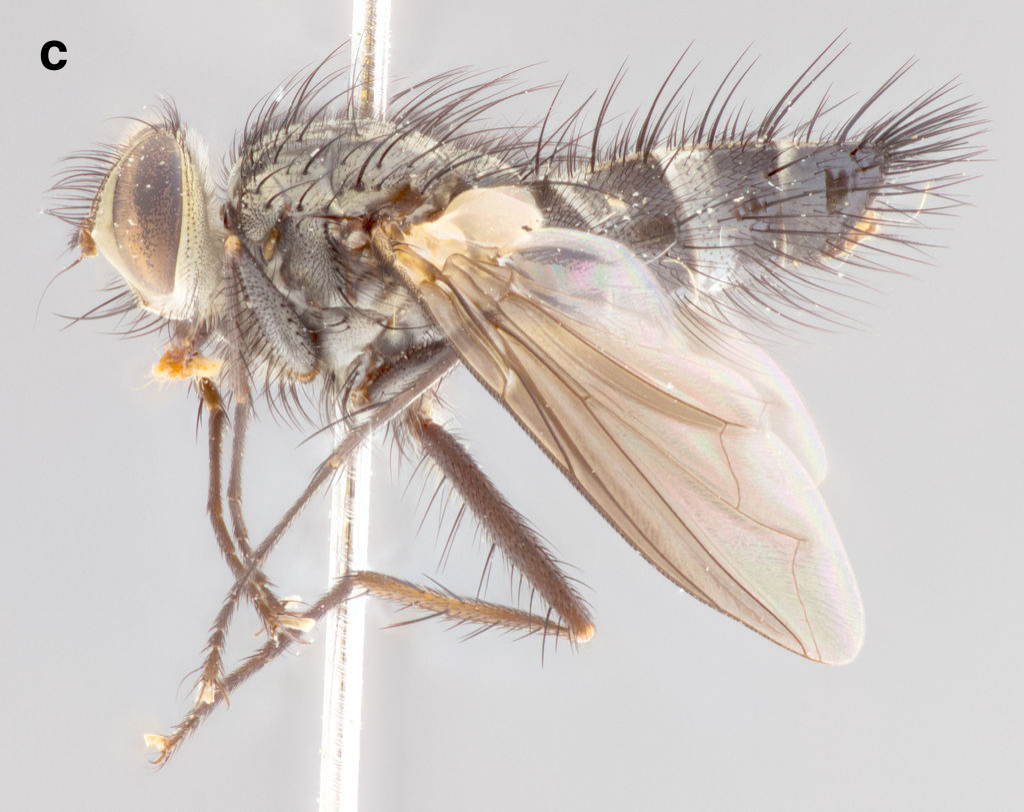
habitus in lateral view

**Figure 18a.**
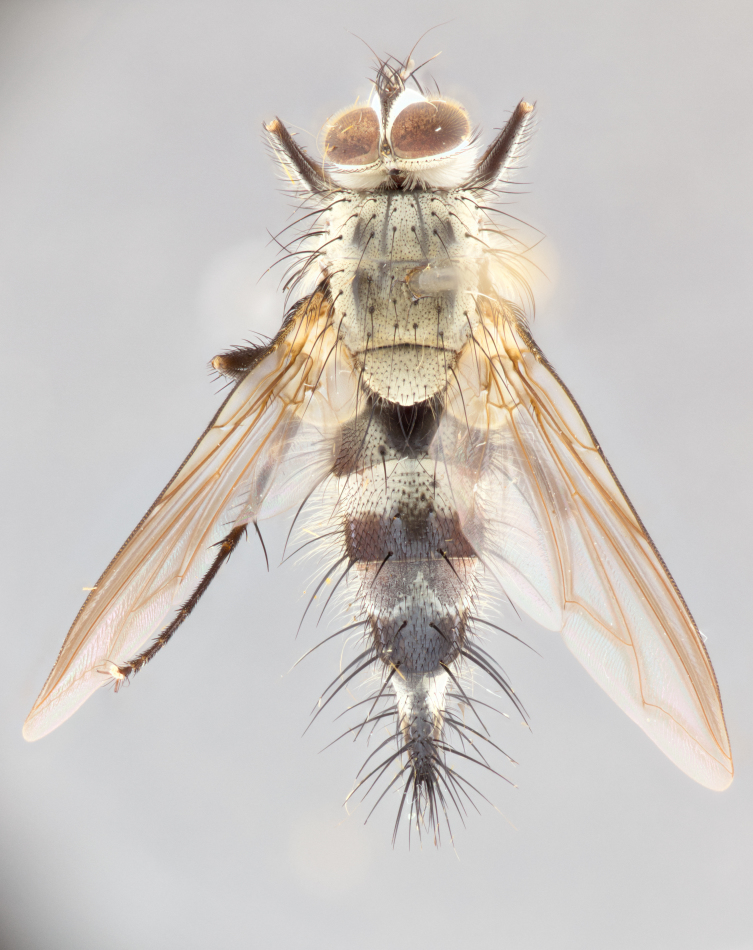
habitus in dorsal view

**Figure 18b.**
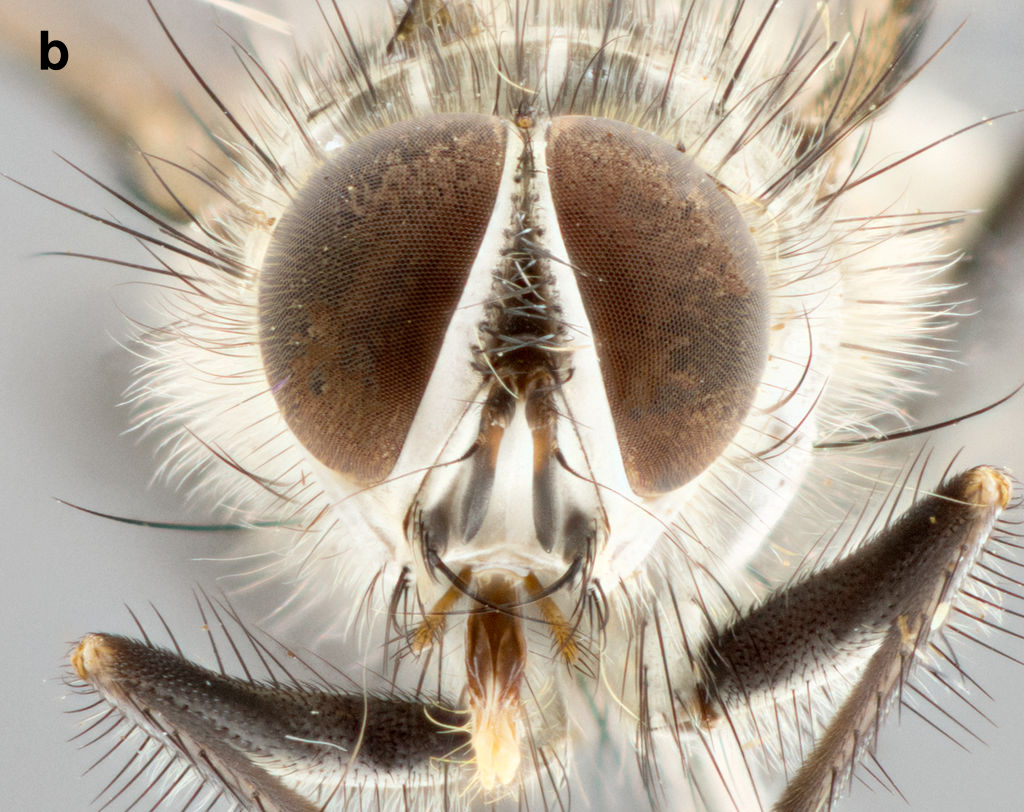
head in frontal view

**Figure 18c.**
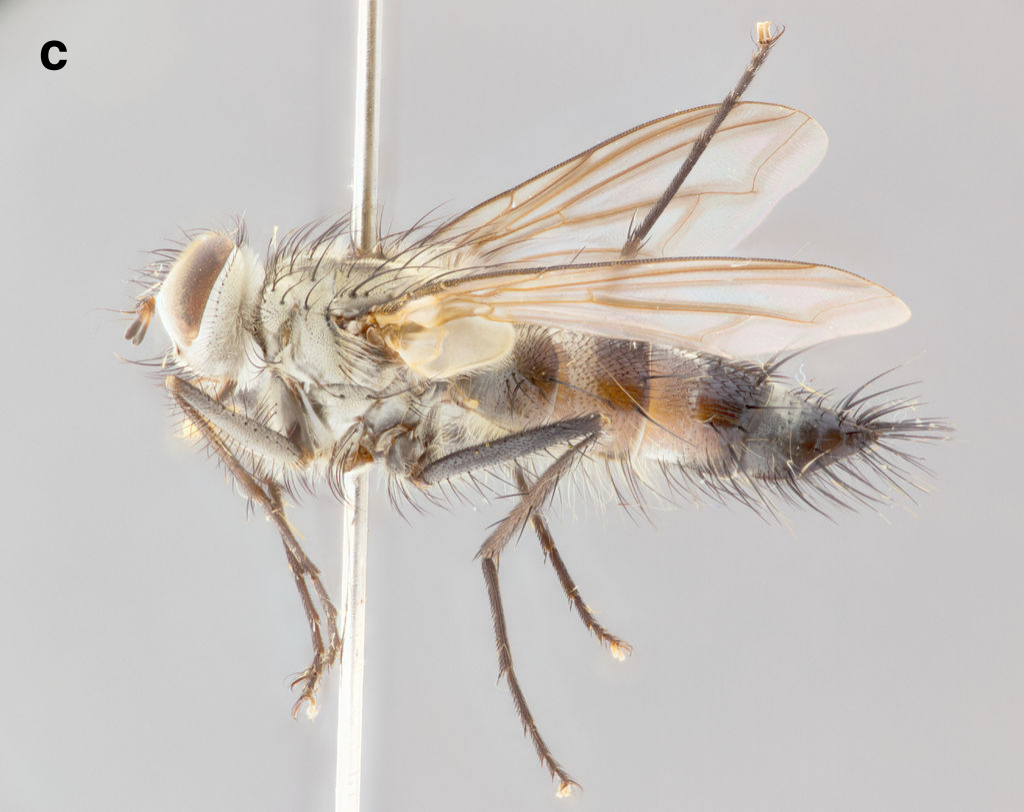
habitus in lateral view

**Figure 18d.**
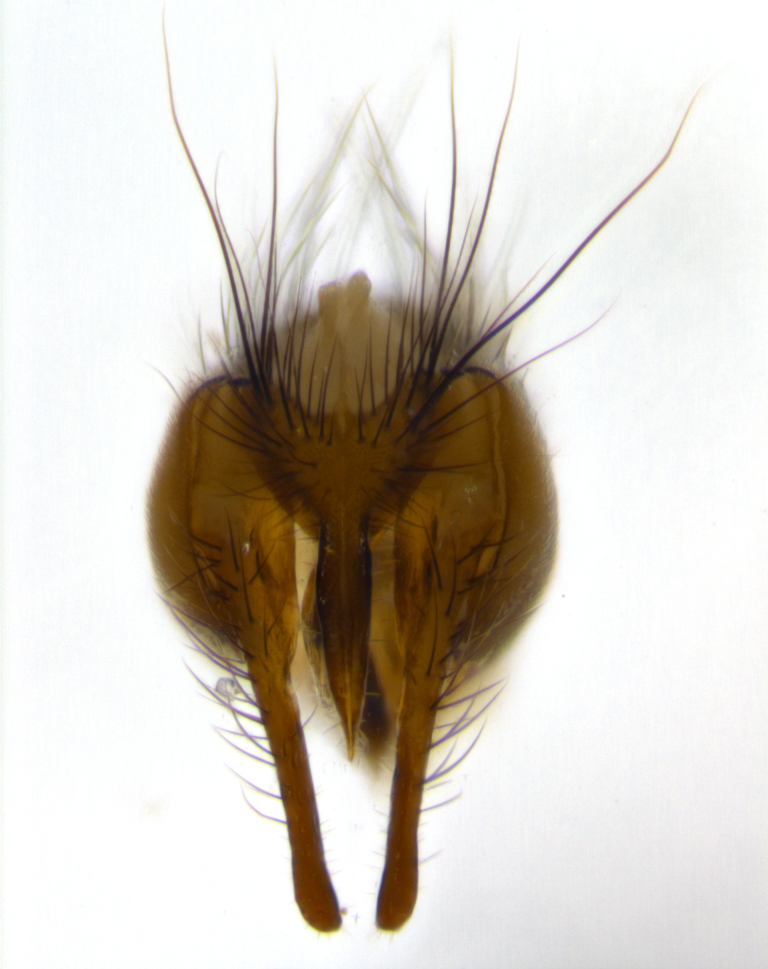
terminalia in dorsal view

**Figure 18e.**
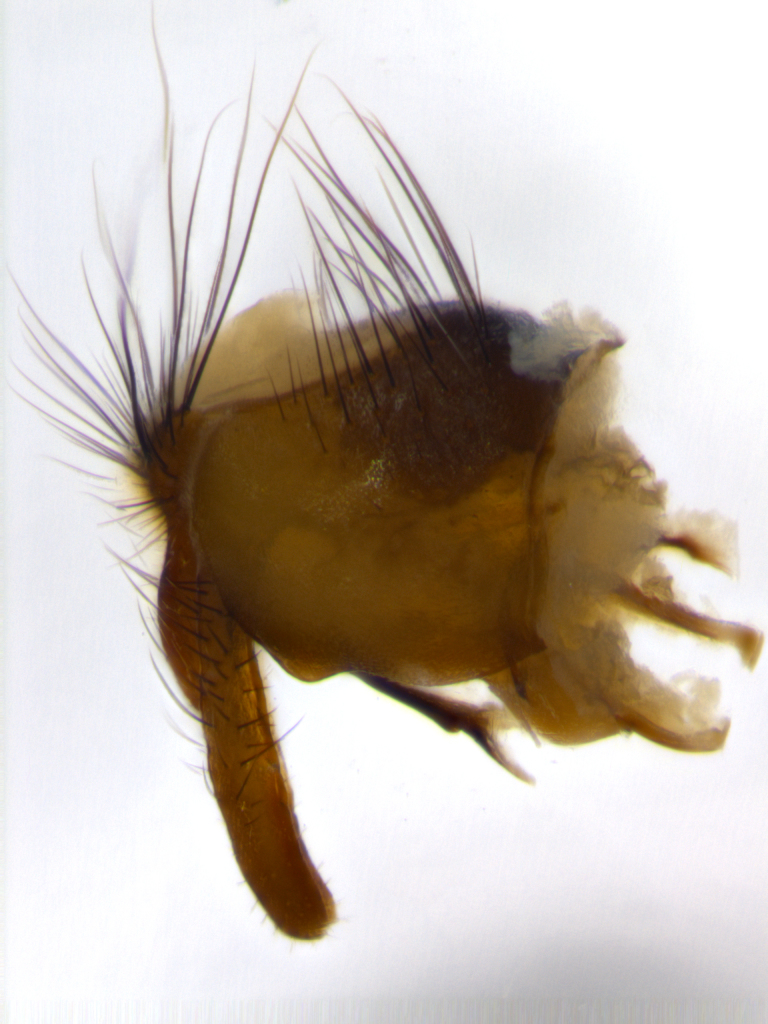
terminalia in lateral view

**Figure 18f.**
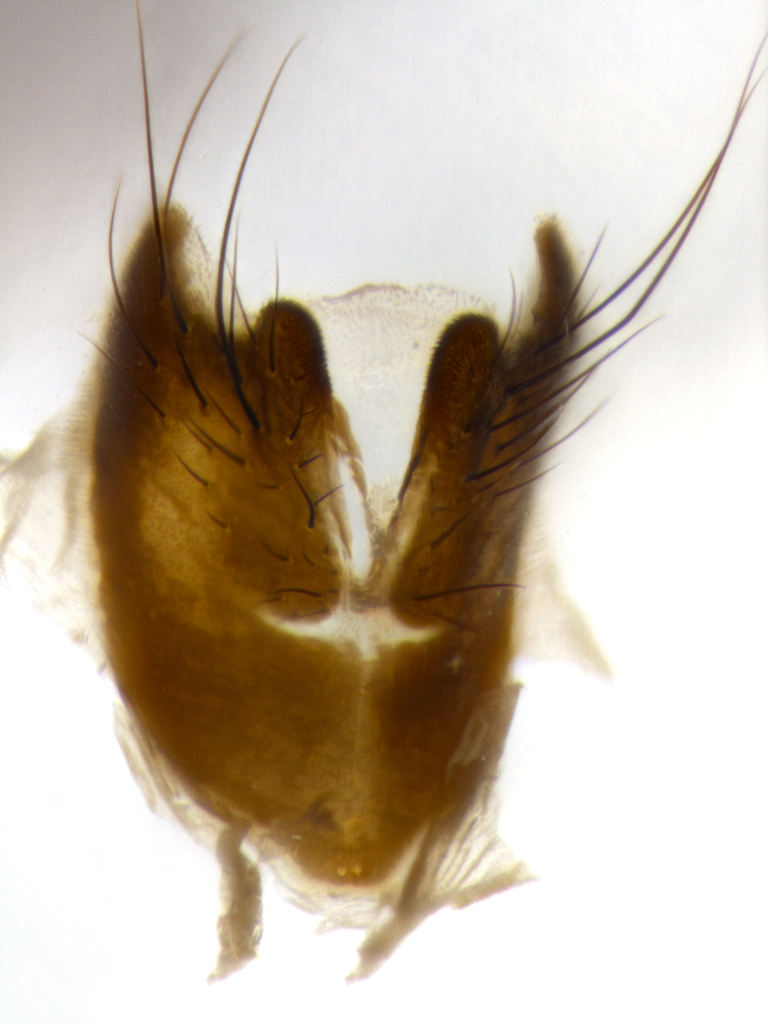
sternite 5 in ventral view
